# Accelerated evolution of SARS-CoV-2 in free-ranging white-tailed deer

**DOI:** 10.1038/s41467-023-40706-y

**Published:** 2023-08-28

**Authors:** Dillon S. McBride, Sofya K. Garushyants, John Franks, Andrew F. Magee, Steven H. Overend, Devra Huey, Amanda M. Williams, Seth A. Faith, Ahmed Kandeil, Sanja Trifkovic, Lance Miller, Trushar Jeevan, Anami Patel, Jacqueline M. Nolting, Michael J. Tonkovich, J. Tyler Genders, Andrew J. Montoney, Kevin Kasnyik, Timothy J. Linder, Sarah N. Bevins, Julianna B. Lenoch, Jeffrey C. Chandler, Thomas J. DeLiberto, Eugene V. Koonin, Marc A. Suchard, Philippe Lemey, Richard J. Webby, Martha I. Nelson, Andrew S. Bowman

**Affiliations:** 1 Department of Veterinary Preventive Medicine, The Ohio State University College of Veterinary Medicine, Columbus, OH USA; 2 Division of Intramural Research, National Center for Biotechnology Information, National Library of Medicine, National Institutes of Health, Bethesda, MD USA; 3 Department of Infectious Diseases, St Jude Children’s Research Hospital, Memphis, TN USA; 4 Department of Human Genetics, David Geffen School of Medicine, University of California, Los Angeles, Los Angeles, CA USA; 5 Infectious Diseases Institute, The Ohio State University, Columbus, OH USA; 6 Center of Scientific Excellence for Influenza Viruses, National Research Centre, Giza, 12622 Egypt; 7 PathAI Diagnostics, Memphis, TN USA; 8 Ohio Department of Natural Resources, Division of Wildlife, Athens, OH USA; 9 U.S. Department of Agriculture, Animal and Plant Health Inspection Service, Wildlife Services, Columbus, OH USA; 10 Columbus and Franklin County Metro Parks, Westerville, OH USA; 11 U.S. Department of Agriculture, Animal and Plant Health Inspection Service, Wildlife Services, National Wildlife Disease Program, Fort Collins, CO USA; 12 U.S. Department of Agriculture, Animal and Plant Health Inspection Service, Wildlife Services, Wildlife Disease Diagnostic Laboratory, Fort Collins, CO USA; 13 U.S. Department of Agriculture, Animal and Plant Health Inspection Service, Wildlife Services, Fort Collins, CO USA; 14 Department of Biomathematics, David Geffen School of Medicine, University of California, Los Angeles, Los Angeles, CA USA; 15 Department of Biostatistics, Fielding School of Public Health, University of California, Los Angeles, Los Angeles, CA USA; 16 Department of Microbiology, Immunology and Transplantation, Rega Institute, KU Leuven, Leuven, Belgium

**Keywords:** Viral infection, Molecular evolution, SARS-CoV-2, Phylogenetics, Epidemiology

## Abstract

The zoonotic origin of the COVID-19 pandemic virus highlights the need to fill the vast gaps in our knowledge of SARS-CoV-2 ecology and evolution in non-human hosts. Here, we detected that SARS-CoV-2 was introduced from humans into white-tailed deer more than 30 times in Ohio, USA during November 2021-March 2022. Subsequently, deer-to-deer transmission persisted for 2–8 months, disseminating across hundreds of kilometers. Newly developed Bayesian phylogenetic methods quantified how SARS-CoV-2 evolution is not only three-times faster in white-tailed deer compared to the rate observed in humans but also driven by different mutational biases and selection pressures. The long-term effect of this accelerated evolutionary rate remains to be seen as no critical phenotypic changes were observed in our animal models using white-tailed deer origin viruses. Still, SARS-CoV-2 has transmitted in white-tailed deer populations for a relatively short duration, and the risk of future changes may have serious consequences for humans and livestock.

## Abstract (web summary)

White-tailed deer are an important reservoir of SARS-CoV-2 in the USA and continued monitoring of the virus in deer populations is needed. In this genomic epidemiology study from Ohio, the authors show that the virus has been introduced multiple times to deer from humans, and that it has evolved faster in deer.

## Introduction

As of December 2022, over 645 million human cases of severe acute respiratory syndrome coronavirus 2 (SARS-CoV-2) were reported globally, resulting in over 6.6 million deaths^1^. Evolution of this virus involves rapid mutation, recombination, and host-switching^2,3^. The emergence of new divergent variants that more efficiently transmit and/or evade host immune response has repeatedly altered the trajectory of the pandemic, delaying economic and societal recovery. Variants with unpredictable characteristics continue to evolve and the role of animal hosts in the future evolution of SARS-CoV-2 remains unclear. SARS-CoV-2 is capable of infecting a broad range of mammals^4,5^. The largest number of non-human SARS-CoV-2 infections have been detected in mink, felines, canines, and cervids^6^. Spillback of mutated variants from animals back to humans has been confirmed in hamsters in Hong Kong and mink in the Netherlands and Denmark, which led to mass culling^7–9^. However, mass culling is not feasible in free-ranging animals, such as white-tailed deer (WTD), and wildlife reservoirs present a more intractable problem.

White-tailed deer were identified as wildlife host for SARS-CoV-2 in July 2021, when antibodies against SARS-CoV-2 were detected in 40% of the tested free-ranging WTD from four US states^10^. One month later, we reported the first confirmed cases of active SARS-CoV-2 infection in free-ranging deer^11,12^. During January–March 2021, one-third of nasal swabs collected from WTD in northeast Ohio were positive for SARS-CoV-2 by rRT-PCR. Phylogenetic analysis indicated that at least six human-to-deer transmissions occurred, and one deer-to-deer transmission cluster persisted for at least several weeks. Since then, the virus has been repeatedly and frequently detected in white-tailed deer in multiple North American locations^10,11,13–16^. Retrospective testing of samples collected in Iowa identified active SARS-CoV-2 infections in WTD as early as September 2020^14^. High seroprevalence (94.4%) was reported in a captive WTD facility in Texas^17^. The omicron variant was detected in WTD in Staten Island, New York in January 2022, following the omicron wave in humans^18^. Intriguingly, an Ontario WTD clade of SARS-CoV-2 that is related to the B.1.641 lineage was identified with likely deer-to-human spillback^13^. As of January 17, 2023, The US Department of Agriculture confirmed SARS-CoV-2 by PCR in wild WTD in 27 states^6^.

Ohio is one of the most populous states in the United States (ranked 7th in population^19^) and has the 8th highest recorded number of human COVID-19 cases (2.9 million as of July 25, 2022)^20^. To further investigate the status of SARS-CoV-2 in WTD in Ohio, we conducted statewide surveillance from November 2021 to March 2022, collecting 1522 nasal swabs from WTD in 83 of Ohio’s 88 counties. This study is a large-scale effort to characterize the infection rate, persistence, spatial spread, and evolution of SARS-CoV-2 in WTD within a geographic area of approximately 115 thousand square kilometers.

## Results

### SARS-CoV-2 detected in Ohio white-tailed deer by PCR, serology, and whole-genome sequencing

**Fig. 1:**
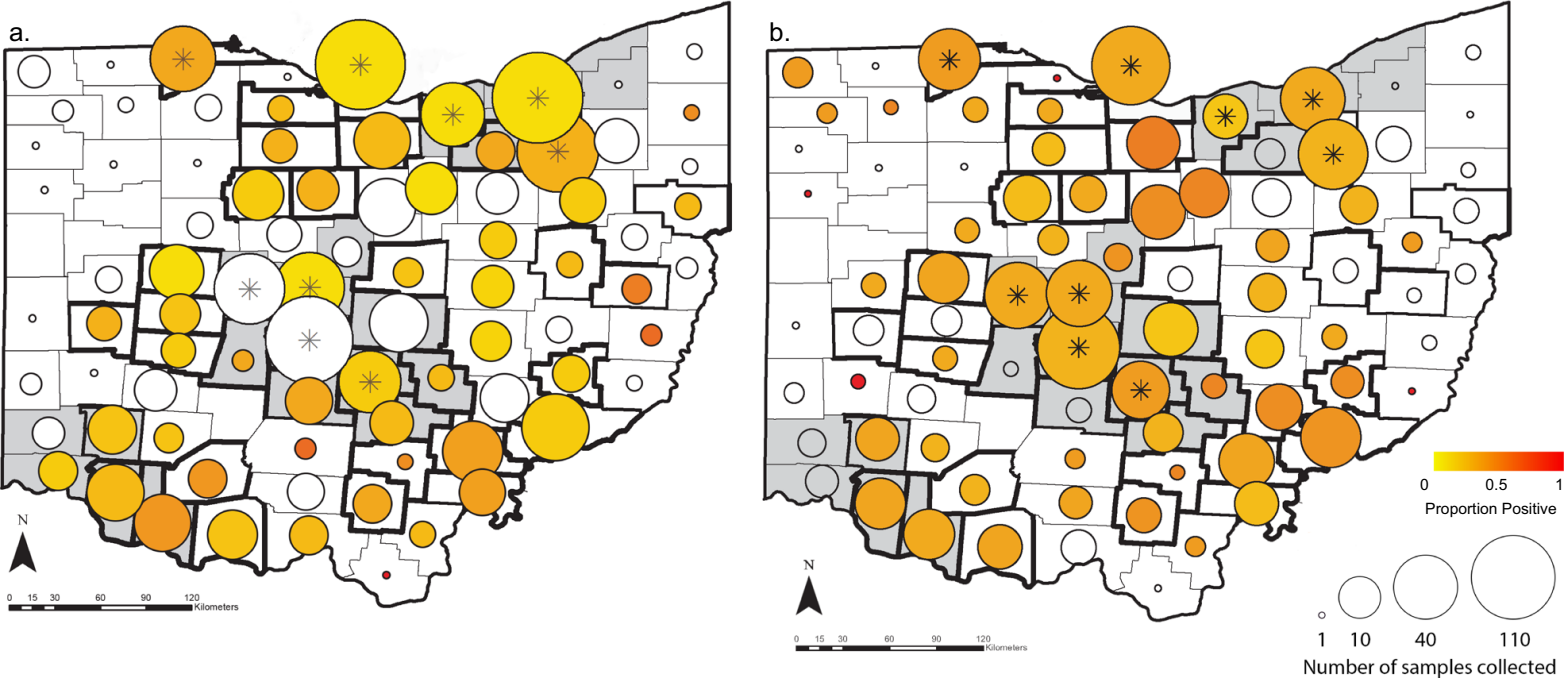
Geographic distribution of SARS-CoV-2 in Ohio by county. Counties classified as urban are colored gray and rural counties are white. The size of circles plotted over the county centroids indicate the number of samples collected and the color scale indicates SARS-CoV-2 estimated prevalence in each county by rRT-PCR (**a**) and seroprevalence by surrogate virus neutralization (**b**). Counties that are outlined in bold borders indicate counties from which we obtained SARS-CoV-2 genomic sequences (Table S2). Counties marked with an asterisk indicate counties from which samples were collected from culled WTD as a part of population management programs (Table S1). Map created with ArcMap (ESRI) using base layers and data from Esri, Garmin, OpenStreetMap, GIS user community, Infogroup and the US Census Bureau.

More than 10% of the samples collected from WTD for this study were positive by rRT-PCR for SARS-CoV-2 (163/1522, 10.7%, 95% CI 9.2–12.4%, Table S1). In more than half of the counties sampled (59.0%, 49/83), at least one SARS-CoV-2 positive WTD was identified (Fig. 1 and Table S2). Positive samples were retrieved from Ohio’s three largest metropolitan areas surrounding Cleveland, Columbus, and Cincinnati, as well as from rural areas (Fig. 1). The only region with no positive samples was the rural northwest, where sample collection was limited. There was no significant difference in the percentage of urban counties with at least one positive sample (68.4%, 13/19) compared to rural counties (56.2%, 36/64) (Chi2 0.90, *p* = 0.34). Accounting for county-level clustering, male WTD had marginally increased odds of infection compared to females (Adjusted OR = 1.5, 95% CI 1.0–2.2, *p* = 0.034, not significant at Bonferroni adjusted cut off of 0.007, Table S3), but the age of the WTD had no substantial effect. Samples collected in December had 1.8x higher odds of infection compared to November, when most samples were collected (Adjusted 95% CI 1.0–3.0, *p* = 0.035, not significant at Bonferroni adjusted cut off of 0.007, Fig. S1 and Table S3). Odds of infection were even lower in January, and no samples from February or March 2022 tested positive. We found a significant statistical interaction between the manner of death and urban county classification. Hunter-harvested WTD had similar odds of infection in urban and rural counties, but culled WTD had higher odds of infection in rural counties compared to urban counties (Adjusted OR = 14.7, 95% CI 2.2–100.0, *p* = 0.006, Table S3). Only 0.6% of WTD culled from urban counties were positive by PCR, but 22.2% of blood samples from the same WTD were seropositive for antibodies (Table S3b), indicating previous exposure in a population with very few active infections. Overall, the estimated seroprevalence of SARS-CoV-2 in Ohio WTD was 23.5% (274/1164, 95% CI 21.1–26.1%, Table S1). Seropositivity was not associated with detection of active infections by PCR (Chi2 1.7, *p* = 0.19). At least one seropositive WTD was sampled in 66.3% (55/83) of counties. Even in an urban county like Franklin (which includes Columbus), where all 149 samples from culled WTD were negative by PCR, estimated seroprevalence was 19%. Thus, urban culled WTD with few infections within our sampling time interval still had similar likelihood of previous exposure to other WTD tested for antibodies. Almost 70% (804/1164) of WTD were negative by both PCR and surrogate virus neutralization test (sVNT), indicating that a large portion of the WTD population in Ohio remained naïve to SARS-CoV-2. Fourteen counties were negative by both PCR and serology, all of which had 10 or fewer serum samples (Table S2). Given that seroprevalence was similar in male and female WTD, in urban and rural counties, and across months, we found no significant predictors for seropositivity.

**Fig. 2:**
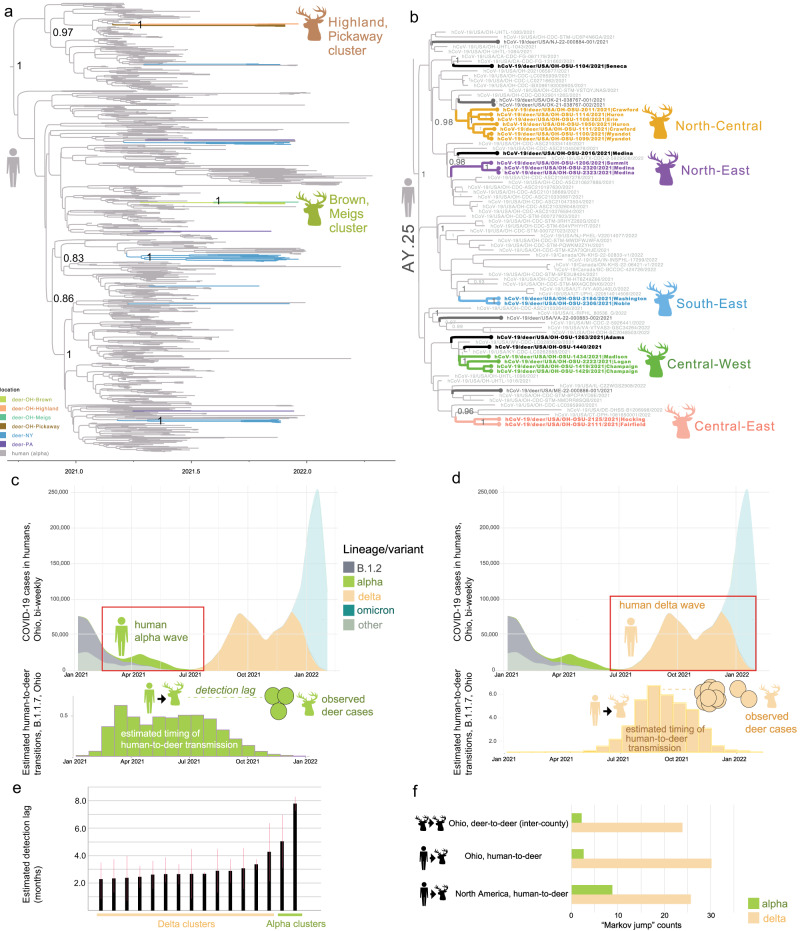
Human-to-deer transmission of SARS-CoV-2 in Ohio. **a** MCC tree inferred for 786 B.1.1.7 viruses collected from humans and WTD. Branches shaded by host species and location. The two Ohio WTD clusters are labeled. **b** AY.25 subtree (entire delta MCC tree shown in Fig. S4, *n* = 1094 delta viruses). Ohio WTD virus transmission clusters are shaded similarly to Fig. 3, with the addition of black branches indicating Ohio WTD singleton detections from this study and dark gray branches indicating non-Ohio WTD singletons. **c** The number of bi-weekly COVID-19 cases in humans in Ohio from January 2021 to February 2022, shaded by the proportion of human SARS-CoV-2 sequences from Ohio that belong to one of four Pango lineages (or ‘other’). Red box delineates the B.1.1.7 wave in humans. Below, green bars show the estimated number of human-to-deer transmission events of B.1.1.7 viruses, per 20-week increments, based on “Markov jump” counts inferred on the alpha MCC tree (*n* = 786 B.1.1.7 viruses). Green circles indicate the collection dates of 9 B.1.1.7 viruses in Ohio WTD. **d** Similar to **c**, but inferred on the delta MCC tree (*n* = 1094). **e** The detection lag (months) is the time difference between a human-to-deer transmission event (estimated) and the first observed sequence from a WTD transmission cluster, shown for 14 delta and 2 alpha WTD transmission clusters, inferred from the MCC trees. Red lines represent the two time points of the two nodes defining the branch on the phylogenetic tree on which the human-to-deer transition occurred; thick black bar represents the mean time of the branch. **f** Estimated number of human-to-deer transmission events in Ohio and North America and long-distance deer-to-deer transmission events that span Ohio counties, inferred from the MCC trees. Data for North America does not include Ohio.

Whole-genome SARS-CoV-2 sequences were obtained from 34 of the 49 counties with at least one positive WTD (69.4%), for a total of 80 sequences (Fig. 1 and Table S4). Nearly 70% of these sequences were from rural counties, corresponding closely to the distribution of PCR positive samples (74% from rural counties, Table S1). Ninety-five percent of sequenced WTD viruses were sampled when the delta variant was dominant in humans, prior to December 15, 2021 (Fig. 2 and Table S4).

### Alpha variants persist in Ohio white-tailed deer

The vast majority of the sequenced WTD viruses (88.8%, 71/80) belonged to the delta variant (B.1.617.2 and AY PANGO lineages), matching the dominant variant circulating in humans at the time (Table S4). The remaining nine WTD viruses belonged to the B.1.1.7 lineage (alpha variant) that circulated in humans in Ohio six months earlier, during the spring of 2021 (Table S4). The nine B.1.1.7 viruses were collected from four counties in southern Ohio (Brown, Highland, Meigs, Pickaway) during November and December 2021. The last human B.1.1.7 virus in Ohio was collected on August 23, 2021 (EPI_ISL_3897556). The B.1.1.7 viruses collected from Ohio WTD cluster in two separate clades on the phylogenetic tree, consistent with two independent human-to-deer transmission events (Figs. 2a and S2). The time-scaled MCC tree indicates that two human-to-deer transmission events occurred during the spring B.1.1.7 wave, an estimated 5-8 months prior to the detection of B.1.1.7 in WTD (Fig. 2a, c, e). Together, these findings suggest that two B.1.1.7 introductions from humans were followed by sustained deer-to-deer transmission in Ohio for more than five months.

### Delta variants frequently transmitted from humans to white-tailed deer

The timing of the delta wave in humans (Figs. 2d and S3) coincided with the timing of the late autumn WTD hunting season in Ohio (our most intense sampling period), and we detected frequent introductions of delta variant viruses from humans to WTD. Twelve PANGO lineages considered delta variant (eleven AY lineages and B.1.617.2) were identified in WTD, representing a minimum of 12 independent human-to-deer delta transmissions (Fig. S4). Most AY lineages were introduced into WTD from humans more than once, as evidenced by multiple independent clades in the phylogenetic tree (Figs. 2b, S2, and S4). In total, more than 30 delta reverse zoonosis events in Ohio were inferred across the branches of the MCC tree, based on “Markov jump” counts that infer the number of human-to-deer transmissions (Fig. 2e). The AY lineages detected at the highest frequency in WTD (AY.103 and AY.25) were also dominant in humans in Ohio (Figs. S5 and S6). In general, the genetic composition of delta variants in Ohio WTD matched the dominant lineages found in humans at that time, with a positive association between AY lineage frequencies in humans and in WTD (Fig. S7). The MCC tree estimates that delta variants were introduced from humans to WTD during the autumn of 2021, coinciding with the delta wave in humans in Ohio, approximately 2–3 months before their descendants were detected in WTD by surveillance (Fig. 2d, e). Frequent reverse zoonotic transmission seeded high genetic diversity in Athens and Washington counties, where four SARS-CoV-2 lineages were identified during a single week (week 49, Fig. S8). Each introduction transmitted onward in WTD enough to be picked up by surveillance, but not all introductions spread to multiple counties, and many are likely to be transient.

### Spatial diffusion of alpha and delta variants in white-tailed deer

**Fig. 3:**
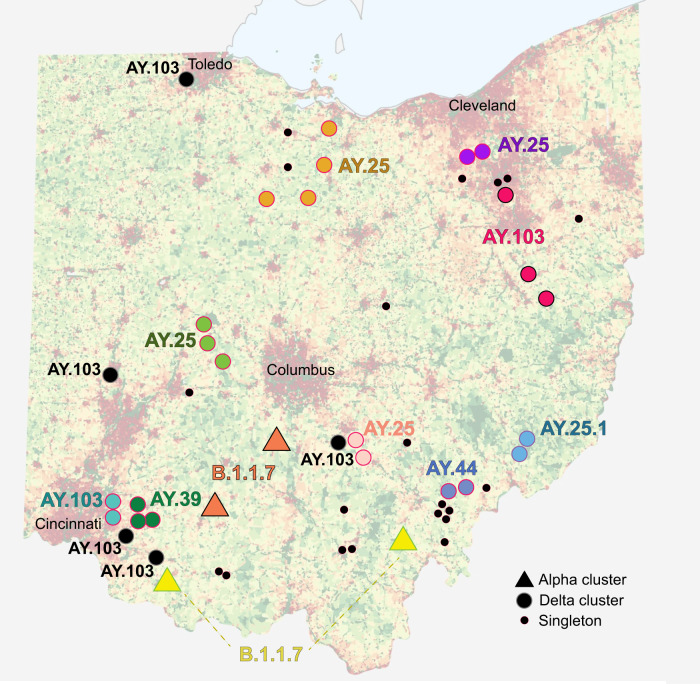
Map of SARS-CoV-2 transmission clusters in Ohio white-tailed deer. Each shape represents a county in Ohio where SARS-CoV-2 virus was identified in WTD for this study (triangle = alpha variant; circle = delta variant). Large circles indicate WTD transmission clusters, as identified on the phylogenetic tree (black = clusters restricted to one county; shaded = clusters identified in more than one county). Large circles shaded the same color belong to the same transmission cluster. Small black circles indicate singleton WTD viruses. PANGO lineage provided for all clusters. Human population density is shown in the background (red = high; green = low) and major cities are labeled. Ohio population map [created by JimIrwin, from Wikimedia Commons] was used with modification under CC BY-SA 3.0 license.

Onward deer-to-deer transmission was observed in 16 WTD clusters (14 delta and 2 alpha, Fig. 3). Eleven clusters spanned multiple counties, including four that spanned three or more counties (Figs. 3 and S9). The largest spatial clusters were found in rural areas. Delta clusters typically spanned neighboring counties that share a border, whereas alpha clusters included viruses from distant, non-contiguous counties separated by hundreds of kilometers. Longer distance dispersal of alpha variants is consistent with the longer time frame of alpha circulation in Ohio WTD inferred from the MCC tree (5–8 months, Fig. 2b, c). At this level of sampling, every virus detected in a WTD, including 20 singletons positioned outside observed transmission clusters on long branches, likely belongs to a sampled or unsampled transmission cluster in deer.

### SARS-CoV-2 evolves faster in white-tailed deer than in humans

**Fig. 4:**
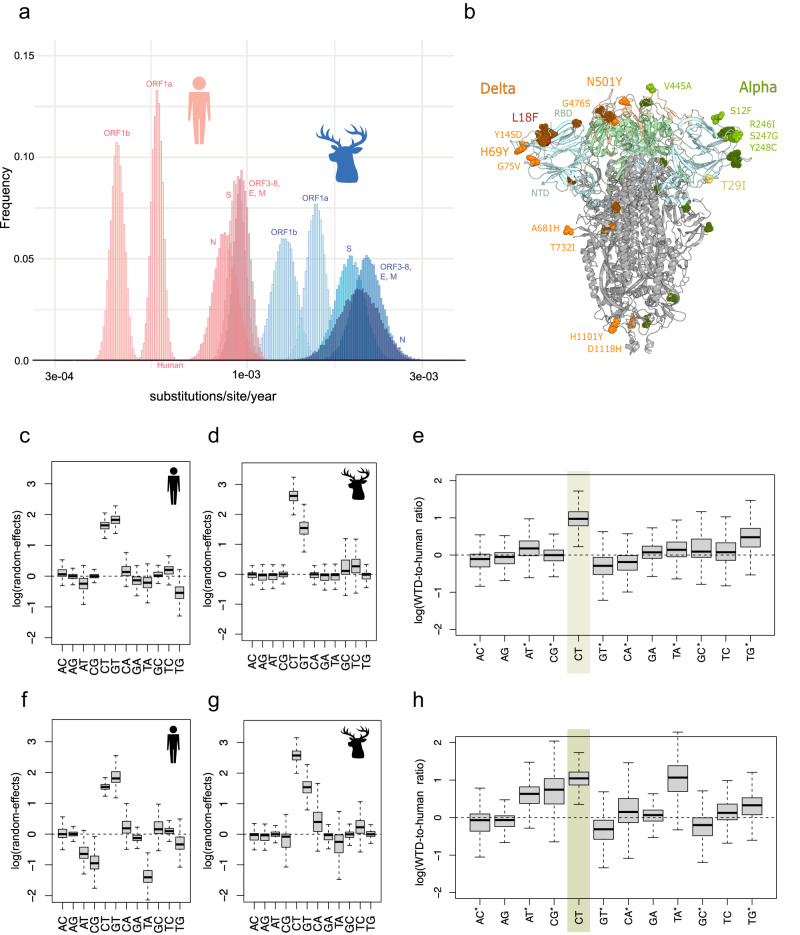
Evolutionary rate of SARS-CoV-2 in humans and white-tailed deer. **a** The posterior distributions of evolutionary rates (substitutions per site per year) for five partitions of the SARS-CoV-2 genome (ORF1a, ORF1b, ORF3–ORF8 plus envelope (E) and membrane (M), spike (S), and nucleocapsid (N)) are presented for human (pink) and WTD (blue) for the delta variant (Fig. S4). Alpha results (similar) are provided in Fig. S11. **b** Mutations in spike protein that were found in delta WTD clusters (orange), L18F is shown in red, alpha WTD clusters (green), and T29I found in both alpha and delta WTD clusters (yellow). Characteristic mutations for delta lineages (mutations present in human and WTD viruses belonging to delta compared to Wuhan reference genome) are shown in dark orange, while characteristic mutations for alpha lineages are shown in dark green. All recurrent mutations from WTD clusters are documented in Supplementary Data 2. The log deviation (random-effect) from HKY model relative rates is presented for **c** alpha, humans, **d** alpha, WTD, **e** alpha, WTD-to-human ratio, **f** delta, humans, **g** delta, WTD, and **h** delta, WTD-to-human ratio, inferred independently for alpha (*n* = 786) and delta (*n* = 1094). Box midlines indicate the median, the box limits show the upper and lower quartiles, and the whiskers extend to 1.5 times the interquartile range. Asterisks indicate transversions. WTD-to-human ratios that significantly differ from zero are highlighted.

To explore whether WTD viruses’ long branches (Fig. S2) and deviation from genetic distance root-to-tip regressions (Fig. S10) arise because SARS-CoV-2 was evolving faster in WTD than in humans, we performed an in-depth analysis of all mutation events, scanned for positive and negative selection across the SARS-CoV-2 genome, and estimated rates of nucleotide substitution using a Bayesian approach. The estimated rate of SARS-CoV-2 evolution (substitutions per site per year) was approximately three times higher in WTD compared to humans for the alpha variant and 2.7 times higher for the delta variant (Figs. 4a and S11, Table S5). The rate was higher in WTD for both synonymous and non-synonymous substitutions across all genome regions, including the spike protein (Fig. S12). However, the rate of non-synonymous substitution in WTD was not as high as would be expected, given the background rate of synonymous substitution. As a result, the ratio of non-synonymous to synonymous substitutions (dN/dS) was lower in WTD than in humans, across the genome, for both alpha and delta variants (Fig. S12). The dN/dS ratio for delta spike protein was significantly lower in WTD compared to humans (Fig. S12), evidence of predominantly purifying selection that removes non-synonymous changes (Table S6). A high rate of non-synonymous substitution was observed in WTD for the genome partition that includes ORFs 3-8, the envelope, and membrane proteins. ORF3a had a high dN/dS ratio in WTD in an analysis of selected genes (See “Methods”, Table S6).

The high rate of SARS-CoV-2 evolution in white-tailed deer observed in our study could be a feature of a high rate of transmission in an immunologically naïve host with weakened purifying selection, similar to the early phase of SARS-CoV-2 evolution in humans. To test this hypothesis, we conducted an additional comparison with early SARS-CoV-2 strains collected in humans during December 2019 to February 2020 that were used in Pekar et al.^21^. The overall rate of SARS-CoV-2 evolution was significantly higher in early human strains (1.3 × 10^−3^ substitutions/site/year; 95% HPD 1.1–1.6 × 10^−3^) compared to the alpha and delta strains that emerged in humans later in the pandemic (5.9–6.0 × 10^−4^), but not as high as the deer rate (1.6–1.8 × 10^−3^) (Table S5 and Fig. S13). However, dN/dS was higher in the spike protein gene of human variants, especially for delta, compared to deer and early human, consistent with increasing immune-driven positive selection as a population becomes exposed (Fig. S12).

### Mutational biases in white-tailed deer

To investigate the possible causes of faster SARS-CoV-2 evolution in WTD compared to humans, we examined differences in mutational bias between human and deer branches in the phylogenetic tree. WTD had a significantly higher C-to-T mutational bias for both alpha and delta variants (Fig. 4c–h, Table S7, Supplementary Data 1). This pattern held for all mutations, including synonymous mutations, and could not be attributed to selection, low WTD sample size, or differences in codon usage between WTD and humans (Figs. S14–S17). Most C-to-T mutations occurred in the WCW context (where W can be A or T), which has been linked to APOBEC activity in humans^22–24^ and would be consistent with previous experimental evidence that suggested APOBEC editing as an important contributor of mutations in human origin SARS-CoV-2 genomes^25,26^. The higher C-to-T bias in deer was maintained, even compared to early SARS-CoV-2 strains from humans (Fig. S18) and there was no evidence that C-to-T biases changed over time in humans.

### Recurrent mutations in spike protein in WTD

Recurrent mutations arose independently in different WTD transmission clusters that could potentially represent individual sites under positive selection (Figs. S19 and S20). For example, the L18F mutation in the spike protein N-terminal domain occurred independently in six WTD delta clusters and two singletons (Fig. 4b; Supplementary Data 2; Fig. S19). This site was estimated to be subject to episodic diversifying positive selection (p-value 0.0001). The S:L18F mutation occurred during the Hong Kong hamster outbreak^7^ and reduced antibody binding in the gamma variant in humans^27^. However, the mutation dropped to low frequencies (<1%) globally in human delta and omicron (Fig. S21). Seven additional recurrent WTD mutations were located on the spike protein surface, including N501Y in the receptor binding domain (RBD) (occurred twice) and H69Y. S:N501Y was a key mutation^28^ in human variants (alpha, beta, gamma, omicron, mu), but was rarely observed in delta viruses in humans. No recurrent spike mutations were found in the smaller alpha dataset, but S:T29I was observed in both alpha and delta.

### In vitro growth kinetics

Virus isolation was attempted on 75 of the 163 PCR positive nasal swabs, yielding 27 SARS-CoV-2 isolates for further laboratory characterization. We analyzed the replication kinetics of nine representative WTD isolates (Table S8) compared with the corresponding ancestral strains from humans, Hu-B.1.1.7 and Hu-B.1.617.2. The AY.75 virus displayed significantly lower titers at 24- and 36-h post infection (hpi) compared to the other isolates in Vero E6 T2 cells (Fig. S22A). No significant differences were observed between the WTD and human B.1.1.7-like viruses in any cells (Fig. S22A–C). Interestingly, some of the deer variants demonstrated reduced replication efficiency in Calu-3 cells (B.1.1.7-like, AY.109, AY.39 and AY.75), whereas the remaining viruses grew efficiently in Calu-3 cells albeit to lesser titers than the Hu-B.1.617.2 virus (Fig. S22C).

### Antigenic characterization

**Fig. 5:**
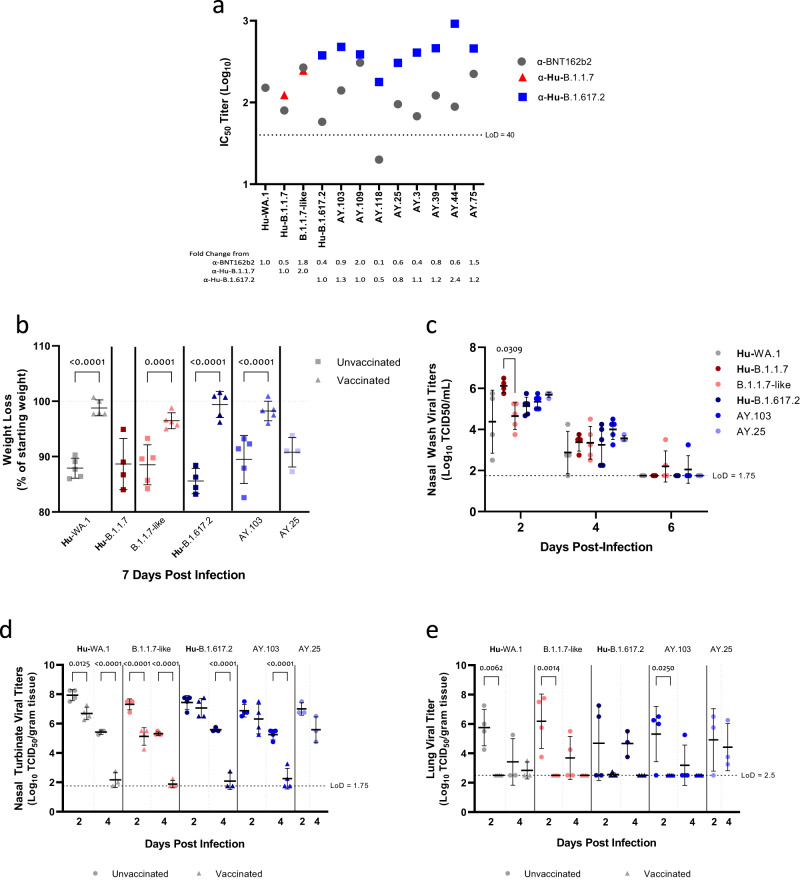
Pathogenicity and replication of multiple strains of SARS-CoV-2 viruses in Golden Syrian hamsters. Golden Syrian hamsters were challenged with Hu-WA.1 (unvaccinated *n* = 12, vaccinated *n* = 12), Hu-B.1.1.7 (unvaccinated *n* = 4), B.1.1.7-like (unvaccinated *n* = 13, vaccinated *n* = 13), Hu-B.1.617.2 (unvaccinated *n* = 11, vaccinated *n* = 12), AY.103 (unvaccinated *n* = 13, vaccinated *n* = 13), and AY.25 (unvaccinated *n* = 10). All sample sizes reflect biologically independent animals. **a** Microneutralization titers of a-BNT162b2 or lineage specific serum against representative viruses from this study. For **b–e**, the mean for each group is plotted, and bars indicate standard deviation. Titers expressed as log_10_ IC_50_ were plotted and described as a fold change from the reference strains. **b** Body weight loss comparison between unvaccinated and BNT162b2 vaccinated animals at the peak of infection, day 7. Mean weights are displayed as a percentage of starting weight. Nasal wash was collected (unvaccinated groups only) (**c**) or lung and nasal turbinate were harvested (**d**, **e**) and used to quantify viral titers. Viral titers expressed as the log_10_ TCID_50_ were plotted. Statistical analysis was performed using one-way ANOVA followed by a Tukey post hoc, *p* values displayed from Tukey’s test statistic, q, which controls for family wise error rate for multiple comparison.

The neutralizing capacity of α-Hu-B.1.617.2 hamster serum was equivalent or greater against all the Delta lineage viruses isolated from WTD compared to the parental Hu-B.1.617.2 virus, except for AY.118 and AY.25 which had a 2.1 and 1.2-fold-reduction in neutralization, respectively (Fig. 5a). The α-BNT162b2 serum had detectable neutralizing titers against all Alpha and Delta lineage viruses tested except for the AY.118 isolate. Overall, some exceptions notwithstanding, most WTD viruses were antigenically similar to their human counterparts.

### In vivo experiments

A subset of WTD viruses was selected for analysis of viral replication and pathogenesis in Syrian Golden hamsters, an established animal model for SARS-CoV-2 variants^29–31^. Throughout the infection in unvaccinated animals, there was no substantial difference in the weight loss observed between the animals infected with different viruses (Fig. 5b). In each case, prior immunization with BNT162b2 led to decreased weight loss, suggesting that current vaccines would be expected to provide protection from severe disease if these viruses were to spill back into the human population (Fig. 5b). No differences were observed between the Hu-B.1.617.2 and the AY.3 or AY.25 nasal washes from unvaccinated hamsters. The Hu-B.1.1.7 virus showed a significantly higher titer at 2 dpi compared to the B.1.1.7-like strain, but that difference was not observed for the later time points (Fig. 5c). Vaccination resulted in a reduction in viral titers compared to unvaccinated hamsters for the majority of strains at 2 dpi and was most pronounced at 4 dpi (Fig. 5e, d).

## Discussion

Three years into the COVID-19 pandemic, gaps in our knowledge of the virus’s broader ecology and evolution in non-human hosts impede efforts to fully resolve the pandemic’s zoonotic origins and predict its future evolutionary trajectory. Here, by capturing the early phase of SARS-CoV-2 transmission in WTD, we observe how a flexible generalist virus invaded a new host species without extensive adaptive evolution or phenotypic change. The persistence of WTD transmission clusters for 2–8 months provided data with enough resolution to robustly estimate that SARS-CoV-2 evolves about three times faster in WTD than in humans SARS-CoV-2 evolution in WTD using Bayesian approaches. The high rate of SARS-CoV-2 evolution, apparent lack of extensive adaptive evolution, and absence of phenotypic change all suggest that the virus is evolving under purifying selection and transmitting efficiently in WTD without (thus far) necessitating major evolutionary changes.

Other zoonotic viruses, such as influenza A virus, evolve at different rates in humans and other mammalian species. Higher overall rates of influenza A virus evolution in non-human hosts (e.g., swine and birds)^32^ raise questions about how virus evolution is modulated by population turnover, host metabolism, the activity of RNA editing enzymes, in particular APOBEC, and other physiological and ecological factors. Influenza serves as a reminder of how pathogens that jump from humans to animals can establish reservoirs with different evolutionary trajectories that can ultimately have consequential effects on animal and human health^33^. While new, fitter influenza variants continuously replace and purge older diversity in humans, pigs sustain older human strains that shuffle their genomes through reassortment and periodically spill over into humans, turkeys, and canines, causing localized outbreaks or global pandemics. It remains questionable whether the ecology and contact rates of free-ranging WTD can sustain an acute respiratory pathogen like SARS-CoV-2 over the long term without involving other host species or environmental persistence. The enigma of how SARS-CoV-2 transmits so frequently from humans to WTD reflects broad gaps in our understanding of SARS-CoV-2 ecology.

Our study demonstrates that SARS-CoV-2 viruses sustained transmission in free-ranging WTD for up to eight months in Ohio in 2021. First, humans seeded dozens of SARS-CoV-2 outbreaks in WTD, scattered across the state. Then, WTD movements spread some viruses regionally over hundreds of kilometers, both in rural areas and on the outskirts of metropolitan areas. The persistence and clustering of alpha and delta lineage SARS-CoV-2 we detected in Ohio WTD are consistent with recent results from New York^16^. To understand how SARS-CoV-2 transmits deer-to-deer between subpopulations, future efforts should integrate WTD movement data collected over many years by wildlife biologists tracking pathogens of concern for livestock, such as chronic wasting disease and tuberculosis^34,35^. Our findings are broadly consistent with patterns observed in other WTD diseases, including higher infection odds in males that travel and contact other WTD during breeding season^36^. Higher infection odds in December could be a bump after peak mating season, which increases WTD contact rates and movements through early November. Rising delta cases in humans could also be a factor. The timing of our sampling coinciding with the fall hunting season seemed to play a large role in where we detected active outbreaks. The very low odds of detecting active infection in deer culled from urban counties, in contrast to the lack of a similar effect in serological samples, points strongly to the fact that our sampling efforts were not able to catch the window of active infection in the majority of the culled urban reservation deer populations we sampled. Deer in urban reservations where hunting is not permitted might interact with humans differently, driving differences in outbreak timing. All these hypotheses require quantitative testing with mathematical models built from empirical movement data and finer resolution, year-round virological surveillance.

The sheer frequency of human-to-deer spillover observed in Ohio—and North America—is consistent with a growing consensus that cross-species transmission of SARS-CoV-2 occurs far more frequently than previously detected. The yet-to-be-determined interface for human-to-deer transmission events appears to exist across the entirety of Ohio, regardless of proximity to major metropolitan areas. We cannot exclude the possibility that all detected introductions are due to direct human contact, although it seems unlikely. Indirect environmental transmission through wastewater and stormwater^37,38^ is possible, but the virus has never been successfully isolated from wastewater, let alone run-off. The scope of airborne transmission of SARS-CoV-2 in indoor and outdoor environments has been a source of lively discussion since the onset of the pandemic. Experimental studies of SARS-CoV-2 transmission in WTD and other wildlife are needed but conducting such experiments in BSL-3 adds cost and logistical hurdles. Other wildlife or domestic species susceptible to SARS-CoV-2 infection may have contact with and facilitate transmission to WTD. Recent work in Virginia, USA, has identified high seroprevalence in species with shared human habitats including raccoons, squirrels, skunks, and white-footed and deer mice, in addition to viral shedding in a opossum^39^. These species could plausibly interface with humans and WTD. However, surveillance in wildlife is limited, which makes identification of other potential intermediate hosts difficult.

There are approximately 30 million WTD in the United States, and that population is increasingly urbanized and in close contact with humans^40^. Although there is evidence of transmission of WTD origin SARS-CoV-2 to humans^13^, no substantial outbreaks of deer-origin SARS-CoV-2 in humans have been reported. Our experimental results indicate that, in general, the WTD lineage viruses have not undergone substantial enough antigenic change to be a risk for immune and vaccine escape in human populations. Going forward, we predict that most delta transmission clusters seeded in WTD will die off and fail to persist long-term, a common phenomenon in SARS-CoV-2 population genetics that has been documented in humans in intensively sequenced locations^41^. However, as we observed for alpha, a subset might persist in WTD. It is possible that the overall rate of SARS-CoV-2 evolution in deer could decline over time, as occurred in humans, and that selection on specific epitopes involved in immune escape could strengthen as deer become more exposed and produce antibodies. Additional surveillance data will also reveal how successfully omicron variants invaded North American WTD and whether co-circulation of delta, alpha, and omicron provided breeding grounds for new recombinants. SARS-CoV-2 is currently not considered an important risk for North American livestock, but the continued spread of the virus in WTD, humans, and other hosts could open new pathways for SARS-CoV-2 evolution.

## Methods

### Sample collection

Previously, we collected nasal samples from WTD in northeastern Ohio focusing on metropolitan area parks^11^. In the present study, we expanded geographical study area by approximately 1000-fold to target the entire state of Ohio. We collected 1522 nasal swabs from WTD across 83 of Ohio’s 88 counties from October 2021 to March 2022. Of those, 713 samples were opportunistically collected from hunter-harvested WTD during hunting season (November to December 2021) in 81 counties in Ohio, drawing primarily from rural areas. In addition to hunted WTD, we also collected 801 samples from WTD culled during deer population management programs from 9 counties in Ohio. We had 2 additional samples collected from roadkill WTD, and 6 nasal swabs that did not have sufficient metadata to identify the manner of death. We had a large sample size discrepancy between adult (*n* = 1199) and juvenile WTD (*n* = 297), which contributed to the reduction of power and may bias the estimates. This large difference in sample between ages is likely due to hunters’ common preference to take larger deer.

Members of the public are permitted to hunt WTD in Ohio during an explicit time frame each autumn (following WTD mating season). Hunter-harvested WTD samples were collected at locations where other cervid disease surveillance programs were being conducted concurrently. Samples from culled WTD were collected in partnership with each respective population management program at their individual deer processing sites. Culled WTD are typically baited at their respective reservations and managed land sites (where hunting by the general public is not permitted). Professional sharpshooters then harvest WTD from those sites to reduce WTD population in accordance with the deer management plan for that location. Nasal swabs were collected from free-ranging WTD during the six-month period from October 2021 to March 2022. Most samples were collected during November-December 2021, corresponding to the gun hunting season in Ohio (Fig. S1). Sample collectors from all partner organizations were trained on standard sample collection methods. Collectors wore a facemask and changed gloves between all samples. Two sterile polyester tipped swabs were used during nasal swab collection. The first swab cleared any debris from the exterior of the nostrils. A second sterile swab was inserted fully, scraped epithelial cells and nasal fluids of both nasal passageways and placed into a tube containing 3 mL viral transport media (BD UVT cat #220220). Post-collection, nasal swab samples were chilled temporarily in the field until transport to our laboratory at The Ohio State University and stored at −80 °C until diagnostic testing. We also collected blood samples to test for antibodies against SARS-CoV-2 in collaboration with USDA. We collected blood samples from 1164 of the 1522 WTD from which we collected nasal swabs. Blood was collected using 2 high purity cellulose fiber filter paper (Nobuto strips) dipped into pooled blood from WTD carcasses to saturate the paper with approximately 0.1 mL of blood. Nobuto strips were labeled and dried prior to transport. Any carcasses that did not appear fresh, had already been substantially processed, exterior blood appeared heavily contaminated, or did not have visible blood sources available for collection were excluded. Due to post-mortem sample collection, the study was exempt from a scientific permit from the Ohio Department of Natural Resources and beyond the scope of The Ohio State University Institutional Animal Care and Use Committee.

### Diagnostic testing

Viral RNA extraction was conducted using the Omega Bio-tek Mag-Bind Viral DNA/RNA 96 kit (cat# M6246·03), with 200 µl of sample^11^. Extracted viral RNA from samples was tested via real-time reverse transcription polymerase chain reaction (rRT-PCR) using the E gene primer/probe panel (Integrated DNA Technologies, Inc. cat #1006804) with Xeno VIC Internal Control Assay (Life Technologies cat #A29765)^11^. Any sample with a cycle threshold (Ct) value of 40 or below were considered E assay positive. All samples that screened E assay positive were confirmed using the Charité/Berlin RdRp confirmatory assay^42^ (Integrated DNA Technologies, Inc. cat #10006805) or the CDC N1/N2 kit (Integrated DNA Technologies, Inc. cat #10006606) diagnostic procedure. Samples with a Ct of ≤40 on either confirmatory assay were classified as positive for SARS-COV-2.

Blood samples were tested for evidence of prior exposure to SARS-CoV-2 as previously described with minor modifications^10^. Briefly, antibody elution from Nobuto strips was accomplished by incubating each strip in 1 mL BupH Tris-buffered saline (pH 7.2) (TBS, Thermo Fisher) containing 3% nonfat dried milk (Sigma) and 0.1% Tween 20 (Sigma) (TBSNT). Following incubation, samples were mixed by vortexing and debris then removed by centrifugation at 5000 × *g* for 10 min at ambient temperature. Supernatants were transferred to sterile microcentrifuge tubes and stored at −80 °C until use. The effective dilution of antibody in each eluate was estimated at 1:20. Sixty microliters of each eluted sample was directly (without further dilution) analyzed using the GenScript SARS-COV-2 Surrogate Virus Neutralization Test (sVNT, L00847-A) in accordance with the manufacturer’s instructions^43^. GenScript’s sVNT has a manufacturer’s provided specificity of 99.40% in humans, but no specificity analysis has been done in WTD. All samples were tested at least twice, with the average % inhibition of technical replicates used for the qualitative interpretation of SARS-COV-2 exposure.

### Genomic sequencing

The Ohio State Applied Microbiology Services Laboratory attempted genomic sequencing on all positive samples with a Ct value of 33 or lower (*n* = 86 samples). RNA was reverse transcribed into cDNA and PCR amplified using the ARTIC v 4.1 SARS-CoV-2 primer panel (Integrated DNA Technologies, Inc. cat #10011442) and NEBNext® FS Library Prep Kit for Illumina® (New England Biolabs, Ipswich MA) per manufacturers protocol instructions. Illumina sequencing libraries were prepared using RNA Prep with Enrichment (L) Tagmentation Kit (Illumina, San Diego, CA) per manufacturers protocol with unique dual indexes (Illumina). Libraries were pooled and quantified using ProNex NGS Library Quant Kit (NG1201, Promega Co. Madison, WI). Sequencing with NextSeq 2000 (Illumina) and assembly by DRAGEN COVID Lineage app v.3.5.6 (Illumina) were performed as previously described^11^.

### Data visualization

Maps were generated using ArcMap (ESRI). For statistical analysis, location was coded using USDA Rural Urban Continuum Codes^44^. Codes range on a scale of 1–9 with increasing numbers corresponding to decreasing population size. For analysis purposes, an RUCC of 1 was considered urban – corresponding to counties surrounding Ohio’s three major metropolitan areas of Cincinnati, Cleveland, and Columbus. All other RUCC values (two through nine) were considered rural for analysis, but smaller metropolitan areas that fall into this category are indicated to aid in understanding spatial clustering. To evaluate risk factors associated with detection of SARS-CoV-2, we fit a mixed-effects logistic regression model (STATA 14.2, StataCorp LLC). We included random intercepts for county of sample collection to account for spatial clustering of sample collection. A highly significant (*p* value < 0.00005) likelihood ratio test compared to a standard logistic model supported that the mixed-effects model was better fit for our data. To evaluate the potential for unobserved heterogeneity at the county level, we fit a correlated random effects model incorporating cluster specific means^45^ and did not find significant evidence for differences in the individual level effects within clusters compared to their effects between clusters. Fixed effects were estimated for urban vs rural binary classification for the county, culled vs hunted WTD, WTD sex, WTD age, and a categorical effect for the month of sample collection to evaluate any changes over the course of the season (Table S3).

### Phylogenetic analysis

First, to determine how viruses obtained from WTD in Ohio (*n* = 80) were genetically related to SARS-CoV-2 viruses circulating in humans in Ohio and mink and WTD in other North American locations, a background dataset of complete genome sequences was compiled from GISAID (downloaded May 24, 2022). A total of 44,456 human SARS-CoV-2 viruses, collected in Ohio during February 2, 2020 – May 10, 2022, were downloaded from GISAID. This study benefited from the availability of a large number of SARS-CoV-2 sequences generated by the Ohio Department of Health, the US Centers for Disease Control, and other laboratories. County-level data was provided by the Ohio State Department of Health, which confirmed that the dataset was spatially representative, with the number of sequences available from an Ohio county proportional to the population size of the county (Fig. S23). Pangolin^46^ was used to assign a lineage to each human virus and ten viruses from each Pango lineage were randomly selected for the final background dataset using a customized Python script (*n* = 1592 human viruses). Outbreaks of genetically similar viruses from mink in Canada or a US state (Michigan, Oregon, Utah, and Wisconsin) were downsampled to 5 viruses per day, resulting in a total of 140 North American mink viruses in the final dataset. In addition, 145 viruses from WTD were included from Canada and 14 US states (Arkansas, Illinois, Iowa, Kansas, Maine, Massachusetts, Minnesota, New Jersey, New York, North Carolina, Oklahoma, Pennsylvania, Tennessee, and Virginia). The dataset was aligned using NextClade with Wuhan-Hu-1 as a reference. In-house python scripts were used to remove non-coding regions and mask sites that are known to be unreliable. A phylogenetic tree was inferred from this dataset using maximum-likelihood methods available in IQ-TREE 2^47^ with a GTR + G model of nucleotide substitution and 1000 bootstrap replicates, using the high-performance computational capabilities of the Biowulf Linux cluster at the National Institutes of Health (http://biowulf.nih.gov). The inferred tree was visualized in FigTree v.1.4.4. White-tailed deer transmission clusters were defined by monophyletic groups of WTD viruses supported by high bootstrap values (>70) and confirmed or refined using UShER^48^ (Ultrafast Sample Placement on Existing tRees). One large cluster of 8 deer viruses in Miami, Columbiana, Harrison, Summit, and Tuscarawas counties turned out to be 3 independent human-to-deer transmission events (2 transmission clusters – one in northeast Ohio and one in western Ohio—and one singleton) upon further inspection in UShER, which created sub-trees with the 50 most closely related sequences out of a dataset of more than 12 million SARS-CoV-2 genome sequences from GISAID, GenBank, COG-UK, and CNCB. This exercise illustrated the importance of not overinterpreting apparent transmission clusters based on sequence similarity alone and the need to account for missing data in phylogenies.

### Bayesian analysis

Bayesian approaches were used to examine the evolutionary relationships between alpha variants and delta variants in humans and WTD in greater detail and compare their evolutionary rates. Separate datasets were generated for alpha (B.1.1.7) and delta (B.1.617.2 and AY lineages). In addition to the alpha and delta sequences obtained for the ML tree, above, sequences from more recently sampled alpha and delta viruses in humans globally were added to provide a longer period of data. The final alpha dataset (*n* = 786 sequences) included 9 viruses from WTD in Ohio collected for this study (November 8, 2021 – December 4, 2021); 31 viruses from WTD in Pennsylvania and New York (October 2, 2021 to December 4, 2021); 677 viruses from humans in Ohio (December 29, 2020 to August 23, 2021); and 69 additional human viruses sampled globally from November 1, 2021 to March 31, 2022. The final delta dataset (*n* = 1094 sequences) included 67 viruses from WTD in Ohio collected for this study (November 6, 2021 to January 20, 2022); 36 viruses from WTD in other North American locations (October 28, 2021 to January 30, 2022); 642 viruses from humans in Ohio (April 26, 2021 to April 18, 2022); 319 additional human viruses sampled globally from March 1, 2022 to July 19, 2022; plus 30 additional human viruses sampled from the United States during peak delta activity (July 10, 2021 to November 24, 2021) that helped resolve portions of the tree where human and WTD viruses were closely related.

We performed a time-scaled Bayesian analysis using the Markov chain Monte Carlo (MCMC) method available using the pre-release 1.10.5 version of the BEAST^49^ package available on GitHub (compiled on October 20, 2022), using GPUs available from the NIH Biowulf Linux cluster. A host-specific local clock^32^ was used to accommodate differences in the evolutionary rate between WTD and humans. Since WTD viruses were not monophyletic on the alpha or delta tree, owing to multiple independent human-to-deer transmission events, separate WTD transmission clusters identified on the ML tree were specified. The analysis was performed two ways, excluding WTD singleton viruses that are not positioned in a WTD transmission cluster and including WTD singleton viruses. A Bayesian non-parametric demographic model^50^ was used, with a general-time reversible (GTR) model of nucleotide substitution with gamma-distributed rate variation among sites. The MCMC chain was run separately 3–5 times for each dataset using the BEAGLE 3^51^ library to improve computational performance, until all parameters reached convergence, as assessed visually using Tracer v.1.7.2. At least 10% of the chain was removed as burn-in, and runs for the same dataset were combined using LogCombiner v1.10.4. A MCC tree was summarized using TreeAnnotator v.1.10.4. To compare evolutionary rates across different regions of the SARS-CoV-2 genome, the analysis was repeated using five genome partitions: ORF1a, ORF1b, ORF3–ORF8, spike (S), and nucleoprotein (N). Several additional analyses were performed, including a phylogeographic discrete trait analysis^52^ to quantify rates of viral gene flow, particularly in the directions of human-to-deer and long-distance (across Ohio county lines) deer-to-deer transmission. A location state was specified for each viral sequence. All human viruses were categorized as “human,” whereas WTD viruses were also categorized by location of collection, with state information used for WTD viruses collected outside of Ohio and county information provided for all Ohio WTD viruses collected for this study. The expected number of location state transitions in the ancestral history conditional on the data observed at the tree tips was estimated using ‘Markov jump’ counts^53,54^, which provided a quantitative measure of asymmetry in gene flow between defined populations. To estimate absolute rates of synonymous and non-synonymous substitutions as well as dN/dS, we employ a ‘renaissance counting’ procedure that combines Markov jump counting with empirical Bayes modelling^55^. The outputs of these analyses (Markov jump counts, evolutionary rate distributions) were summarized and visualized using customized R scripts. Finally, we performed a flexible random effects analysis of the evolutionary substitution process^21^ to capture mutational bias differences along the human and WTD branches of evolutionary history. Mutational bias was measured by deviations from the Hasegawa-Kishino-Yano (HKY) substitution model that accommodates unequal base frequencies and different rates of transition and transversion substitutions. To facilitate efficient sampling of the additional random effects parameters, this analysis took advantage of gradient-based Hamiltonian Monte Carlo for phylogenetics within BEAST^56^. As a sensitivity analysis, we repeated our analysis to compare the early evolution of SARS-CoV-2 in white-tailed deer following a host-switch against the early evolution of SARS-CoV-2 in humans during December 2019 to February 2020, using the same dataset that was analyzed in Pekar et al. ^21^, (787 genomes, predominantly from China) except no rate prior was used.

### Epidemiological data

The epidemiological curve of SARS-CoV-2 cases in humans in Ohio from January 1, 2021 to January 22, 2022 was generated using the number of daily reported COVID-19 cases in the state of Ohio (all age groups), available from the US Centers for Disease Control and Prevention (https://data.cdc.gov/Case-Surveillance/COVID-19-Case-Surveillance-Public-Use-Data-with-Ge/n8mc-b4w4). To estimate the proportion of COVID-19 cases belonging to different Pango lineages during each week of the epidemic, SARS-CoV-2 sequences collected from humans in Ohio during this time period were downloaded from GISAID. To account for inconsistencies in the intensity of viral surveillance, the number of viruses per lineage per week was normalized against the epidemiological curve derived from COVID-19 case counts and visualized using R. To further minimize biases only sequences categorized in the GISAID submission as obtained using a ‘baseline surveillance’ sampling strategy were included in the analysis. The dataset was further trimmed to include only submissions with complete collection dates and sufficient coverage to assign a Pango lineage, resulting in a final dataset of 27,187 sequences from Ohio. For simplicity, sub-lineages of B.1.617.2 (for example, AY.3) were consolidated into the Delta category, sub-lineages of B.1.1.7 (for example, Q.3) were consolidated into the Alpha category, and sub-lineages of B.1.1.529 (e.g., BA.1) were consolidated into the Omicron category.

### Mutation analysis

We used root-to-tip regression to visualize the rate of substitution over time for the omicron variant, deer viruses, and all other SARS-CoV-2 lineages. To correctly assign mutation status during annotation to alignment in the phylogenetic analysis section, reference Wuhan genome (NC_045512.2) was added with MAFFT version 7.475^57^; the Wuhan genome was then removed to preserve the original set of sequences, while allowing for the correct alignment length representing all positions in the SARS-CoV-2 genome. This alignment together with the corresponding phylogenetic tree (see Phylogenetic analysis section) were used to reconstruct states at all tree nodes with TreeTime^58^ ancestral v 0.9.0-b.2 using default parameters. Mutations were extracted from the tree and reformatted into vcf format. Obtained vcf files were annotated with SnpEff^59^ v 4.5, and NC_045512.2 was utilized as a reference. Because mutations that happen along the tree do not always have the same nucleotide in REF field as position in genome, we corrected the annotation for the cases when those two did not match. The final annotated vcf and phylogenetic tree were used to count the number of mutations occurring from root to each leaf. We considered all mutations, synonymous and missense independently. The obtained results were visualized in R. To calculate the linear regression slope we excluded all WTD samples and human omicron data.

To accurately analyze mutations accumulating in WTD on delta and alpha backgrounds, datasets described in the Bayesian analysis section were utilized. First, we added reference to each alignment as described above, and then reconstructed phylogenetic trees with IQTree using the following parameters: --polytomy -m GTR + G --alrt 1000. Root in both cases was placed at the reference genome. States at nodes were reconstructed as described above. VCF files were produced independently for WTD clusters and WTD singletons. In alpha dataset we utilized not only the two clusters from Ohio, but all available alpha clusters (7 in total, Fig. S20). VCF annotation was produced as described above. Mutations in known problematic sites were filtered out using a list available at https://github.com/W-L/ProblematicSites_SARS-CoV2. Individual transmission clusters were visualized with ete3 python package^60^. Observed mutations on the spike trimer were visualized using the Protein Data Bank (PDB; rcsb.org), structure ID 7JJI. Structure visualization was performed with Open-Source PyMOL version 2.4.0 (Schrödinger, LLC). R-package MutationalPatterns^61^ was utilized to reconstruct mutational contexts. For input, we utilized mutations in WTD clusters and data on mutations in humans, the latter of which was extracted from the public version of the UShER (Ultrafast Sample placement on Existing tRee) tree downloaded on 2022-07-01 (http://hgdownload.soe.ucsc.edu/goldenPath/wuhCor1/UShER_SARS-CoV-2/2022/07/01/public-2022-07-01.all.masked.pb.gz), containing 5.7 million sequences. Variability in mutational contexts in humans was estimated by producing a series of subsamples containing the same number of mutations as observed in WTD delta clusters. The 10 subsamples were utilized to perform a permutation test to estimate significance of elevated C > T rate in WTD.

### Selection analysis

The HyPhy v.2.5.40 package was used to study positive and negative selection^62^. For this analysis we selected the four genes with highest number of homoplastic sites in clusters (N, S, ORF3a and nsp3). Samples that contained missing data (Ns) in the studied gene were removed, because HyPhy is unable to perform calculations on the missing data. Three different methods were run for each gene: aBSREL and BUSTED to check for positive selection in particular genes in WTD, and MEME to look for individual sites under positive selection. We independently tested selection for two sets of branches: all branches within WTD transmission clusters (called later on ‘clusters’); and clusters set plus singletons and the branches leading to transmission clusters (called ‘all’). While the clusters set represented the mutations happening only within the WTD population, the ‘all’ set was potentially contaminated by mutations that happened before the virus was transmitted to WTD, but it allowed incorporation of all available WTD samples. To search for sites under positive selection with MEME, we only looked for sites in foreground branches (e.g. included in ‘cluster’ or ‘all’ sets) with *p* < 0.001 in comparison with all other branches on the phylogenetic tree (background). The analyses for alpha and delta datasets were performed independently on the phylogenetic trees described in the previous section. Foreground branches were marked on the phylogenetic tree with phylotree.js (http://veg.github.io/phylotree.js/#). dN/dS value provided in Supplementary Table S6 were extracted from MEME output. Results of aBSREL and BUSTED outputs showed no signs of gene wise positive selection. As an alternative approach, we used codeml (from PAML version 4.9e^63^) to test whether there are signs of positive selection on branches leading for transmission clusters. Codeml was run in two modes with fix_omega =1 and fix_omega = 0. The LRT and p-values were calculated from obtained lnL values for each cluster. Again, no signs of gene wise positive selection were found. To study the frequencies of mutations in human populations for comparison to WTD, we utilized the outbreak.info package for R^64^ that utilizes GISAID data. Data was visualized with ggplot2 package for R 4.1.2.

### In vitro and in vivo experiments

#### Ethics statement

Animal studies were conducted in accordance with the Guide for the Care and Use of Laboratory Animals of the National Institutes of Health and approved under St. Jude Children’s Research Hospital’s Animal Care and Use Committee protocol 442.

#### Swabs

Positive swabs were received on dry ice and were transferred into the ABSL3+ at St. Jude Children’s Research Hospital. All virus isolation, characterization and animal experiments were performed under ABSL3+ conditions.

#### Virus isolation

The Vero E6 cell line ectopically expressing both TMPRSS2 and ACE2 (Vero ACE2 T2) was a kind gift from Dr. Barney Graham at VRC, NIAID, NIH. Cells were maintained in DMEM (Sigma D6429) supplemented with 10% heat treated fetal bovine serum (HyClone SH30071.03) and 10 μg/ml Puromycin (Sigma P9620). Cultures were overlaid with 1 mL of inoculum consisting of 100 μL of swab suspension plus 900 μL of infection media (DMEM supplemented with 2% heat treated fetal bovine serum and 1× antibiotic solution (Gibco 15240-062)). After 1 h, the inoculum was aspirated off and fresh infection media was added to the cells. Cultures were checked daily, and media-cell suspension was harvested when greater than 90% cytopathic effect (CPE) was observed. The suspension was tested by BD Veritor System for rapid detection of SARS-CoV-2 (Catalog # 256082) for confirmation of virus isolation and streaked on blood agar plates for sterility. Select viral isolates were sequenced to confirm identity with the original sample sequences that produced the viral isolates using CLC Genomic Workbench v22.0.1 (Qiagen) and BioEdit v7.2.5 (open source).

#### TCID_50_ assay

Virus stocks or experimental samples were titered using a Vero E6 cell line ectopically expressing the TMPRSS2 gene (Vero E6 T2) sourced from JCRB Cell Bank in Japan (https://cellbank.nibiohn.go.jp/english/). To determine the 50% tissue culture infectious dose (TCID_50_), 96-well culture plates were inoculated with 100 μL of a 1:10 serially diluted sample in infection media. After 72 h, the plates were fixed and stained with 0.1% crystal violet in 10% formalin. Infectious dose titers were determined using the Reed and Muench method^23^.

#### Vaccination

Male LVG Golden Syrian Hamster, 4–5 weeks of age, were purchased from Charles River Laboratories (Wilmington, MA), assigned numbers sequentially upon arrival to the Animal Resource Center, and assigned to groups based on vaccine treatment and virus challenge on paper without investigators observing individual animals. Laboratory experiments were performed exclusively with male animals, as designated by the vendor prior to arrival. Male animals were chosen as it has been established male Syrian hamsters are more susceptible to disease caused by SARS-CoV-2^29^. There were 10 total experimental groups with 120 total hamsters. Hamsters assigned to vaccine groups were vaccinated intramuscularly with 10 μg BNT162b2 vaccine (New York, NY), prepared as instructed with the exception that it had reached an expiration date and was no longer suitable for clinical use. Vaccine was administered in 50 μl at 2 injection sites (100 μl total volume) of the rear hind limb. Animals were boosted by the same procedure, on alternate limb, 21 days post vaccination (dpv). Sera was collected 21 days post boost (B + 21) and assessed for antibody response by microneutralization.

#### Animal challenge

Approximately 3 weeks post boost, vaccinated or naïve control hamsters were inoculated intranasally with 10^4^ TCID_50_ of SARS-Cov-2 virus. Each experimental group had 5 animals that were used for weight loss measurements, 4 animals that were sacrificed on 2-days post inoculation (dpi), and 3–4 animals that were sacrificed on 4 dpi for a total of 12-13 animals per group. Except the Human B.1.1.7 and Deer AY.25, which had 4, 3, and 3 animals for each of those time points respectively (10 total per group). These groups were limited by animal availability and included an unvaccinated control group for cross virus comparisons only (Fig. 5b). At 2 and 4 dpi, animals were sacrificed for lung and nasal turbinate. Alternatively, on 2, 4 and 6 dpi animals were anesthetized with 100 mg/kg Ketamine and nasal passages were rinsed with 0.5 mL phosphate buffered saline (PBS). Infectious viral load was determined by TCID_50_ as described. Longitudinal animals were scored for clinical signs and weighed daily for 2 weeks. All surviving animals were exsanguinated at 21 dpi and the serum was collected for microneutralization comparison. Animals were housed individually in standard filter top rat cages with day/night cycle from 6 a.m. to 6 p.m. Animal health observations were made at least 1/day or 2/day during peak infection. We did not observe any adverse events in these experiments other than the expected animal weight loss. Statistical analysis for all in vitro and in vivo experiments was performed using one-way ANOVA followed by a Tukey post hoc in Prism v.9 (GraphPad).

#### Neutralization assay

A viral microneutralization assay was performed to measure the neutralizing antibody activity of hamster sera of SARS-CoV-2/human/USA/WA-1/2020 (WA-1), hCoV-19/USA/CA_CDC 5574/2020 (Hu-B.1.1.7), SARS-CoV-2/human/USA/COR-21-192500/2021 (Hu-B.1.617.2), (hCoV-19/deer/USA/OH-OSU-2158/2021) (AY.103), hCoV-19/deer/USA/OH-OSU-1338/2021 (B.1.1.7-like), and BNT162b2 vaccine against representative WTD SARS-CoV-2 isolates. Fivefold serial dilutions were performed on heat inactivated sera (1 h at 56 °C) in infection medium, starting at a 1:40 dilution. A standardized amount of infectious SARS-CoV-2 virus (250 TCID_50_), diluted in infection medium, was added to the diluted serum at a 1:1 ratio and incubated for 1 h at 37 °C. A volume of 100 μL of the serum/virus mixture was added to Vero E6 T2 cells seeded in 96-well plates the previous day and incubated for 1 h at 37 °C under 5% CO_2_. Subsequently, an additional 100 μL of infection media was added and the cells incubated for a further 24–48 h. Following incubation, cells were fixed with 4% formaldehyde (Polysciences Cat #18814-20) for 30 min, washed with PBS (source) three times and then incubated with a block/permeabilization buffer (PBS supplemented with 3% Bovine Serum Albumin (BSA; Sigma-Aldrich Cat #A8327-500 ml) and 0.2% Triton-X-100 (ThermoFisherSurfact-Amps-X-100, 10% Solution Cat #28314)) for 30 min. Rabbit anti-SARS CoV-2 NP mAb (Sinobiologicals Cat # 40143-R040) at a 1:2000 dilution was added for 1 h. Cells were washed three times with PBS supplemented with 0.5% Tween (PBST; Thermofisher Cat #28314) before incubation with a secondary goat anti-rabbit IgG –HRP conjugated antibody (Cell Signaling Cat# 7074 S) at a 1:3000 dilution for 1 h. After washing the cells three times with PBST, 100 μL of TMB (Thermofisher Cat #N301) was added and color developed for 10 min before 1 N sulfuric acid (Fisher Scientific Cat #SA212-1) was added to stop the reaction. The optical density was measured at 450 nm on a Biotek Synergy plate microplate reader and the neutralization titers were calculated as the reciprocal serum dilution (IC_50_) causing 50% reduction of relative light units.

#### Growth kinetics

Vero E6 T2, Vero ACE2 T2, and Calu-3 cells were infected at a low multiplicity of infection (MOI 0.001 TCID_50_/cell) with representative WTD isolates of SARS-CoV-2 and parent viruses, washed, and maintained in infection medium. Supernatants were collected at 12, 24, 36, and 48 hpi, then titrated in Vero E6 T2 using TCID_50_. Titrations were calculated by the Reed and Muench method^65^. Data shown are mean titers ±SD of triplicate measures for each time point.

### Reporting summary

Further information on research design is available in the Nature Portfolio Reporting Summary linked to this article.

### Supplementary information

Supplementary Information

Peer Review File

Description of Additional Supplementary Files

Supplementary Data 1

Supplementary Data 2

Supplementary Data 3

Supplementary Data 4

Reporting Summary

## Data Availability

Whole-genome SARS-CoV-2 sequences are available on GenBank and raw sequence read data are available at NCBI SRA, accession numbers are available in Table S9. Available sequences for background data were downloaded from GISAID (acknowledged in Supplementary Data 3 and 4). The 787 genomes analyzed by Pekar et al.^21^ were used to compare for rate sensitivity analysis (10.1126/science.abp8337). The epidemiological curve of SARS-CoV-2 cases in humans in Ohio was generated using data available from the US Centers for Disease Control and Prevention (https://data.cdc.gov/Case-Surveillance/COVID-19-Case-Surveillance-Public-Use-Data-with-Ge/n8mc-b4w4). Wuhan SARS-CoV-2 genome NC_045512.2 was used as reference for the mutation analysis. Observed mutations on the spike trimer were visualized using the Protein Data Bank (PDB; rcsb.org), structure ID 7JJI. All other data are included in this article and its supplementary files.

## Code availability

Code generated for analysis is available from GitHub at https://github.com/garushyants/sars_cov_2_deer_Ohio (10.5281/zenodo.8137224)^66^.
