# Supplementary material for: Accelerated evolution of SARS-CoV-2 in free-ranging white-tailed deer
Source: Nat Commun. 2023 Aug 28;14:5105. doi: 10.1038/s41467-023-40706-y (PMC10462754; doi:10.1038/s41467-023-40706-y)
Supplement: Supplementary file 1 — Supplementary Information [file 41467_2023_40706_MOESM1_ESM.pdf]

Supplementary Materials

Table S1. Temporal, demographic, and harvest characteristics of the nasal swabs and serum samples collected from white-tailed deer are shown stratified by real-time reverse transcriptase PCR (rRT-PCR) and surrogate virus neutralization results.

	rRT-PCR results			Surrogate virus neutralization results		
	SARS-CoV-2 Negative	SARS-CoV-2 Positive	Estimated Prevalence	Sero-negative	Sero-positive	Estimated Sero-prevalence
Total	1359	163	10.7%	890	274	23.5%
Sex						
Female	649	60	8.5%	405	126	23.7%
Male	704	102	12.7%	481	145	23.2%
Age						
Adult	1063	136	11.3%	679	224	24.8%
Juvenile	273	25	8.4%	193	45	18.9%
Manner of death						
Culled	755	46	5.7%	421	124	22.8%
Hunter harvested	597	116	16.3%	464	149	24.3%
County urban classification						
Rural	716	121	14.5%	502	174	25.7%
Urban	643	42	6.1%	388	100	20.5%
Month of collection						
October	2	0	0.0%	0	0	
November	829	93	10.1%	578	172	22.9%
December	221	40	15.3%	118	50	29.8%
January	234	30	11.4%	148	40	21.3%
February	72	0	0.0%	46	11	19.3%
March	1	0	0.0%	0	1	100.0%

Table S2. Estimated SARS-CoV-2 prevalence is shown by county with total number of samples collected and urban/rural classification. Number of samples that were collected from white-tailed deer culled in population management programs are indicated.

County	Urban	Total Samples	Culled Deer Samples	Positive Samples	Prevalence
Adams	Rural	17	0	3	18%
Ashland	Rural	20	0	1	5%
Ashtabula	Rural	2	0	0	0%
Athens	Rural	33	0	12	36%
Belmont	Rural	3	0	2	67%
Brown	Urban	27	0	11	41%
Butler	Urban	6	0	0	0%
Carroll	Rural	4	0	0	0%
Champaign	Rural	10	0	2	20%
Clark	Rural	7	0	1	14%
Clermont	Urban	26	0	6	23%
Clinton	Rural	5	0	1	20%
Columbiana	Rural	4	0	1	25%
Coshocton	Rural	11	0	1	9%
Crawford	Rural	11	0	3	27%
Cuyahoga	Urban	183	179	1	1%
Darke	Rural	1	0	0	0%
Defiance	Rural	3	0	0	0%
Delaware	Urban	55	40	1	2%
Erie	Rural	179	177	2	1%
Fairfield	Urban	35	25	4	11%
Franklin	Urban	151	149	0	0%
Fulton	Rural	1	0	0	0%
Gallia	Rural	4	0	1	25%
Geauga	Urban	1	0	0	0%
Greene	Rural	11	0	0	0%
Guernsey	Rural	4	0	0	0%
Hamilton	Urban	9	0	1	11%
Hancock	Rural	1	0	0	0%
Hardin	Rural	4	0	0	0%
Harrison	Rural	5	0	3	60%
Henry	Rural	2	0	0	0%
Highland	Rural	9	0	4	44%
Hocking	Urban	12	0	3	25%
Holmes	Rural	8	0	1	13%

Huron	Rural	25	0	6	24%
Jackson	Rural	9	0	3	33%
Jefferson	Rural	3	0	0	0%
Knox	Rural	5	0	1	20%
Lawrence	Rural	1	0	1	100%
Licking	Urban	30	0	0	0%
Logan	Rural	23	0	1	4%
Lorain	Urban	40	35	2	5%
Lucas	Rural	43	41	13	30%
Madison	Urban	3	0	1	33%
Mahoning	Rural	2	0	0	0%
Marion	Rural	7	0	0	0%
Medina	Urban	9	0	3	33%
Meigs	Rural	14	0	5	36%
Miami	Rural	7	0	2	29%
Monroe	Rural	2	0	0	0%
Montgomery	Rural	1	0	0	0%
Morgan	Rural	16	0	0	0%
Morrow	Urban	5	0	0	0%
Muskingum	Rural	10	0	1	10%
Noble	Rural	8	0	1	13%
Ottawa	Rural	1	0	0	0%
Paulding	Rural	1	0	0	0%
Perry	Urban	4	0	1	25%
Pickaway	Urban	15	0	5	33%
Pike	Rural	8	0	0	0%
Portage	Rural	13	0	0	0%
Preble	Rural	3	0	0	0%
Putnam	Rural	1	0	0	0%
Richland	Rural	28	0	0	0%
Ross	Rural	3	0	2	67%
Sandusky	Rural	4	0	1	25%
Scioto	Rural	9	0	2	22%
Seneca	Rural	7	0	2	29%
Shelby	Rural	4	0	0	0%
Stark	Rural	14	0	2	14%
Summit	Rural	110	106	30	27%
Trumbull	Rural	2	0	1	50%
Tuscarawas	Rural	4	0	1	25%
Union	Urban	58	49	0	0%
Van Wert	Rural	1	0	0	0%

Vinton	Rural	2	0	1	50%
Warren	Urban	16	0	3	19%
Washington	Rural	47	0	5	11%
Wayne	Rural	11	0	0	0%
Williams	Rural	6	0	0	0%
Wood	Rural	4	0	0	0%
Wyandot	Rural	19	0	2	11%
Total		1522	801	163	11%

Table S3a. Results from mixed-effects logistic regression with random effects accounting for county level clustering is shown for all the covariates for which we adjusted. The reference category for each risk factor is indicated as the top row. October, February, and March were excluded from the model because no nasal swabs collected from white-tailed deer during those months tested positive for SARS-CoV-2 (Figure S1). The interaction term is presented as a model equation coefficient, not as an odds ratio for a specific covariate pattern. The Bonferroni corrected p-value cutoff for significance is 0.007.

Covariate	Categories	Model coefficient	Odds Ratio	95% Confidence interval OR	P-value (two-sided z test)
Urban county classification	Urban				
	Rural	2.69	14.7	2.2 – 100.0	0.006
WTD manner of death	Culled				
	Hunter-harvested	2.84	17.1	4.0 – 73.5	<0.0005
Interaction term for manner of death with urban county classification	Hunted*Rural	-2.90	0.055	0.008 – 0.397	0.004
Sex	Female				
	Male	0.42	1.5	1.0 – 2.2	0.034
Age	Adult				
	Juvenile	- 0.32	0.7	0.4 – 1.2	0.214
Month of collection	November				
	December	0.58	1.8	1.0 – 3.0	0.035
	January	- 0.17	0.8	0.2 – 3.8	0.821

Table S3b. The estimated prevalence and seroprevalence of SARS-CoV-2 is shown stratified based on the manner of death and the urban/rural designation of the county from which the WTD was harvested. Sample size for each category is indicated parenthetically.

		**County designation**		
	**Prevalence**			
		Rural	Urban	Total
**Manner of death**	Culled	13.3% (324)	0.6% (477)	5.7% (801)
	Hunted	15.2% (508)	19.0% (205)	16.3% (713)
	Total	14.4% (832)	6.2% (682)	10.7% (1514)
	**Seroprevalence**			
		Rural	Urban	Total
	Culled	23.6% (225)	22.2% (320)	22.8% (545)
	Hunted	26.8% (447)	17.5% (166)	24.3% (613)
	Total	25.7% (672)	20.6% (486)	23.6% (1158)

Table S4. SARS-CoV-2 viruses sequenced from white-tailed deer shown with county, date of collection, lineage, and variant data.

	Virus Name	Metropolitan Area	County	Environ-ment	Collection date	Lineage	Variant
1	hCoV-19/deer/USA/OH-OSU-1331/2021	Cincinnati–Wilmington–Maysville	Brown	Urban	11/13/2021	B.1.1.7	Alpha
2	hCoV-19/deer/USA/OH-OSU-1335/2021	Cincinnati–Wilmington–Maysville	Brown	Urban	11/13/2021	B.1.1.7	Alpha
3	hCoV-19/deer/USA/OH-OSU-1338/2021	Cincinnati–Wilmington–Maysville	Brown	Urban	11/13/2021	B.1.1.7	Alpha
4	hCoV-19/deer/USA/OH-OSU-2088/2021	Cincinnati–Wilmington–Maysville	Brown	Urban	11/30/2021	AY.103	Delta
5	hCoV-19/deer/USA/OH-OSU-2070/2021	Cincinnati–Wilmington–Maysville	Brown	Urban	12/4/2021	AY.103	Delta
6	hCoV-19/deer/USA/OH-OSU-2072/2021	Cincinnati–Wilmington–Maysville	Brown	Urban	12/4/2021	B.1.617.2	Delta
7	hCoV-19/deer/USA/OH-OSU-1271/2021	Cincinnati–Wilmington–Maysville	Clermont	Urban	11/6/2021	AY.103	Delta
8	hCoV-19/deer/USA/OH-OSU-1292/2021	Cincinnati–Wilmington–Maysville	Clermont	Urban	11/6/2021	AY.39	Delta
9	hCoV-19/deer/USA/OH-OSU-2083/2021	Cincinnati–Wilmington–Maysville	Clermont	Urban	11/30/2021	AY.103	Delta
10	hCoV-19/deer/USA/OH-OSU-2084/2021	Cincinnati–Wilmington–Maysville	Clermont	Urban	11/30/2021	AY.103	Delta
11	hCoV-19/deer/USA/OH-OSU-1297/2021	Cincinnati–Wilmington–Maysville	Warren	Urban	11/6/2021	B.1.617.2	Delta
12	hCoV-19/deer/USA/OH-OSU-2069/2021	Cincinnati–Wilmington–Maysville	Warren	Urban	12/4/2021	AY.103	Delta
13	hCoV-19/deer/USA/OH-OSU-1106/2021	Cleveland–Akron–Canton	Erie	Rural	11/15/2021	AY.25	Delta
14	hCoV-19/deer/USA/OH-OSU-1114/2021	Cleveland–Akron–Canton	Huron	Rural	11/13/2021	AY.25	Delta
15	hCoV-19/deer/USA/OH-OSU-1950/2021	Cleveland–Akron–Canton	Huron	Rural	11/29/2021	AY.25	Delta
16	hCoV-19/deer/USA/OH-OSU-2016/2021	Cleveland–Akron–Canton	Medina	Urban	11/30/2021	AY.25	Delta
17	hCoV-19/deer/USA/OH-OSU-2320/2021	Cleveland–Akron–Canton	Medina	Urban	12/4/2021	AY.25	Delta
18	hCoV-19/deer/USA/OH-OSU-2323/2021	Cleveland–Akron–Canton	Medina	Urban	12/4/2021	AY.25	Delta
19	hCoV-19/deer/USA/OH-OSU-1206/2021	Cleveland–Akron–Canton	Summit	Rural	11/13/2021	AY.25	Delta
20	hCoV-19/deer/USA/OH-OSU-0735/2022	Cleveland–Akron–Canton	Summit	Rural	1/19/2022	AY.3	Delta
21	hCoV-19/deer/USA/OH-OSU-0921/2022	Cleveland–Akron–Canton	Summit	Rural	1/19/2022	AY.3	Delta
22	hCoV-19/deer/USA/OH-OSU-0716/2022	Cleveland–Akron–Canton	Summit	Rural	1/20/2022	AY.103	Delta
23	hCoV-19/deer/USA/OH-OSU-0926/2022	Cleveland–Akron–Canton	Summit	Rural	1/20/2022	AY.103	Delta
24	hCoV-19/deer/USA/OH-OSU-2307/2021	Cleveland–Akron–Canton	Tuscarawas	Rural	12/4/2021	AY.103	Delta
25	hCoV-19/deer/USA/OH-OSU-2105/2021	Columbus–Marion–Zanesville	Fairfield	Urban	11/29/2021	AY.103	Delta
26	hCoV-19/deer/USA/OH-OSU-2126/2021	Columbus–Marion–Zanesville	Fairfield	Urban	11/29/2021	AY.103	Delta
27	hCoV-19/deer/USA/OH-OSU-2111/2021	Columbus–Marion–Zanesville	Fairfield	Urban	11/29/2021	AY.25	Delta
28	hCoV-19/deer/USA/OH-OSU-2125/2021	Columbus–Marion–Zanesville	Hocking	Urban	11/29/2021	AY.25	Delta
29	hCoV-19/deer/USA/OH-OSU-1214/2021	Columbus–Marion–Zanesville	Knox	Rural	11/13/2021	AY.118	Delta
30	hCoV-19/deer/USA/OH-OSU-1790/2021	Columbus–Marion–Zanesville	Licking	Urban	11/8/2021	B.1.617.2	Delta
31	hCoV-19/deer/USA/OH-OSU-2222/2021	Columbus–Marion–Zanesville	Logan	Rural	12/4/2021	AY.25	Delta
32	hCoV-19/deer/USA/OH-OSU-1434/2021	Columbus–Marion–Zanesville	Madison	Urban	11/6/2021	AY.25	Delta
33	hCoV-19/deer/USA/OH-OSU-2113/2021	Columbus–Marion–Zanesville	Perry	Urban	11/29/2021	AY.100	Delta
34	hCoV-19/deer/USA/OH-OSU-1459/2021	Columbus–Marion–Zanesville	Pickaway	Urban	11/13/2021	B.1.1.7	Alpha
35	hCoV-19/deer/USA/OH-OSU-1480/2021	Columbus–Marion–Zanesville	Pickaway	Urban	11/13/2021	B.1.1.7	Alpha
36	hCoV-19/deer/USA/OH-OSU-1490/2021	Columbus–Marion–Zanesville	Pickaway	Urban	11/13/2021	B.1.1.7	Alpha
37	hCoV-19/deer/USA/OH-OSU-1419/2021	Dayton–Springfield–Kettering	Champaign	Rural	11/13/2021	AY.25	Delta
38	hCoV-19/deer/USA/OH-OSU-1429/2021	Dayton–Springfield–Kettering	Champaign	Rural	11/13/2021	AY.25	Delta
39	hCoV-19/deer/USA/OH-OSU-1440/2021	Dayton–Springfield–Kettering	Clark	Rural	11/8/2021	AY.25	Delta
40	hCoV-19/deer/USA/OH-OSU-1710/2021	Dayton–Springfield–Kettering	Miami	Rural	12/4/2021	AY.103	Delta
41	hCoV-19/deer/USA/OH-OSU-1734/2021	Dayton–Springfield–Kettering	Miami	Rural	12/4/2021	AY.103	Delta

42	hCoV-19/deer/USA/OH-OSU-1111/2021	Mansfield–Ashland–Bucyrus	Crawford	Rural	11/22/2021	AY.25	Delta
43	hCoV-19/deer/USA/OH-OSU-2011/2021	Mansfield–Ashland–Bucyrus	Crawford	Rural	11/30/2021	AY.25	Delta
44	hCoV-19/deer/USA/OH-OSU-1983/2021	Parkersburg-Marietta-Vienna	Washington	Rural	11/29/2021	AY.3	Delta
45	hCoV-19/deer/USA/OH-OSU-1985/2021	Parkersburg-Marietta-Vienna	Washington	Rural	11/29/2021	AY.3	Delta
46	hCoV-19/deer/USA/OH-OSU-1988/2021	Parkersburg-Marietta-Vienna	Washington	Rural	11/29/2021	AY.3.1	Delta
47	hCoV-19/deer/USA/OH-OSU-1842/2021	Parkersburg-Marietta-Vienna	Washington	Rural	11/30/2021	AY.44	Delta
48	hCoV-19/deer/USA/OH-OSU-2184/2021	Parkersburg-Marietta-Vienna	Washington	Rural	12/4/2021	AY.25.1	Delta
49	hCoV-19/deer/USA/OH-OSU-1263/2021	Rural	Adams	Rural	11/13/2021	AY.25	Delta
50	hCoV-19/deer/USA/OH-OSU-1265/2021	Rural	Adams	Rural	11/13/2021	AY.3	Delta
51	hCoV-19/deer/USA/OH-OSU-1756/2021	Rural	Athens	Rural	11/6/2021	AY.3.1	Delta
52	hCoV-19/deer/USA/OH-OSU-1793/2021	Rural	Athens	Rural	11/7/2021	AY.44	Delta
53	hCoV-19/deer/USA/OH-OSU-1834/2021	Rural	Athens	Rural	11/30/2021	AY.20	Delta
54	hCoV-19/deer/USA/OH-OSU-1839/2021	Rural	Athens	Rural	11/30/2021	AY.25.1	Delta
55	hCoV-19/deer/USA/OH-OSU-1703/2021	Rural	Athens	Rural	11/30/2021	AY.44	Delta
56	hCoV-19/deer/USA/OH-OSU-1828/2021	Rural	Athens	Rural	11/30/2021	AY.44	Delta
57	hCoV-19/deer/USA/OH-OSU-1830/2021	Rural	Athens	Rural	11/30/2021	AY.44	Delta
58	hCoV-19/deer/USA/OH-OSU-1833/2021	Rural	Athens	Rural	11/30/2021	AY.44	Delta
59	hCoV-19/deer/USA/OH-OSU-1744/2021	Rural	Athens	Rural	11/30/2021	AY.75	Delta
60	hCoV-19/deer/USA/OH-OSU-1243/2021	Rural	Harrison	Rural	11/13/2021	AY.103	Delta
61	hCoV-19/deer/USA/OH-OSU-2249/2021	Rural	Harrison	Rural	12/4/2021	AY.103	Delta
62	hCoV-19/deer/USA/OH-OSU-1403/2021	Rural	Highland	Rural	11/8/2021	B.1.1.7	Alpha
63	hCoV-19/deer/USA/OH-OSU-2169/2021	Rural	Highland	Rural	12/4/2021	AY.109	Delta
64	hCoV-19/deer/USA/OH-OSU-2077/2021	Rural	Highland	Rural	12/4/2021	B.1.1.7	Alpha
65	hCoV-19/deer/USA/OH-OSU-1778/2021	Rural	Jackson	Rural	11/6/2021	AY.103	Delta
66	hCoV-19/deer/USA/OH-OSU-1439/2021	Rural	Jackson	Rural	11/13/2021	AY.39	Delta
67	hCoV-19/deer/USA/OH-OSU-2152/2021	Rural	Meigs	Rural	12/4/2021	AY.20	Delta
68	hCoV-19/deer/USA/OH-OSU-2153/2021	Rural	Meigs	Rural	12/4/2021	B.1.1.7	Alpha
69	hCoV-19/deer/USA/OH-OSU-2306/2021	Rural	Noble	Rural	12/4/2021	AY.25.1	Delta
70	hCoV-19/deer/USA/OH-OSU-1720/2021	Rural	Vinton	Rural	11/13/2021	AY.75	Delta
71	hCoV-19/deer/USA/OH-OSU-1099/2021	Rural	Wyandot	Rural	11/14/2021	AY.25	Delta
72	hCoV-19/deer/USA/OH-OSU-1100/2021	Rural	Wyandot	Rural	11/14/2021	AY.25	Delta
73	hCoV-19/deer/USA/OH-OSU-2156/2021	Toledo–Findlay-Tiffin	Lucas	Rural	12/7/2021	AY.103	Delta
74	hCoV-19/deer/USA/OH-OSU-2158/2021	Toledo–Findlay-Tiffin	Lucas	Rural	12/7/2021	AY.103	Delta
75	hCoV-19/deer/USA/OH-OSU-2324/2021	Toledo–Findlay-Tiffin	Lucas	Rural	12/7/2021	AY.103	Delta
76	hCoV-19/deer/USA/OH-OSU-2326/2021	Toledo–Findlay-Tiffin	Lucas	Rural	12/7/2021	AY.103	Delta
77	hCoV-19/deer/USA/OH-OSU-2327/2021	Toledo–Findlay-Tiffin	Lucas	Rural	12/7/2021	AY.103	Delta
78	hCoV-19/deer/USA/OH-OSU-1144/2021	Toledo–Findlay-Tiffin	Sandusky	Rural	11/15/2021	AY.3	Delta
79	hCoV-19/deer/USA/OH-OSU-1104/2021	Toledo–Findlay-Tiffin	Seneca	Rural	11/15/2021	B.1.617.2	Delta
80	hCoV-19/deer/USA/OH-OSU-2253/2021	Youngstown–Warren	Columbiana	Rural	12/4/2021	AY.103	Delta

Table S5. Evolutionary rates in white-tailed deer and humans (nucleotide subs/st/yr)

Entire genome

**Host**	**Dataset**	**Mean**	**95% HPD**
Deer	Alpha	1.8×10^{-3}	$1.6 – 2.0 \times 10^{-3}$
Human	Alpha	5.9×10^{-4}	$5.5 – 6.4 \times 10^{-4}$
Deer	Delta	1.6×10^{-3}	$1.5 – 1.8 \times 10^{-3}$
Human	Delta	6.0×10^{-4}	$5.9 – 6.6 \times 10^{-4}$
Human	*Pekar et al.*	1.3×10^{-3}	$1.1 – 1.6 \times 10^{-3}$

By genome partition

**Host**	**Dataset**	**Partition**	**Mean**	**95% HPD**
Deer	Alpha	ORF1a	1.92×10^{-3}	$1.64 - 2.19 \times 10^{-3}$
Deer	Alpha	ORF1b	1.40×10^{-3}	$1.12 – 1.67 \times 10^{-3}$
Deer	Alpha	S	1.38×10^{-3}	$1.02 – 1.74 \times 10^{-3}$
Deer	Alpha	ORF3-8, E, M	2.22×10^{-3}	$1.73 - 2.77 \times 10^{-3}$
Deer	Alpha	N	1.30×10^{-3}	$7.33 \times 10^{-4} – 1.91 \times 10^{-3}$
Deer	Delta	ORF1a	1.54×10^{-3}	$5.48 – 6.38 \times 10^{-4}$
Deer	Delta	ORF1b	1.27×10^{-3}	$1.08 – 1.45 \times 10^{-3}$
Deer	Delta	S	1.91×10^{-3}	$1.57 - 2.25 \times 10^{-3}$
Deer	Delta	ORF3-8, E, M	2.13×10^{-3}	$1.77 - 2.52 \times 10^{-3}$
Deer	Delta	N	2.03×10^{-3}	$1.52 – 2.57 \times 10^{-3}$
Human	Alpha	ORF1a	5.49×10^{-4}	$5.00 – 5.99 \times 10^{-4}$
Human	Alpha	ORF1b	4.65×10^{-4}	$4.12 – 5.15 \times 10^{-4}$
Human	Alpha	S	6.36×10^{-4}	$5.53 – 7.26 \times 10^{-4}$
Human	Alpha	ORF3-8, E, M	8.29×10^{-4}	$7.26 – 9.43 \times 10^{-4}$
Human	Alpha	N	1.09×10^{-3}	$9.04 \times 10^{-4} – 1.30 \times 10^{-3}$
Human	Delta	ORF1a	5.70×10^{-4}	$5.31 – 6.09 \times 10^{-4}$
Human	Delta	ORF1b	4.43×10^{-4}	$4.06 – 4.80 \times 10^{-4}$
Human	Delta	S	9.40×10^{-4}	$8.51 \times 10^{-4} – 1.03 \times 10^{-3}$
Human	Delta	ORF3-8, E, M	9.70×10^{-4}	$8.76 \times 10^{-4} – 1.06 \times 10^{-3}$
Human	Delta	N	8.76×10^{-4}	$7.53 \times 10^{-4} – 1.00 \times 10^{-3}$
Human	Early	ORF1a	1.15×10^{-3}	$9.35 \times 10^{-4} - 1.39 \times 10^{-3}$
Human	Early	ORF1b	1.12×10^{-3}	$8.81 \times 10^{-4} - 1.38 \times 10^{-3}$
Human	Early	S	1.44×10^{-3}	$1.08 - 1.82 \times 10^{-3}$
Human	Early	ORF3-8, E, M	1.65×10^{-3}	$1.23 - 2.12 \times 10^{-3}$

Human	Early	N	2.06×10^{-3}	$1.36 - 2.75 \times 10^{-3}$

Table S6. dN/dS values for four selected genes estimated with BUSTED. White-tailed deer – dN/dS within transmission clusters. Deer all – dN/dS for all white-tailed deer samples, Human – dN/dS for all human samples in dataset.

	**Delta**			**Alpha**		
	Deer all	Deer clusters	Human	Deer all	Deer clusters	Human
**N**	0.77	0.94	0.69	0.25	0.29	0.78
**nsp3**	0.47	0.69	0.60	0.17	0.19	0.59
**ORF3a**	1.26	0.51	1.38	0.68	4.30	1.54
**S**	0.40	0.65	0.84	0.38	0.46	0.57

Table S7. Prevalence of different types of mutations in white-tailed deer clusters and in humans. For both Alpha and Delta samples counts provided for transmission clusters including branches leading to clusters, and both values for all mutational events and synonymous mutations are provided. Data for human is provided based on states on the UShER phylogenetic tree (see Materials and Methods).

		**Delta Deer**				**Alpha Deer**				**Human**			
**REF**	**ALT**	**Syn**	**%(Syn)**	**All**	**%(All)**	**Syn**	**%(Syn)**	**All**	**%(All)**	**UShER_all**	**%(UShER)**	**UShER_syn**	**%(UShER_syn)**
A	C	7	3.17	4	0.92	0	0.00	3	0.67	77574	1.82	14112	0.82
A	G	0	0.00	18	4.14	7	2.82	19	4.22	414733	9.71	156893	9.15
A	T	1	0.45	9	2.07	3	1.21	6	1.33	70454	1.65	24353	1.42
C	A	3	1.36	11	2.53	0	0.00	4	0.89	108080	2.53	23273	1.36
C	G	1	0.45	2	0.46	0	0.00	3	0.67	24135	0.57	6895	0.40
C	T	176	79.64	299	68.74	202	81.45	318	70.67	1903905	44.59	904026	52.70
G	A	6	2.71	16	3.68	4	1.61	19	4.22	352838	8.26	96505	5.63
G	C	0	0.00	3	0.69	0	0.00	6	1.33	60632	1.42	4898	0.29
G	T	3	1.36	27	6.21	3	1.21	25	5.56	580823	13.60	92853	5.41
T	A	2	0.90	6	1.38	2	0.81	6	1.33	58581	1.37	29124	1.70
T	C	20	9.05	33	7.59	27	10.89	37	8.22	532552	12.47	344300	20.07
T	G	2	0.90	7	1.61	0	0.00	4	0.89	85567	2.00	18169	1.06

Table S8. Representative SARS-CoV-2 viral isolates which were selected for further characterization in vitro and in vivo.

Lineage	Virus Selected
AY.109 Delta (B.1.617.2-like)	hCoV-19/deer/USA/OH-OSU-2169/2021
AY.118 Delta (B.1.617.2-like)	hCoV-19/deer/USA/OH-OSU-1214/2021
AY.3 Delta (B.1.617.2-like)	hCoV-19/deer/USA/OH-OSU-1265/2021
AY.39 Delta (B.1.617.2-like)	hCoV-19/deer/USA/OH-OSU-1292/2021
AY.44 Delta (B.1.617.2-like)	hCoV-19/deer/USA/OH-OSU-1828/2021
AY.75 Delta (B.1.617.2-like)	hCoV-19/deer/USA/OH-OSU-1744/2021
B.1.1.7 Alpha (B.1.1.7-like)	hCoV-19/deer/USA/OH-OSU-1338/2021
AY.25 Delta (B.1.617.2-like)	hCoV-19/deer/USA/OH-OSU-1440/2021
AY.103 Delta (B.1.617.2-like)	hCoV-19/deer/USA/OH-OSU-2158/2021

Table S9. GenBank accession numbers for consensus sequences and SRA accession numbers for raw sequence read data for 80 white-tailed deer SARS-CoV-2 strains.

Virus Name	Consensus Sequence GenBank Accession	Sequence Read SRA Accession
hCoV-19/deer/USA/OH-OSU-0716/2022	OQ437913	SRR23645831
hCoV-19/deer/USA/OH-OSU-0735/2022	OQ437959	SRR23645830
hCoV-19/deer/USA/OH-OSU-0921/2022	OQ437948	SRR23645829
hCoV-19/deer/USA/OH-OSU-0926/2022	OQ437923	SRR23645828
hCoV-19/deer/USA/OH-OSU-1099/2021	OQ437920	SRR23645905
hCoV-19/deer/USA/OH-OSU-1100/2021	OQ437949	SRR23645904
hCoV-19/deer/USA/OH-OSU-1104/2021	OQ437932	SRR23645893
hCoV-19/deer/USA/OH-OSU-1106/2021	OQ437980	SRR23645882
hCoV-19/deer/USA/OH-OSU-1111/2021	OQ437956	SRR23645871
hCoV-19/deer/USA/OH-OSU-1114/2021	OQ437984	SRR23645860
hCoV-19/deer/USA/OH-OSU-1144/2021	OQ437969	SRR23645849
hCoV-19/deer/USA/OH-OSU-1206/2021	OQ437936	SRR23645838
hCoV-19/deer/USA/OH-OSU-1214/2021	OQ437978	SRR23645827
hCoV-19/deer/USA/OH-OSU-1243/2021	OQ437946	SRR23645826
hCoV-19/deer/USA/OH-OSU-1263/2021	OQ437912	SRR23645903
hCoV-19/deer/USA/OH-OSU-1265/2021	OQ437930	SRR23645902
hCoV-19/deer/USA/OH-OSU-1271/2021	OQ437915	SRR23645901
hCoV-19/deer/USA/OH-OSU-1292/2021	OQ437950	SRR23645900
hCoV-19/deer/USA/OH-OSU-1297/2021	OQ437990	SRR23645899
hCoV-19/deer/USA/OH-OSU-1331/2021	OQ437918	SRR23645898
hCoV-19/deer/USA/OH-OSU-1335/2021	OQ437958	SRR23645897
hCoV-19/deer/USA/OH-OSU-1338/2021	OQ437928	SRR23645896
hCoV-19/deer/USA/OH-OSU-1403/2021	OQ437921	SRR23645895

hCoV-19/deer/USA/OH-OSU-1419/2021	OQ437926	SRR23645894
hCoV-19/deer/USA/OH-OSU-1429/2021	OQ437971	SRR23645892
hCoV-19/deer/USA/OH-OSU-1434/2021	OQ437922	SRR23645891
hCoV-19/deer/USA/OH-OSU-1439/2021	OQ437981	SRR23645890
hCoV-19/deer/USA/OH-OSU-1440/2021	OQ437938	SRR23645889
hCoV-19/deer/USA/OH-OSU-1459/2021	OQ437960	SRR23645888
hCoV-19/deer/USA/OH-OSU-1480/2021	OQ437962	SRR23645887
hCoV-19/deer/USA/OH-OSU-1490/2021	OQ437957	SRR23645886
hCoV-19/deer/USA/OH-OSU-1703/2021	OQ437988	SRR23645885
hCoV-19/deer/USA/OH-OSU-1710/2021	OQ437967	SRR23645884
hCoV-19/deer/USA/OH-OSU-1720/2021	OQ437934	SRR23645883
hCoV-19/deer/USA/OH-OSU-1734/2021	OQ437929	SRR23645881
hCoV-19/deer/USA/OH-OSU-1744/2021	OQ437943	SRR23645880
hCoV-19/deer/USA/OH-OSU-1756/2021	OQ437970	SRR23645879
hCoV-19/deer/USA/OH-OSU-1778/2021	OQ437974	SRR23645878
hCoV-19/deer/USA/OH-OSU-1790/2021	OQ437976	SRR23645877
hCoV-19/deer/USA/OH-OSU-1793/2021	OQ437939	SRR23645876
hCoV-19/deer/USA/OH-OSU-1828/2021	OQ437961	SRR23645875
hCoV-19/deer/USA/OH-OSU-1830/2021	OQ437927	SRR23645874
hCoV-19/deer/USA/OH-OSU-1833/2021	OQ437982	SRR23645873
hCoV-19/deer/USA/OH-OSU-1834/2021	OQ437985	SRR23645872
hCoV-19/deer/USA/OH-OSU-1839/2021	OQ466563	SRR23645870
hCoV-19/deer/USA/OH-OSU-1842/2021	OQ437940	SRR23645869
hCoV-19/deer/USA/OH-OSU-1950/2021	OQ437977	SRR23645868
hCoV-19/deer/USA/OH-OSU-1983/2021	OQ437989	SRR23645867
hCoV-19/deer/USA/OH-OSU-1985/2021	OQ437975	SRR23645866
hCoV-19/deer/USA/OH-OSU-1988/2021	OQ437983	SRR23645865
hCoV-19/deer/USA/OH-OSU-2011/2021	OQ437947	SRR23645864
hCoV-19/deer/USA/OH-OSU-2016/2021	OQ437987	SRR23645863
hCoV-19/deer/USA/OH-OSU-2069/2021	OQ437979	SRR23645862
hCoV-19/deer/USA/OH-OSU-2070/2021	OQ437972	SRR23645861
hCoV-19/deer/USA/OH-OSU-2072/2021	OQ449344	SRR23645859
hCoV-19/deer/USA/OH-OSU-2077/2021	OQ437945	SRR23645858
hCoV-19/deer/USA/OH-OSU-2083/2021	OQ437937	SRR23645857
hCoV-19/deer/USA/OH-OSU-2084/2021	OQ437919	SRR23645856
hCoV-19/deer/USA/OH-OSU-2088/2021	OQ437917	SRR23645855
hCoV-19/deer/USA/OH-OSU-2105/2021	OQ437953	SRR23645854
hCoV-19/deer/USA/OH-OSU-2111/2021	OQ437935	SRR23645853
hCoV-19/deer/USA/OH-OSU-2113/2021	OQ437951	SRR23645852
hCoV-19/deer/USA/OH-OSU-2125/2021	OQ437942	SRR23645851
hCoV-19/deer/USA/OH-OSU-2126/2021	OQ437944	SRR23645850

hCoV-19/deer/USA/OH-OSU-2152/2021	OQ449343	SRR23645848
hCoV-19/deer/USA/OH-OSU-2153/2021	OQ437986	SRR23645847
hCoV-19/deer/USA/OH-OSU-2156/2021	OQ437924	SRR23645846
hCoV-19/deer/USA/OH-OSU-2158/2021	OQ437925	SRR23645845
hCoV-19/deer/USA/OH-OSU-2169/2021	OQ437941	SRR23645844
hCoV-19/deer/USA/OH-OSU-2184/2021	OQ437914	SRR23645843
hCoV-19/deer/USA/OH-OSU-2222/2021	OQ437954	SRR23645842
hCoV-19/deer/USA/OH-OSU-2249/2021	OQ437963	SRR23645841
hCoV-19/deer/USA/OH-OSU-2253/2021	OQ437952	SRR23645840
hCoV-19/deer/USA/OH-OSU-2306/2021	OQ437955	SRR23645839
hCoV-19/deer/USA/OH-OSU-2307/2021	OQ437916	SRR23645837
hCoV-19/deer/USA/OH-OSU-2320/2021	OQ437933	SRR23645836
hCoV-19/deer/USA/OH-OSU-2323/2021	OQ437968	SRR23645835
hCoV-19/deer/USA/OH-OSU-2324/2021	OQ437973	SRR23645834
hCoV-19/deer/USA/OH-OSU-2326/2021	OQ437964	SRR23645833
hCoV-19/deer/USA/OH-OSU-2327/2021	OQ437931	SRR23645832

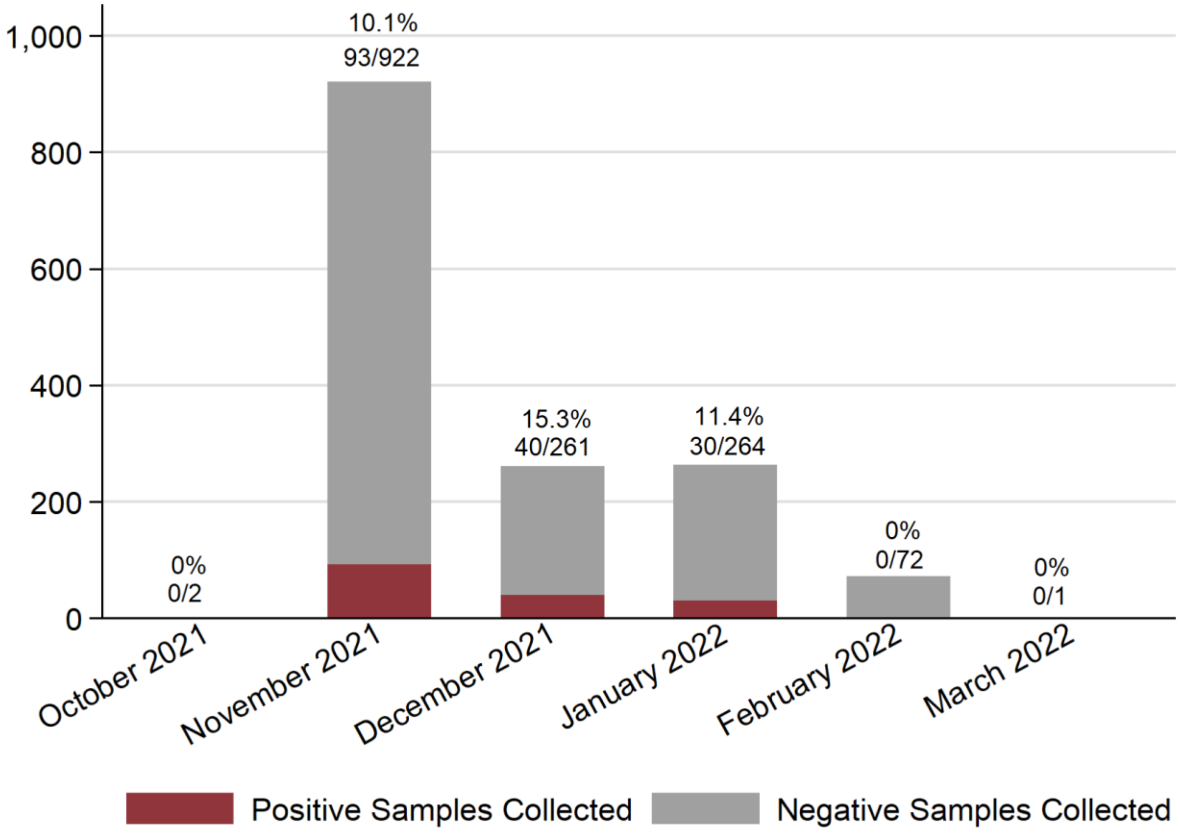

Figure S1. Number of nasal swabs collected from white-tailed deer is shown by month of collection with rRT-PCR positive samples shown in red and negative samples in gray. Number of positive samples and total sample size for each month is indicated above each bar along with estimated SARS-CoV-2 prevalence.

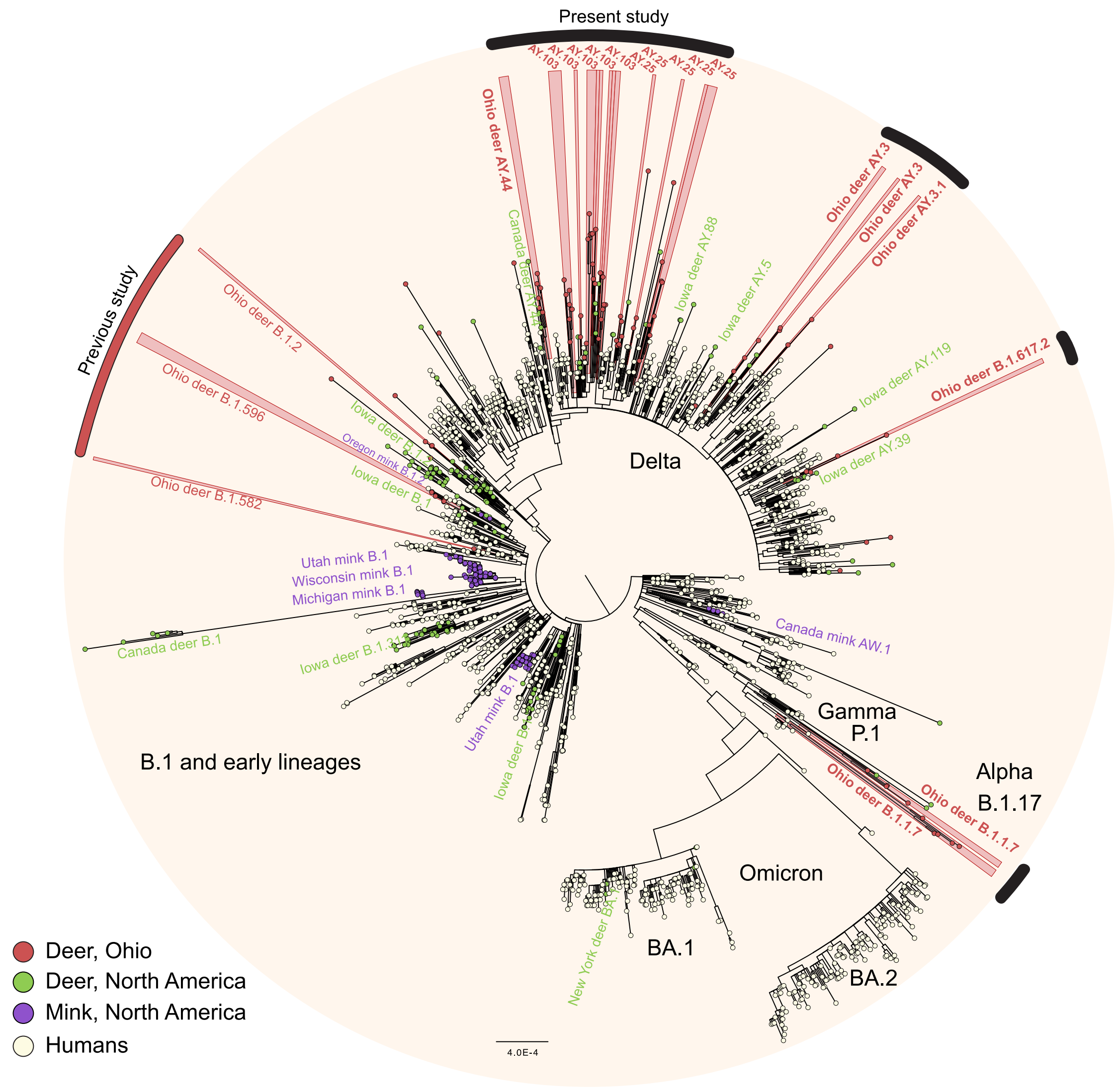

Figure S2. Maximum likelihood phylogenetic tree of SARS-CoV-2 in humans, mink, and white-tailed deer in North America. Tips (circles) are shaded by host species and location. Viruses from white-tailed deer in Ohio (red circles) are labeled to indicate whether they were collected from our previous study (B.1.2, B.1.596, and B.1.582) or the present study (delta and alpha).

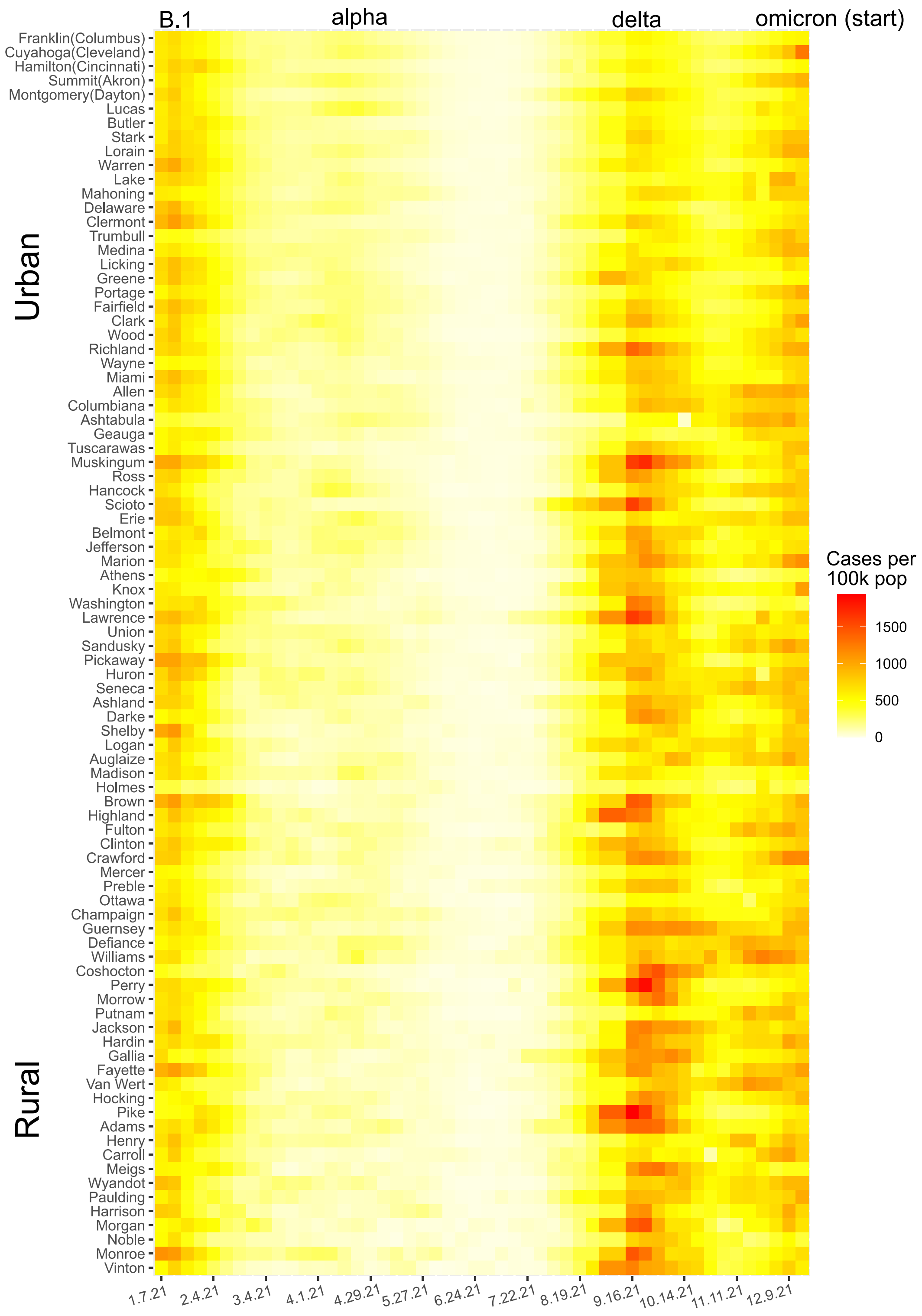

Figure S3. Heat map of human COVID-19 cases over time and space in Ohio. For each Ohio county (y-axis), the number of COVID-19 cases per 100,000 population is indicated over time (weekly, x-axis) by the shade of the box (red = high, white = low). Ohio counties listed on the y-axis are ordered by population size, from the largest, most urban counties (top) to the smallest, most rural counties (bottom).

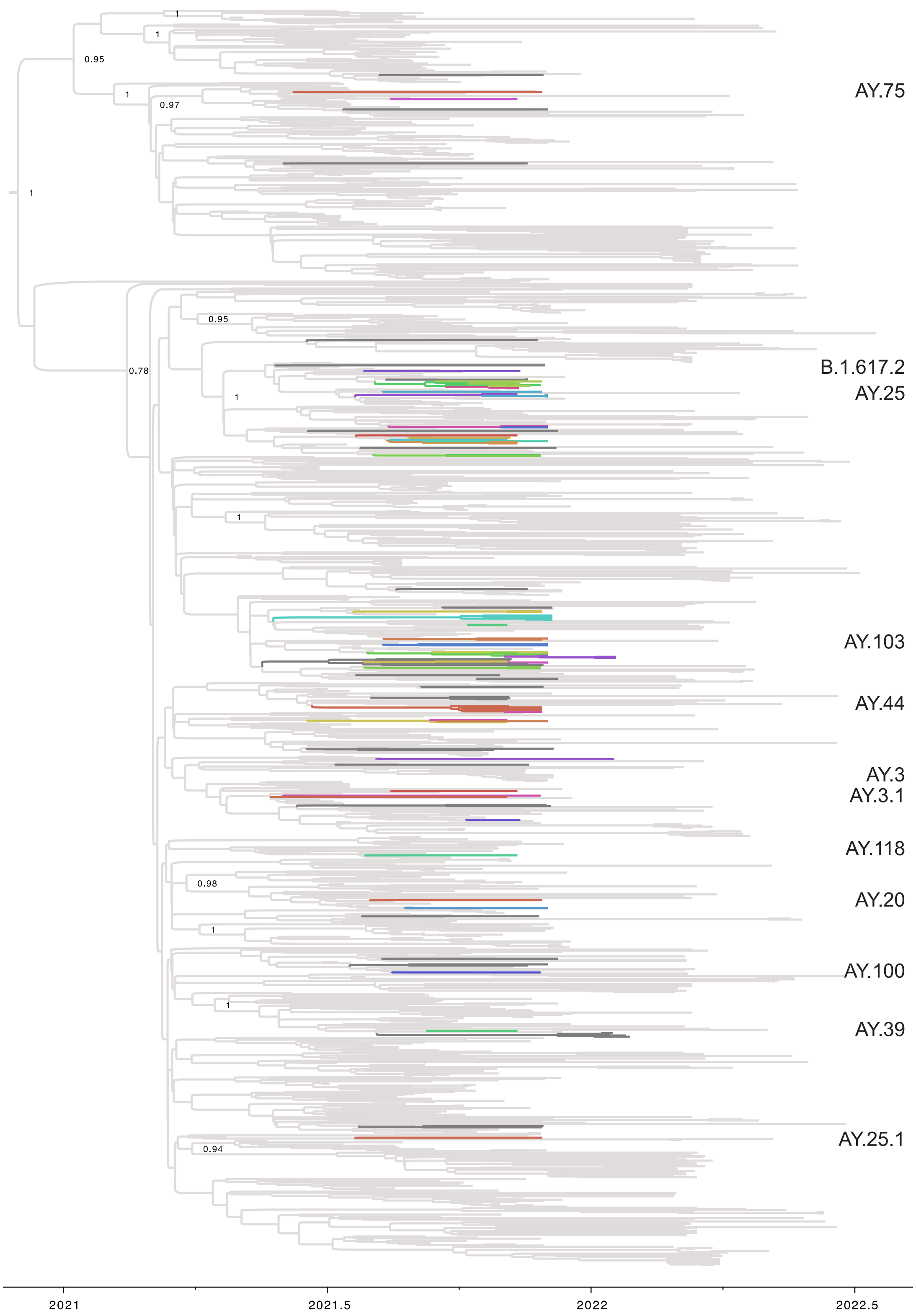

Figure S4. Time-scaled MCC tree inferred for delta variant viruses in humans and white-tailed deer in Ohio. Branches shaded by host species: gray = human, dark gray = deer from outside Ohio, colored branches = Ohio deer. Ohio deer branches are colored by county. Posterior probabilities are provided for

key nodes. PANGO lineages for Ohio deer viruses are listed. Raw tree file is available in GitHub repository.

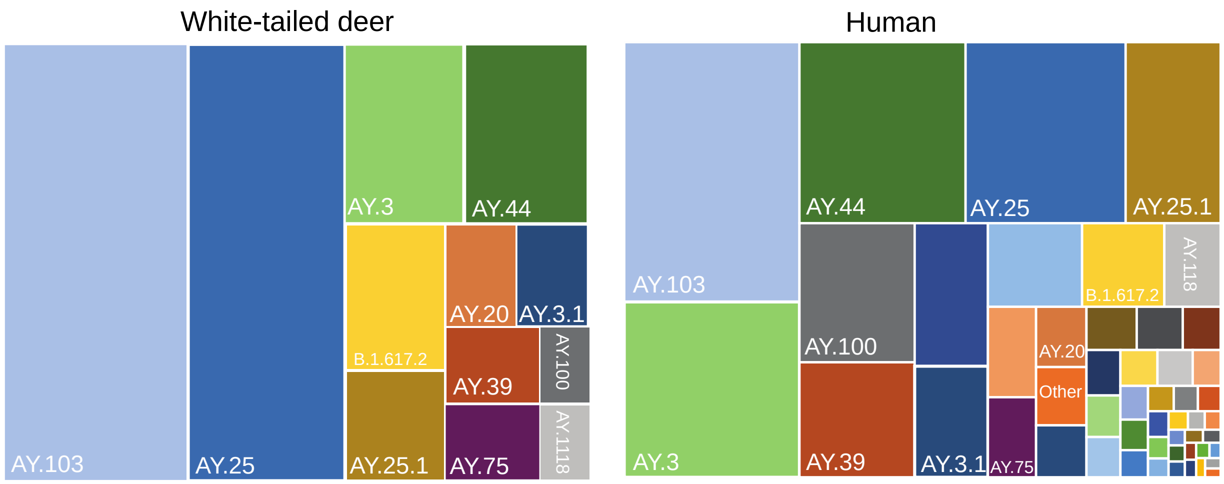

Figure S5. Dominant delta variant lineages in humans and white-tailed deer in Ohio. The size of the box is proportional to the number of viruses belonging to the Pango lineage in white-tailed deer (left) and humans (right) in Ohio.

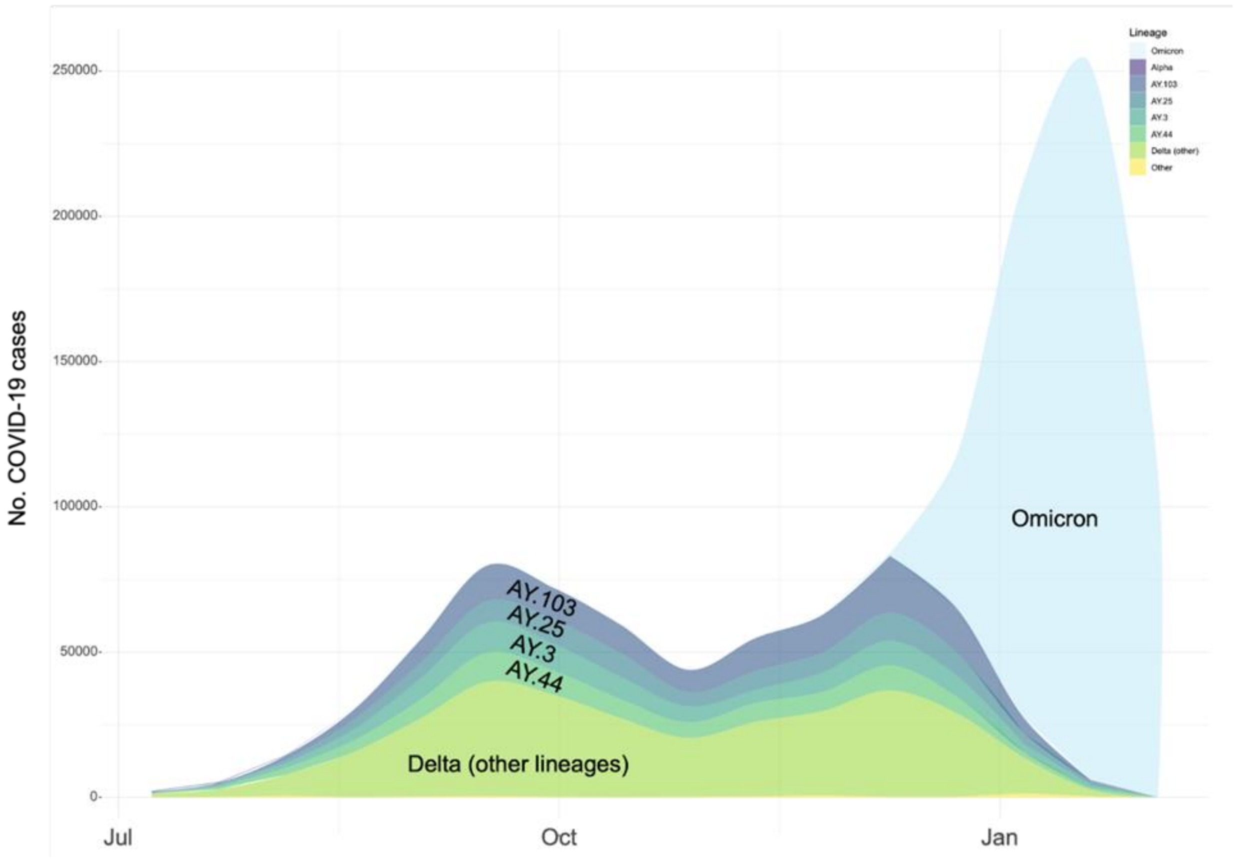

Fig S6. Co-circulation of delta variant lineages in humans in Ohio. The number of weekly COVID-19 cases in humans from July 1, 2021 to Jan 22, 2022. Shading indicates the proportion of 22,200 sequenced human viruses in Ohio, categorized as collected for "baseline surveillance," that belong to one of the four dominant AY lineages of the delta variant, omicron, or one of the other lineages of the delta variant.

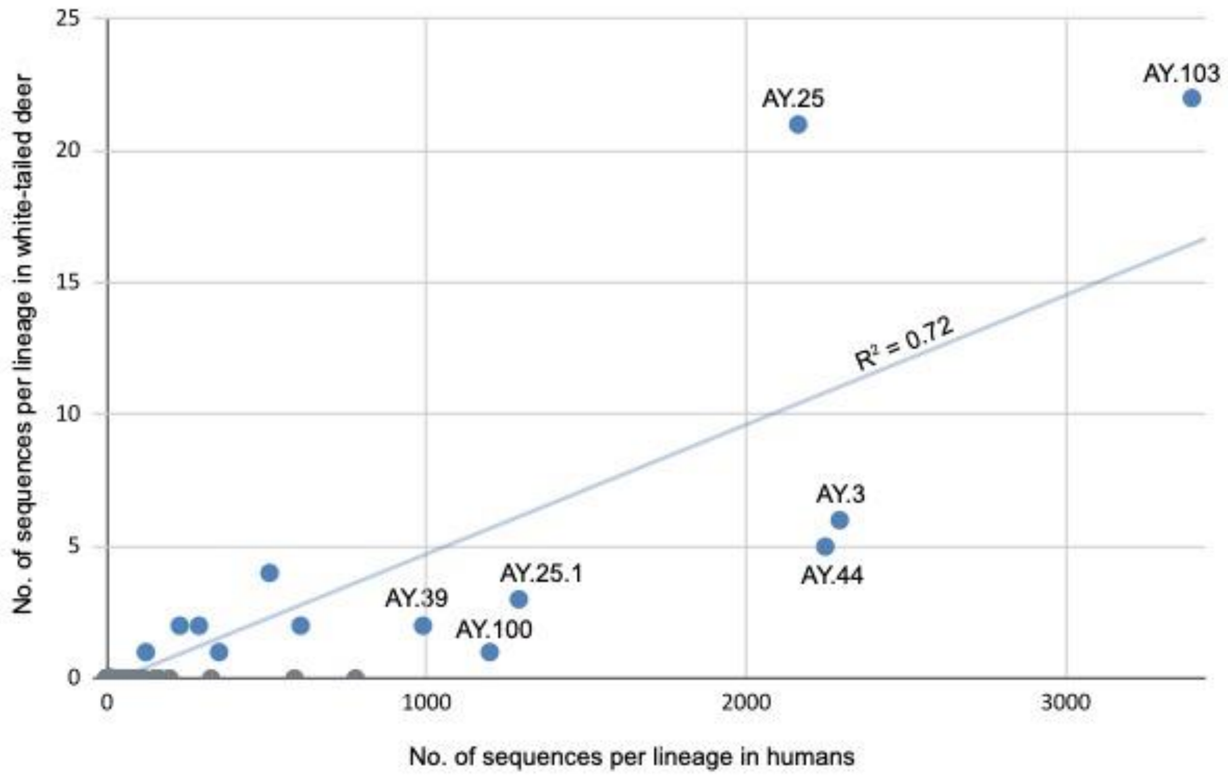

Figure S7. Plot of delta variant lineage frequency in humans and white-tailed deer in Ohio. The x-axis represents the number of sequences from SARS-CoV-2 viruses collected in humans in Ohio that belong to AY PANGO lineages. The y-axis represents the number of sequences from SARS-CoV-2 viruses collected in deer in Ohio for this study that belong to AY PANGO lineages.

		Week												
		44	45	46	47	48	49	50	51	52	1	2	3	Total
Cincinnati	Brown		B.1.1.7			AY.103<br>B.1.617.2								3
	Clermont	AY.39/103				AY.103								2
	Warren	B.1.617.2				AY.103								2
Cleveland	Erie			AY.25										1
	Huron		AY.25			AY.25								1
	Medina					AY.25								1
	Summit		AY.25										AY.3/103	3
	Tuscarawas					AY.103								1
Columbus	Fairfield					AY.25/103								2
	Hocking					AY.25								1
	Knox		AY.118											1
	Licking		B.1.617.2											1
	Logan					AY.25								1
	Madison	AY.25												1
	Perry					AY.100								1
	Pickaway		B.1.1.7											1
Dayton	Champaign		AY.25											1
	Clark		AY.25											1
	Miami					AY.103								1
Toledo	Lucas						AY.103							1
	Sandusky			AY.3										1
	Seneca			B.1.617.2										1
	Adams		AY.25/3											2
	Athens	AY.3.1/44				AY.20/25.1/44/75								5
	Columbiana					AY.103								1
	Crawford				AY.25	AY.25								1
	Harrison		AY.103			AY.103								1
	Highland		B.1.1.7			AY.109<br>B.1.1.7								2
	Jackson	AY.103	AY.39											2
	Meigs					AY.20<br>B.1.1.7								2
	Noble					AY.25.1								1
	Vinton		AY.75											1
	Washington					AY.25.1/3/3.1/44								4
	Wyandot		AY.25											1

Figure S8. Co-circulation of SARS-CoV-2 lineages in Ohio white-tailed deer by week and county. SARS-CoV-2 lineages identified in white-tailed deer per county per week (week 44 begins November 1, 2021)**.** Counties located within that larger metropolitan areas are listed first, followed by rural counties.

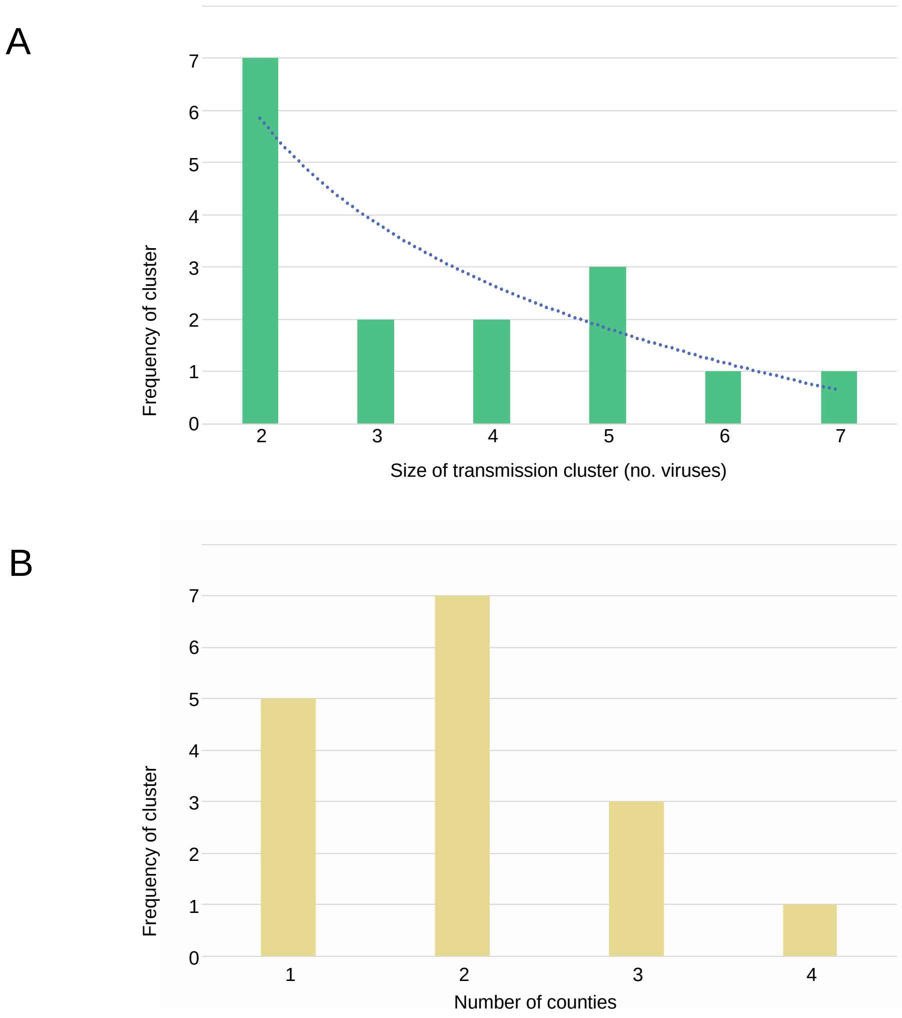

Figure S9. Size of white-tailed deer transmission clusters. (A) The frequency of deer transmission clusters of different sizes (minimum size = 2 viruses; maximum size = 7 viruses) is presented on the y-axis. (B) The frequency of deer transmission clusters spanning different numbers of counties (minimum size = 1 county; maximum size = 4 counties).

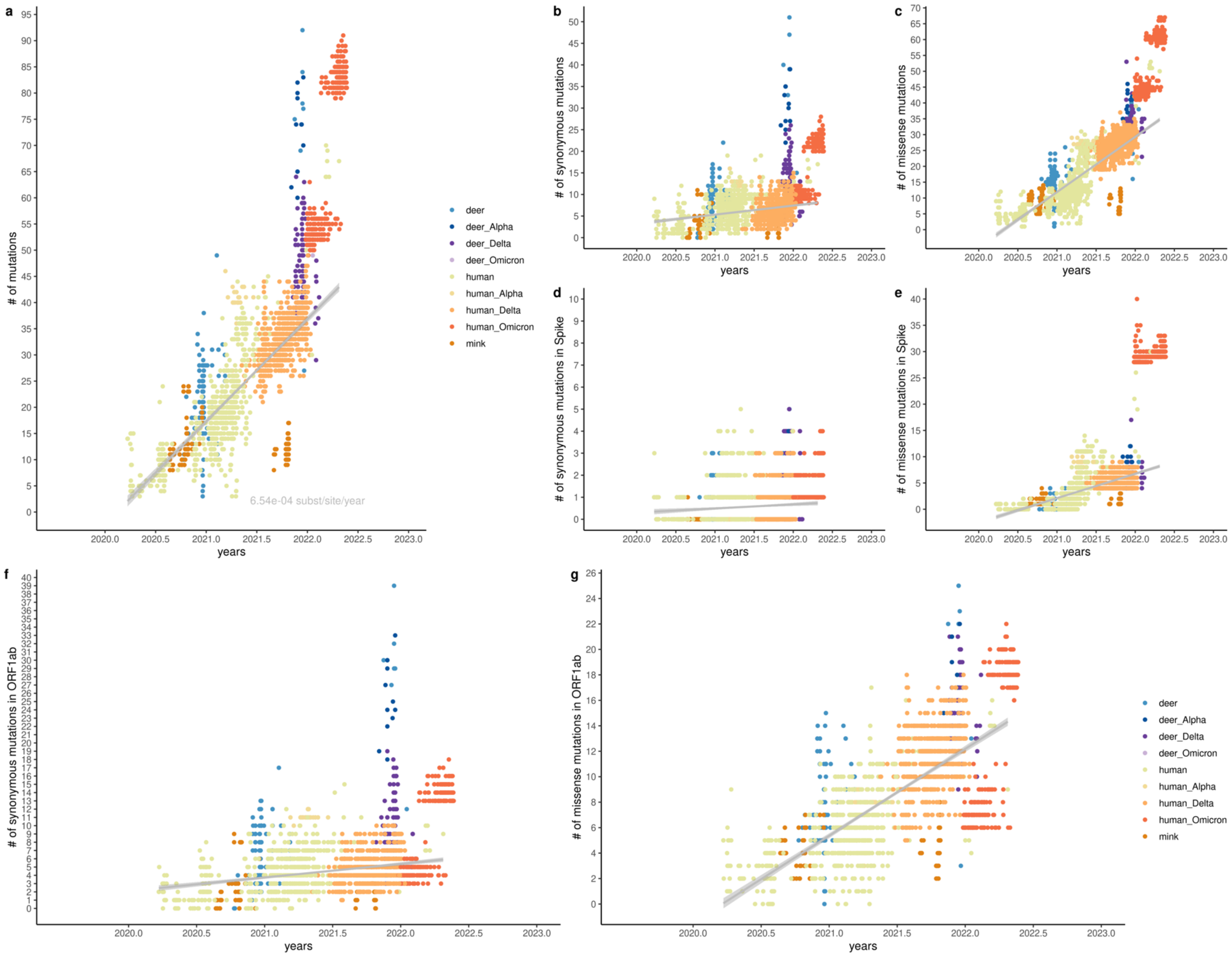

Figure S10. Root-to-tip regression for the global SARS-CoV-2 dataset in humans, mink, and white-tailed deer in North America. Each plot shows the number of mutations accumulated from root to leaves versus collection date of leaves. Each point (representing one leaf on the phylogenetic tree) colored by the category it belongs to: white-tailed deer samples from either alpha (dark blue), delta (purple) or omicron(violet) VOCs or other lineages (light blue), human samples from alpha (dark green), delta (light orange) or omicron (dark orange) or other lineages (light green), or mink samples (orange). (a) number of all mutations in the whole genome, (b) only synonymous mutations and (c) missense mutations. Separately we considered synonymous and missense mutations in Spike (d, e) and ORF1ab (f, g). The state at each node on the global ML tree (Figure S2) was reconstructed with TreeTime.

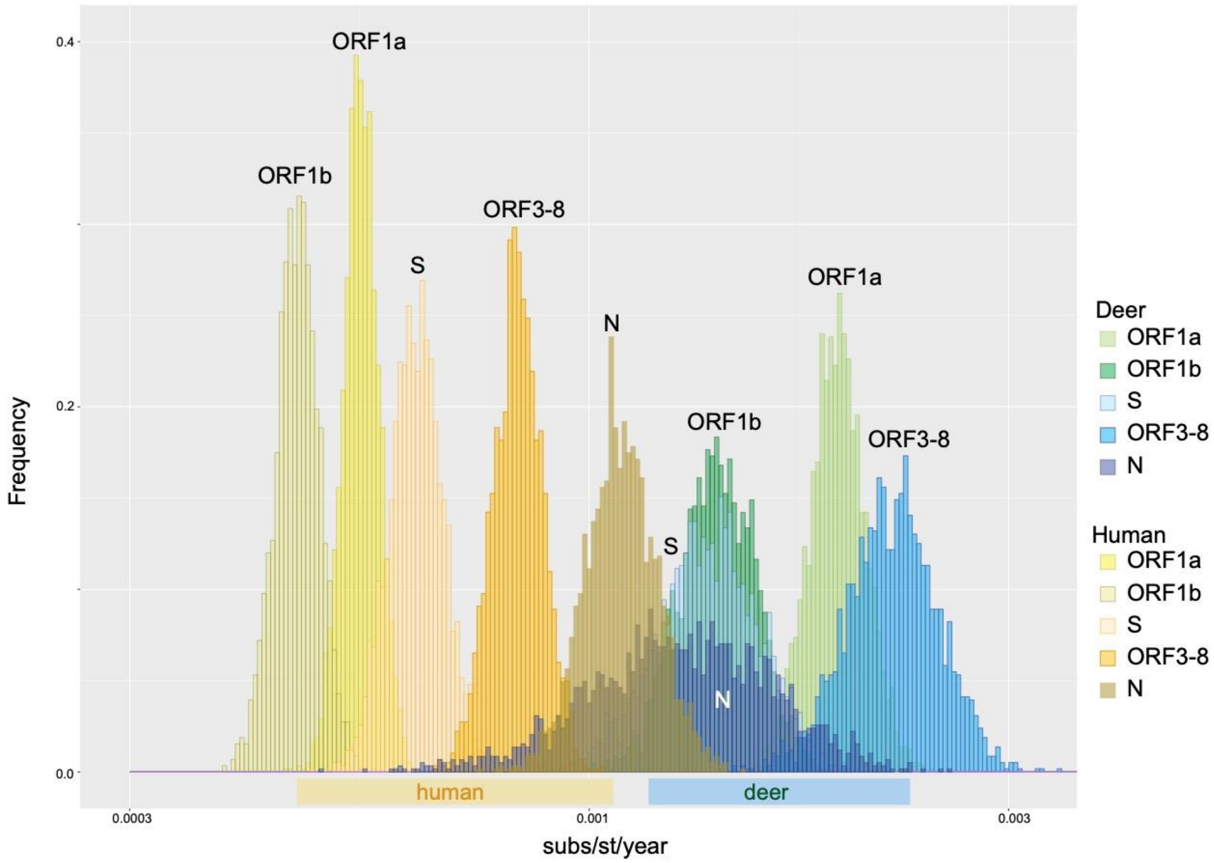

Figure S11. Evolutionary rates of alpha in humans and white-tailed deer. The posterior distributions of evolutionary rates (substitutions per site per year) for five partitions of the SARS-CoV-2 genome (ORF1a, ORF1b, ORF3 – ORF8, spike (S), and nucleocapsid (N) are presented for human (yellow/brown shades) and white-tailed deer (green/blue shades) for the alpha variant. Similar plot for delta variant is available in Figure 4C.

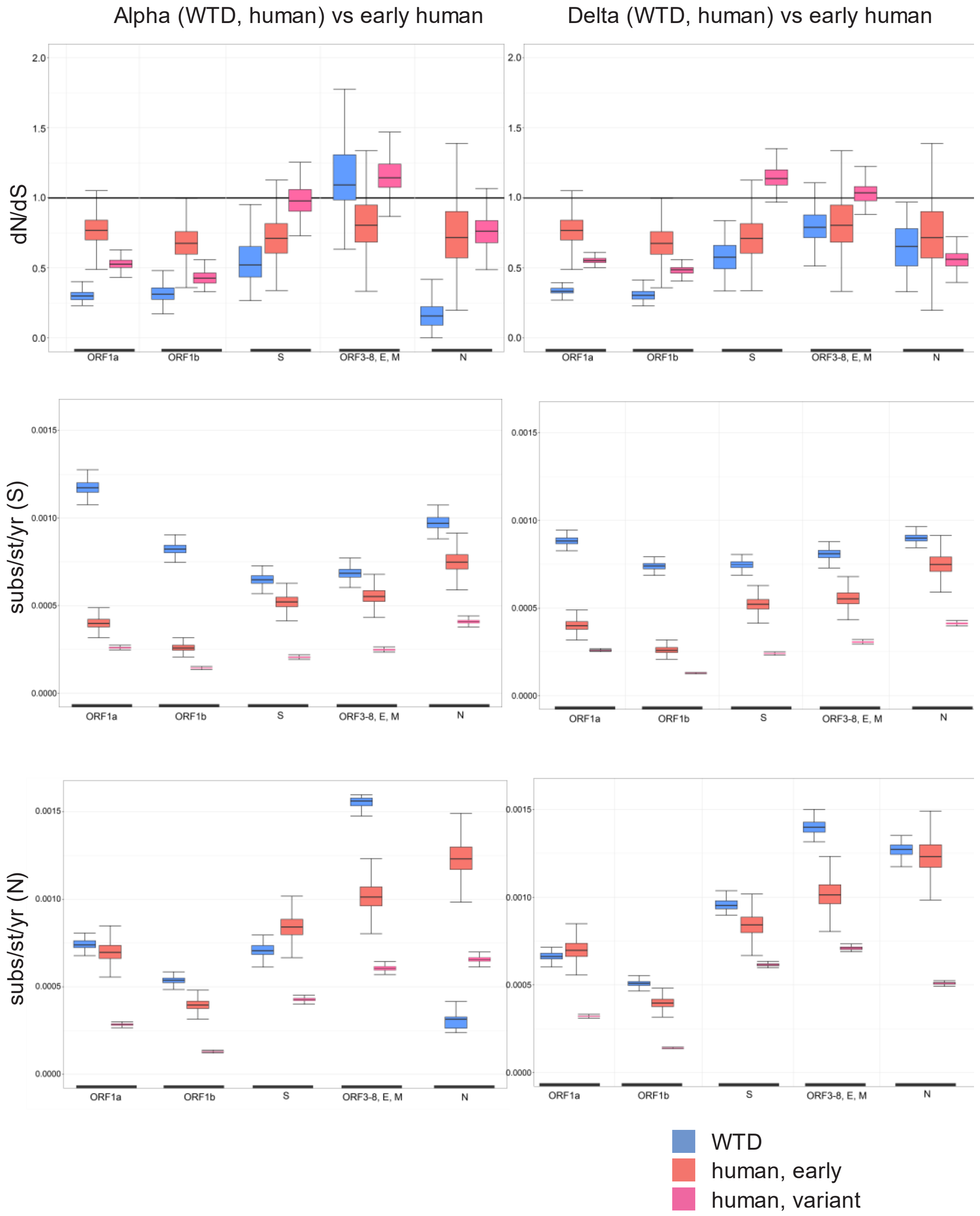

Figure S12. Rates of synonymous and non-synonymous substitution for alpha and delta variants in humans and white-tailed deer. The ratio of non-synonymous to synonymous substitution rates (dN/dS) is presented for the alpha (left column, n = 786) and delta (right column, n = 1094) variants for deer (blue boxes) and humans (pink boxes), as well as 786 early strains of SARS-CoV-2 in humans from Pekar et al.[1] Data is presented for five genome partitions: open reading frame 1a, open reading frame 1b, spike protein, open reading frames 3-8 with envelope and membrane proteins, and nucleocapsid. Whisker plots present the mean value, upper and lower quartiles, and minimum and maximum. A black line where dN/dS = 1 indicates the threshold between positive and purifying selection. Synonymous substitutions (S) per site per year is presented in the middle row and non-synonymous substitutions (N) per site per year is presented in the lowest row.

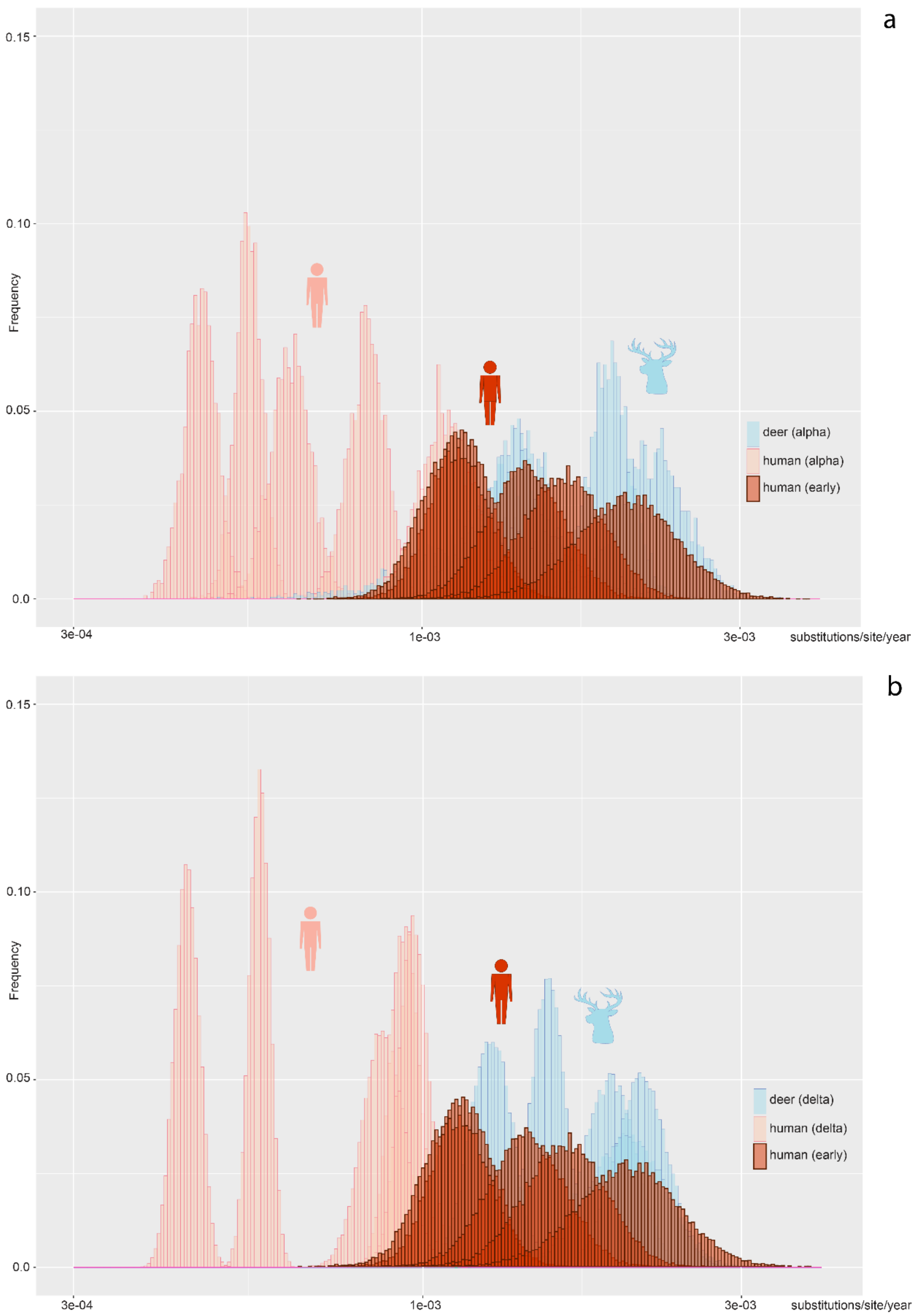

Figure S13. Evolutionary rates during early phase of SARS-CoV-2 outbreak in humans. The posterior distributions of evolutionary rates (substitutions per site per year) for five partitions of the SARS-CoV-2 genome (ORF1a, ORF1b, ORF3 – ORF8, spike (S), and nucleocapsid (N) are presented for three datasets: variant in white-tailed deer (blue); variant in humans (pink); and 786 early strains of SARS-CoV-

2 in humans from Pekar et al.[1] Alpha is presented above (n = 786) and delta below (n = 1094). Similar plots are available for variant data only (human vs. deer) for the alpha variant (Figure S11) and delta variant (Figure 4C). Mean values and 95% HPD are available for each partition and dataset in Table S5.

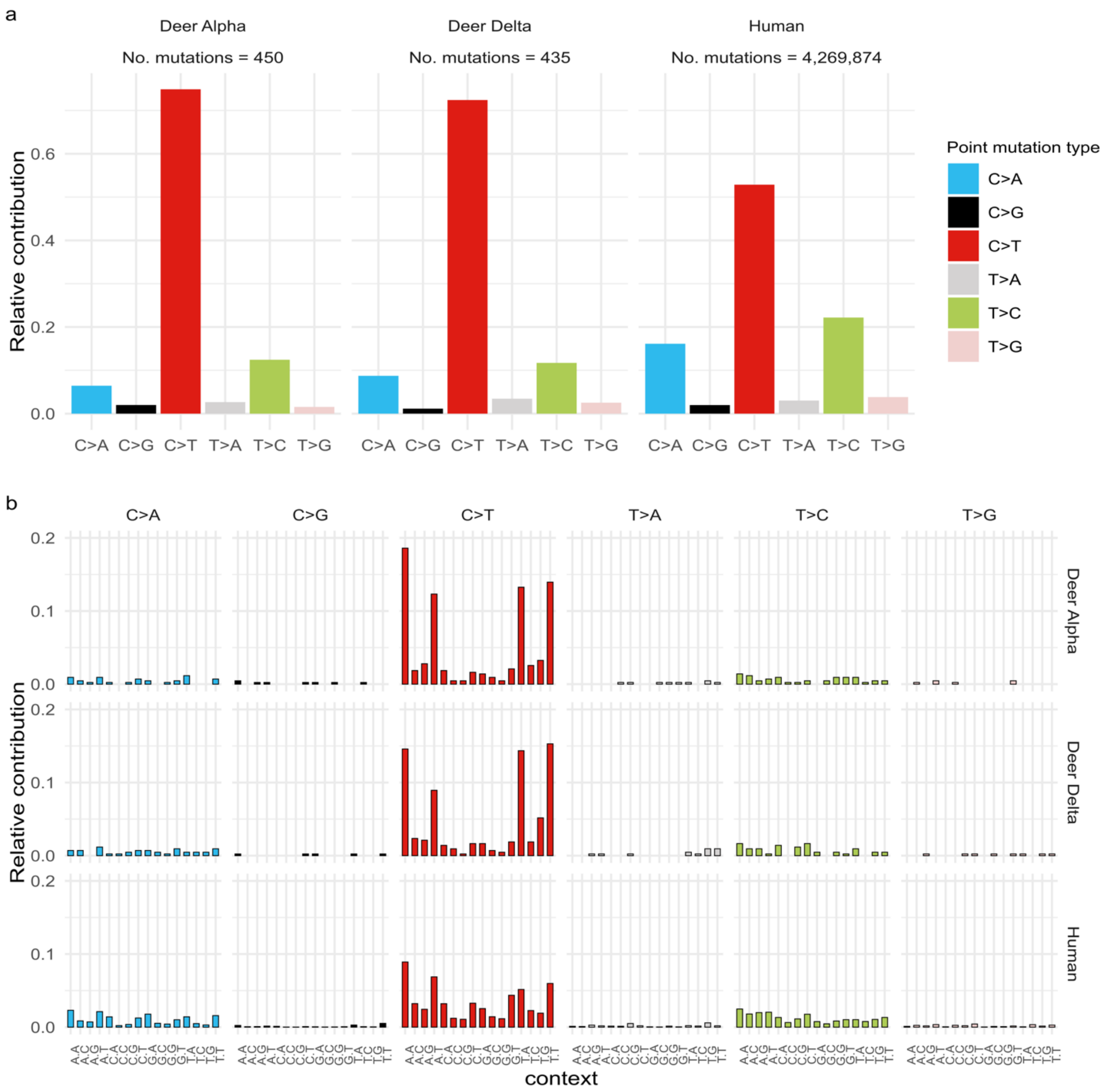

Figure S14. Mutational spectrum for all types of mutations in white-tailed deer and human. (a) Single nucleotide substitutions (b) Three letter contexts. Mutations in deer calculated for deer clusters (including branches leading to clusters): Delta white-tailed deer – Ohio white-tailed deer clusters from Delta dataset; Alpha white-tailed deer – all white-tailed deer clusters from Alpha dataset. Data for human is extracted from public version of UShER tree (see Materials and Methods).

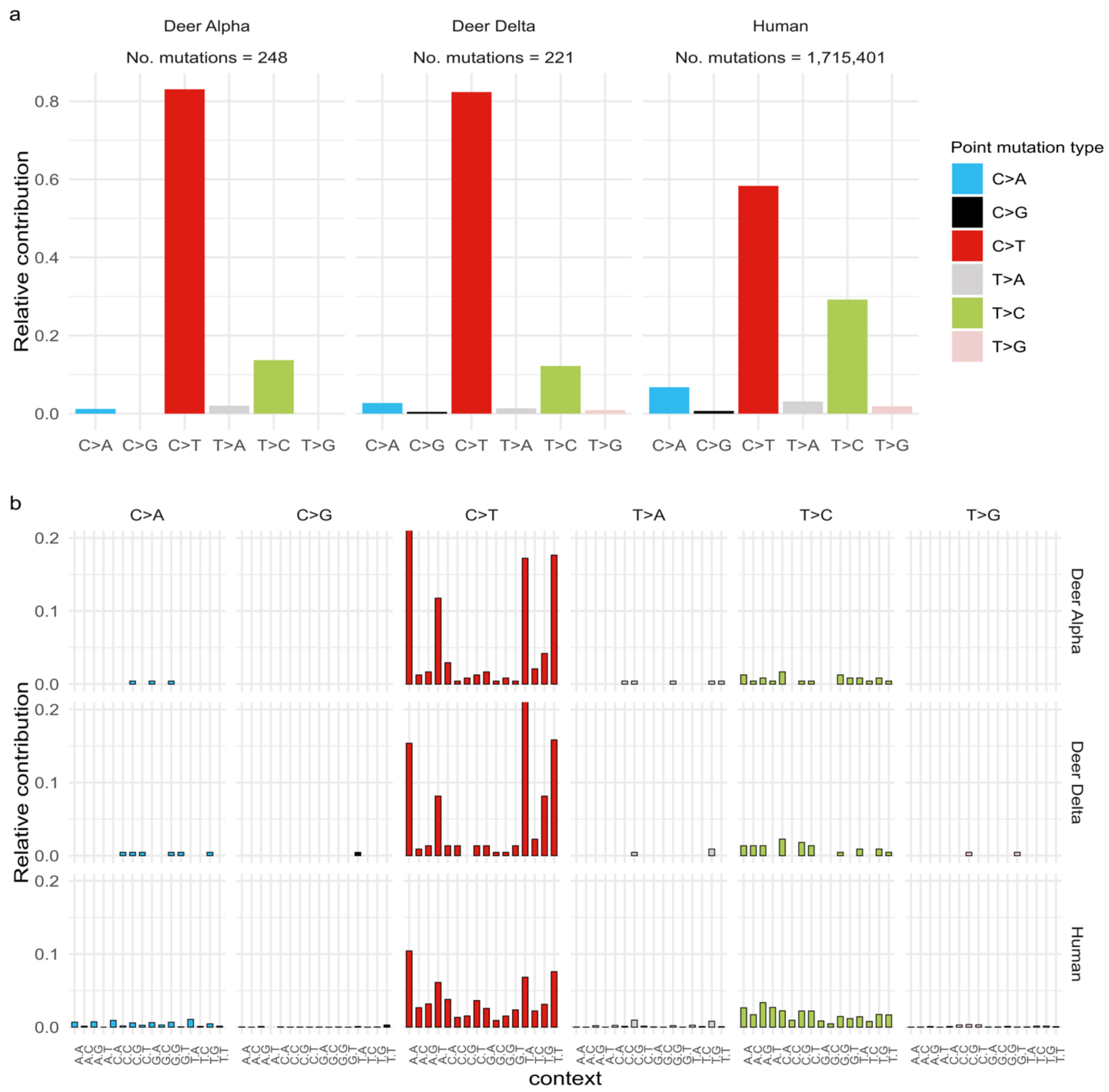

Figure S15. Mutational spectrum for synonymous mutations only in white-tailed deer and human. (a) Single nucleotide substitutions (b) Three letter contexts. Mutations in white-tailed deer calculated for white-tailed deer clusters (including branches leading to clusters): Delta white-tailed deer – Ohio white-tailed deer clusters from Delta dataset; Alpha white-tailed deer – all white-tailed deer clusters from Alpha dataset.

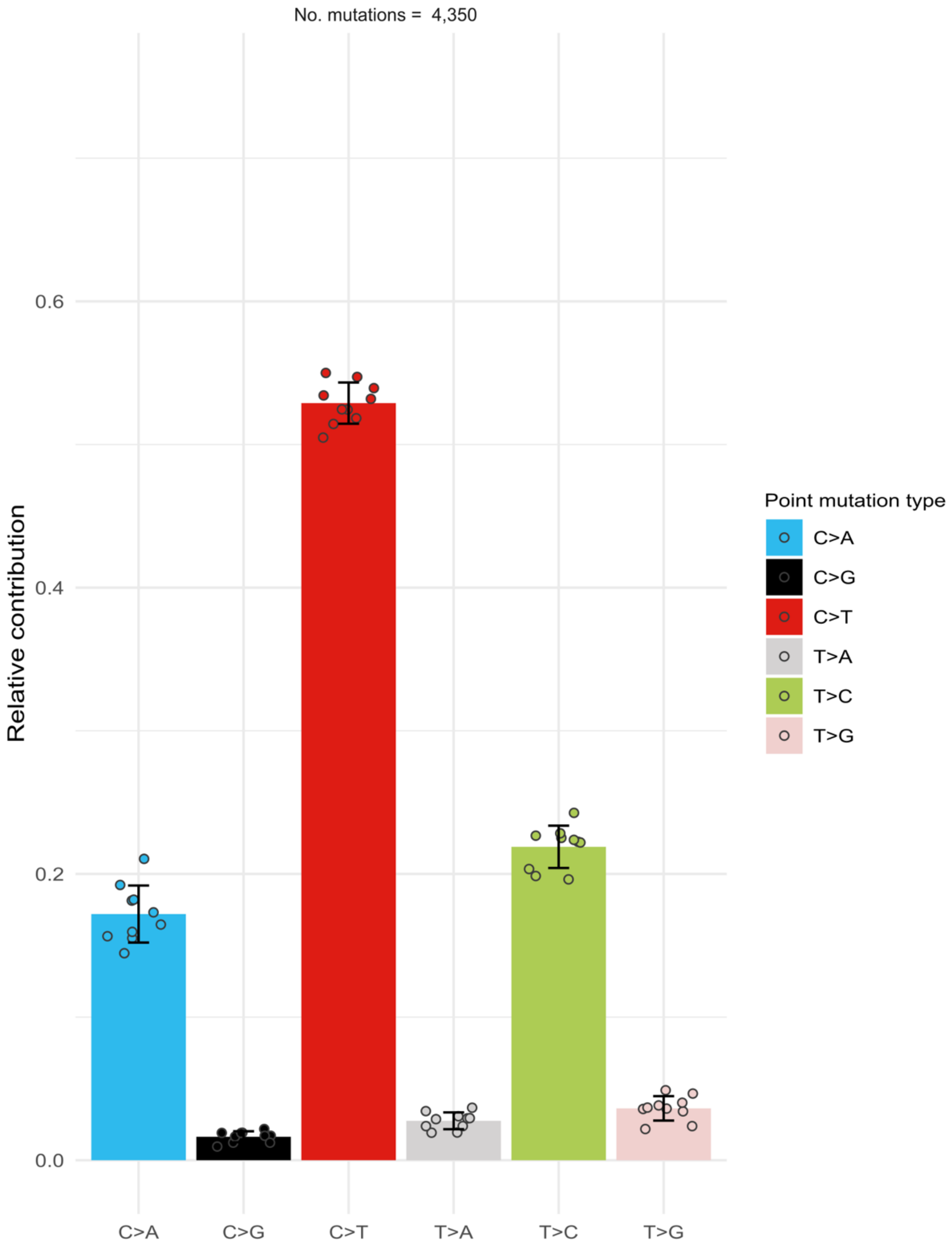

Figure S16. Low variability of mutational spectrum in humans. To test whether the elevated C-to-T rate could be explained by high variability in the white-tailed deer mutation spectrum arising from a small deer sample size, 10 random subsamples of the same size as the delta white-tailed deer dataset (435 mutations each) were selected to estimate spectrum variability, with whiskers indicating standard deviation.

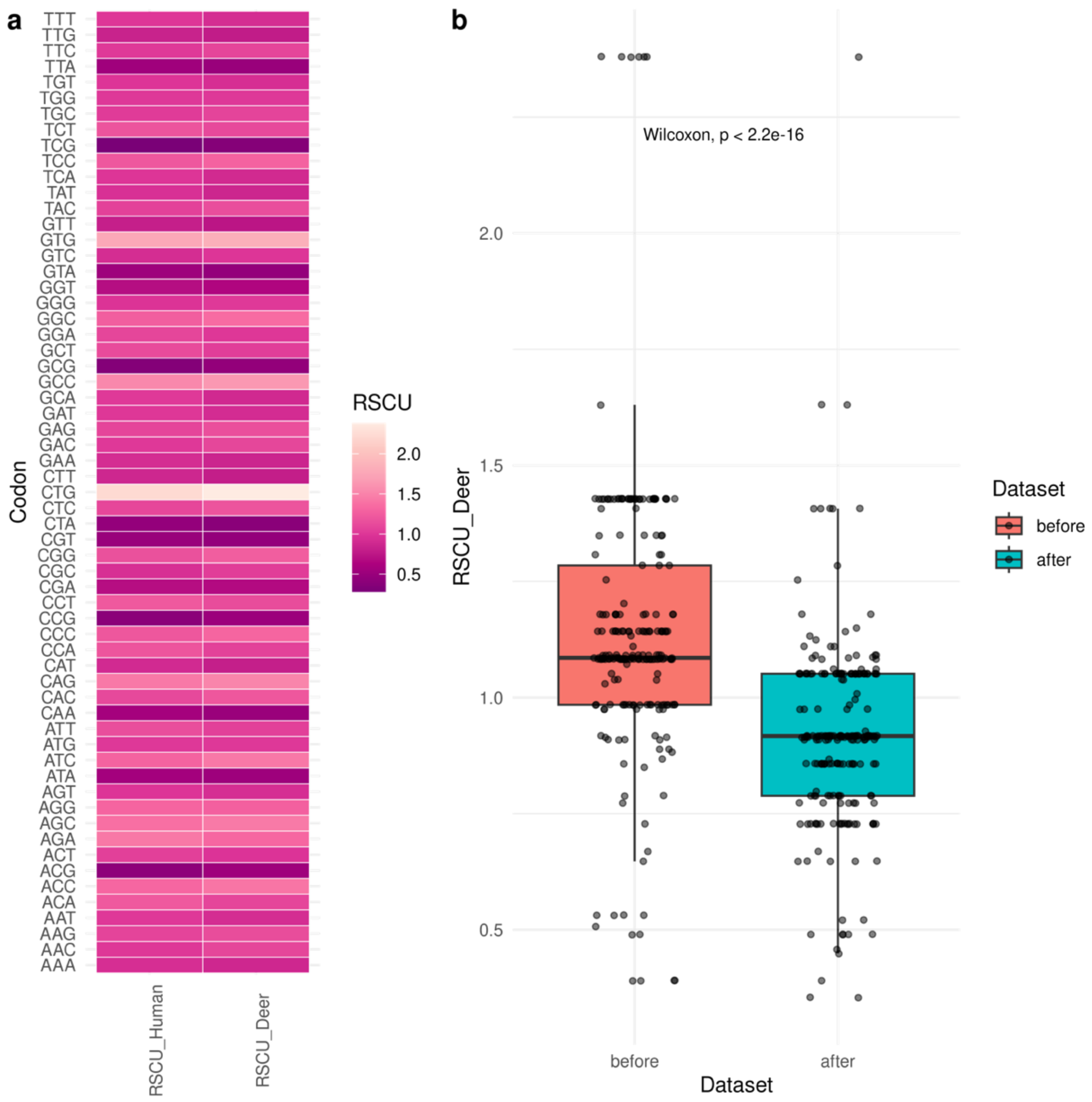

Figure S17. Codon bias in white-tailed deer calculated for synonymous substitutions in Delta white-tailed deer clusters. (a) Comparison of relative synonymous codon usage (RSCU) in human and white-tailed deer. (b) Changes in codon RSCU by introduction of mutation. Distribution of RSCU values in ancestral codons (n=221) (before mutation was introduced) are shown in coral and after – in cyan. Box midlines indicate the median, the box limits show the 75th and 25th quartiles, and the whiskers show the distribution range. RSCU calculated as described in Sharp et al.[2] Information on codon usage in white-tailed deer and humans was obtained from CoCoPUTS database (https://dnahive.fda.gov/dna.cgi?cmd=codon_usage&id=537&mode=cocoputs).

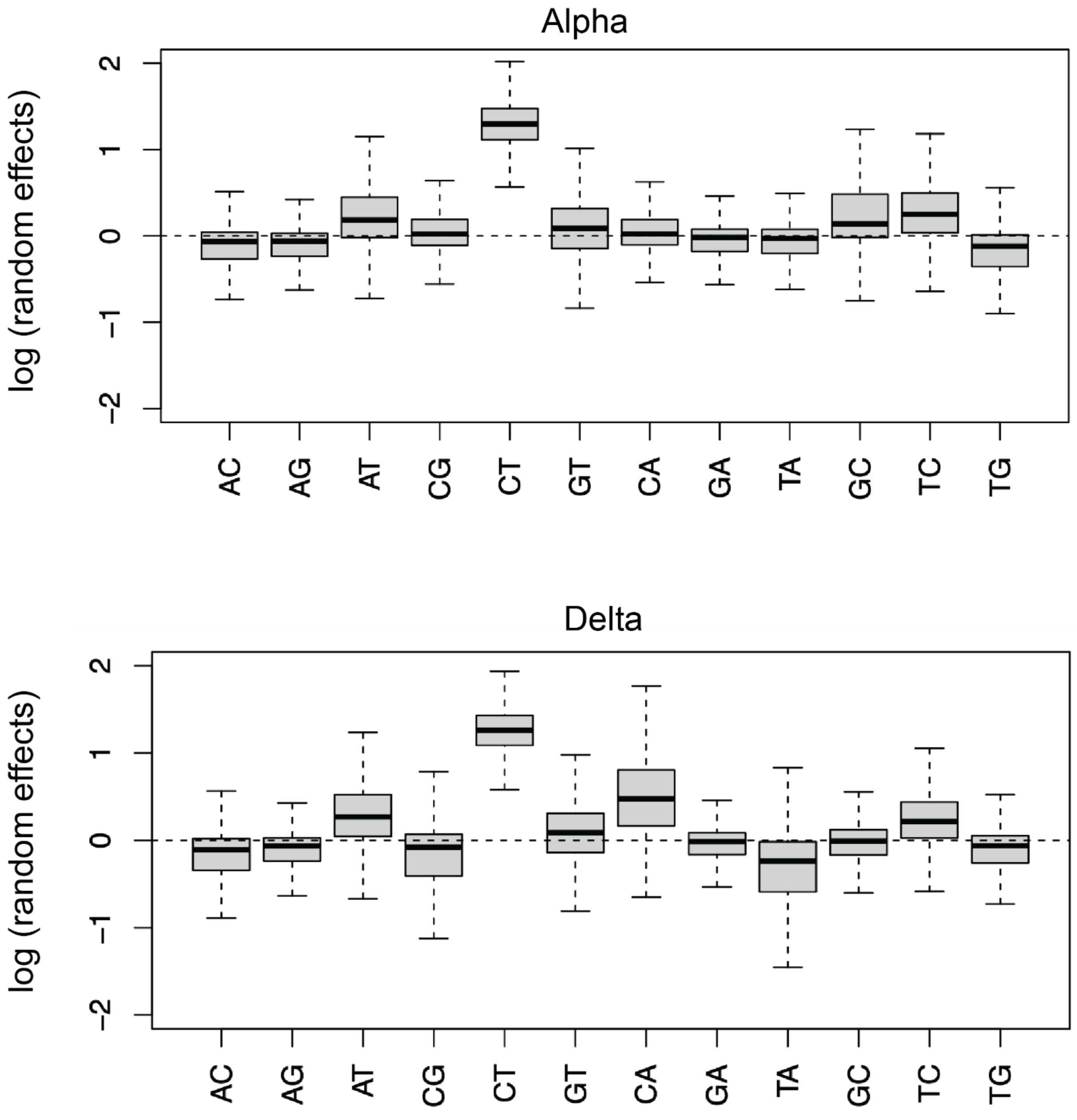

Figure S18. SARS-CoV-2 substitution rates (WTD-to-early human ratio). The log deviation (random-effect) from HKY model relative rates is presented for the ratio of deer viruses (alpha, above, n = 786, and delta, below, n = 1094) compared to a dataset of 786 early human SARS-CoV-2 viruses from Pekar et al.[1] Box midlines indicate the median, the box limits show the upper and lower quartiles, and the whiskers extend to 1.5 times the interquartile range.

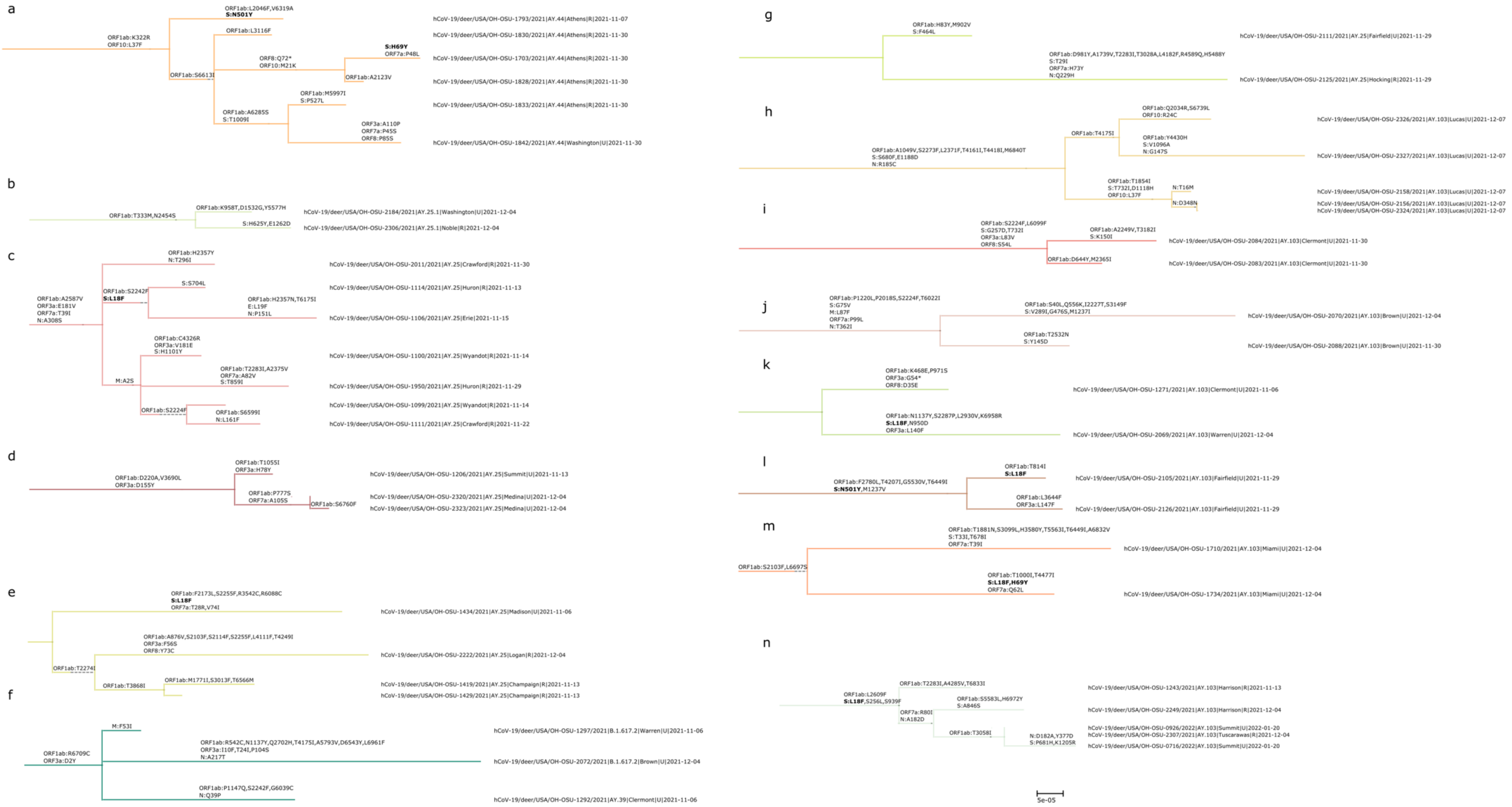

Figure S19. Transmission clusters in white-tailed deer Delta dataset. Missense mutations are shown on the branches. Recurring mutations in Spike with known phenotypic effects are shown in bold.

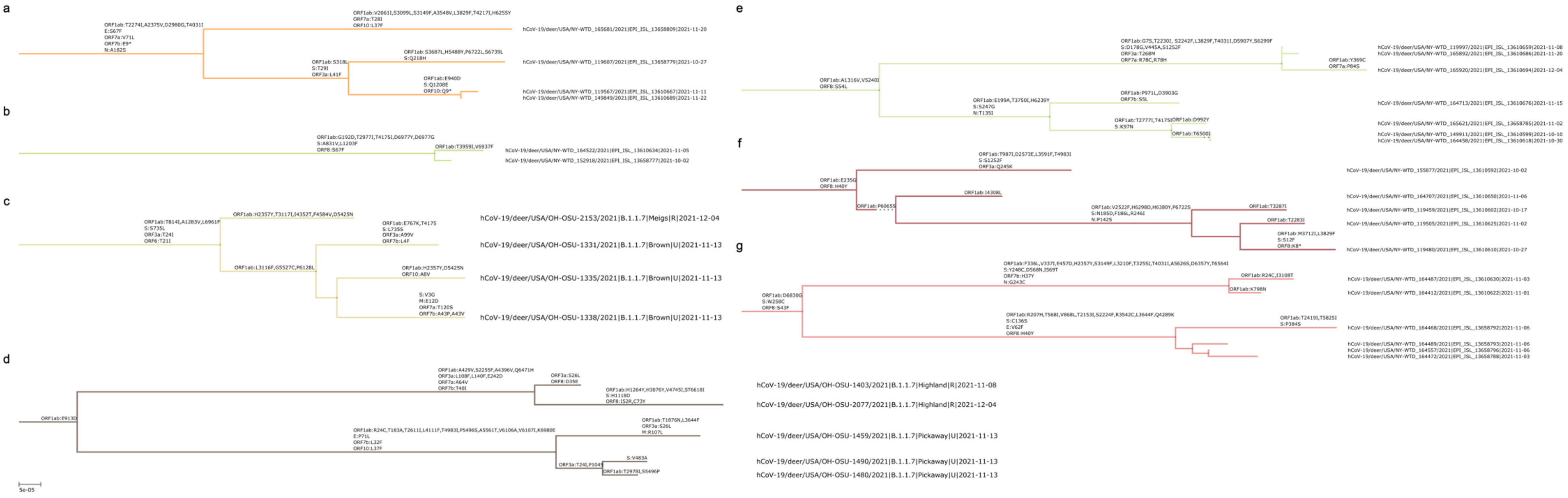

Figure S20. All transmission clusters in white-tailed deer Alpha dataset. (c) and (d) correspond to two clusters found in Ohio, members of which were sequenced in this study, while other clusters correspond to data collected in NY state and was downloaded from GISAID. Missense mutations are shown on the branches. Recurring mutations in Spike with known phenotypic effects are shown in bold.

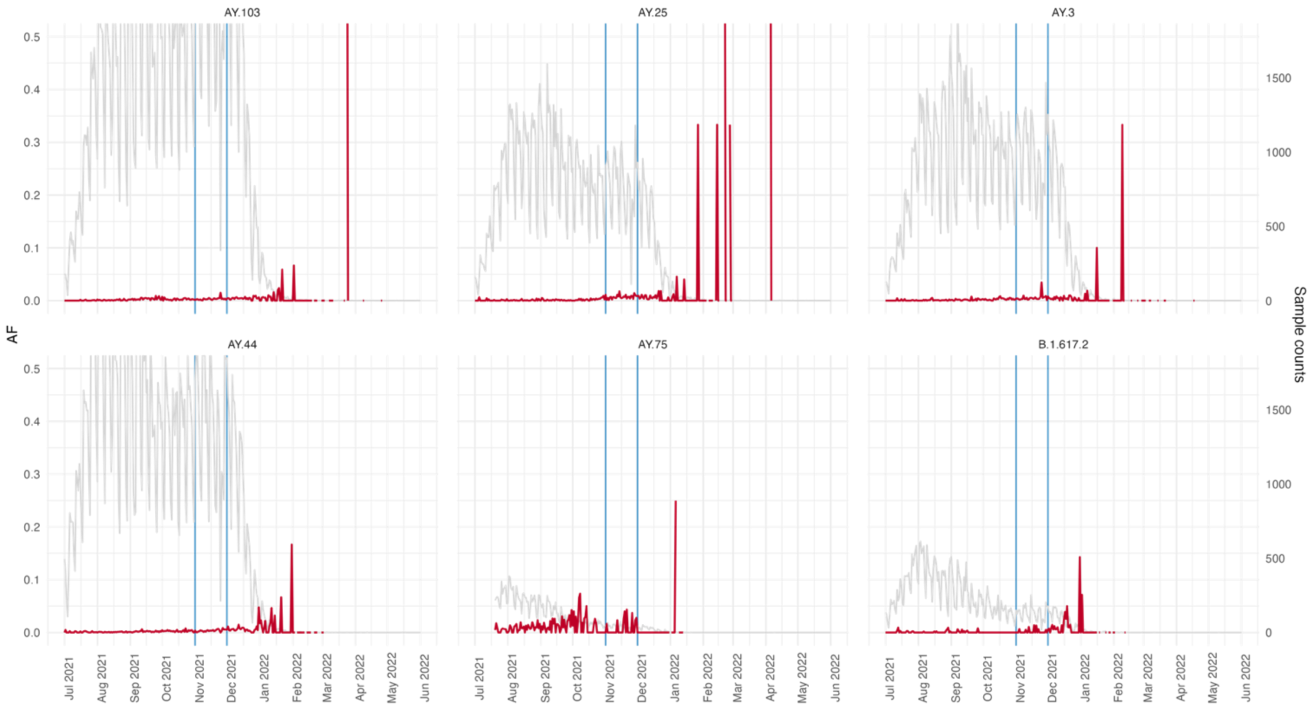

Figure S21. Changes in S:L18F in six Delta lineages in human population. Red line – portion of human samples with mutation among all samples from particular lineage on the particular day. Grey line – total number of samples collected from the lineage on that day. Blue lines show the interval in which samples from white-tailed deer with L18F were collected.

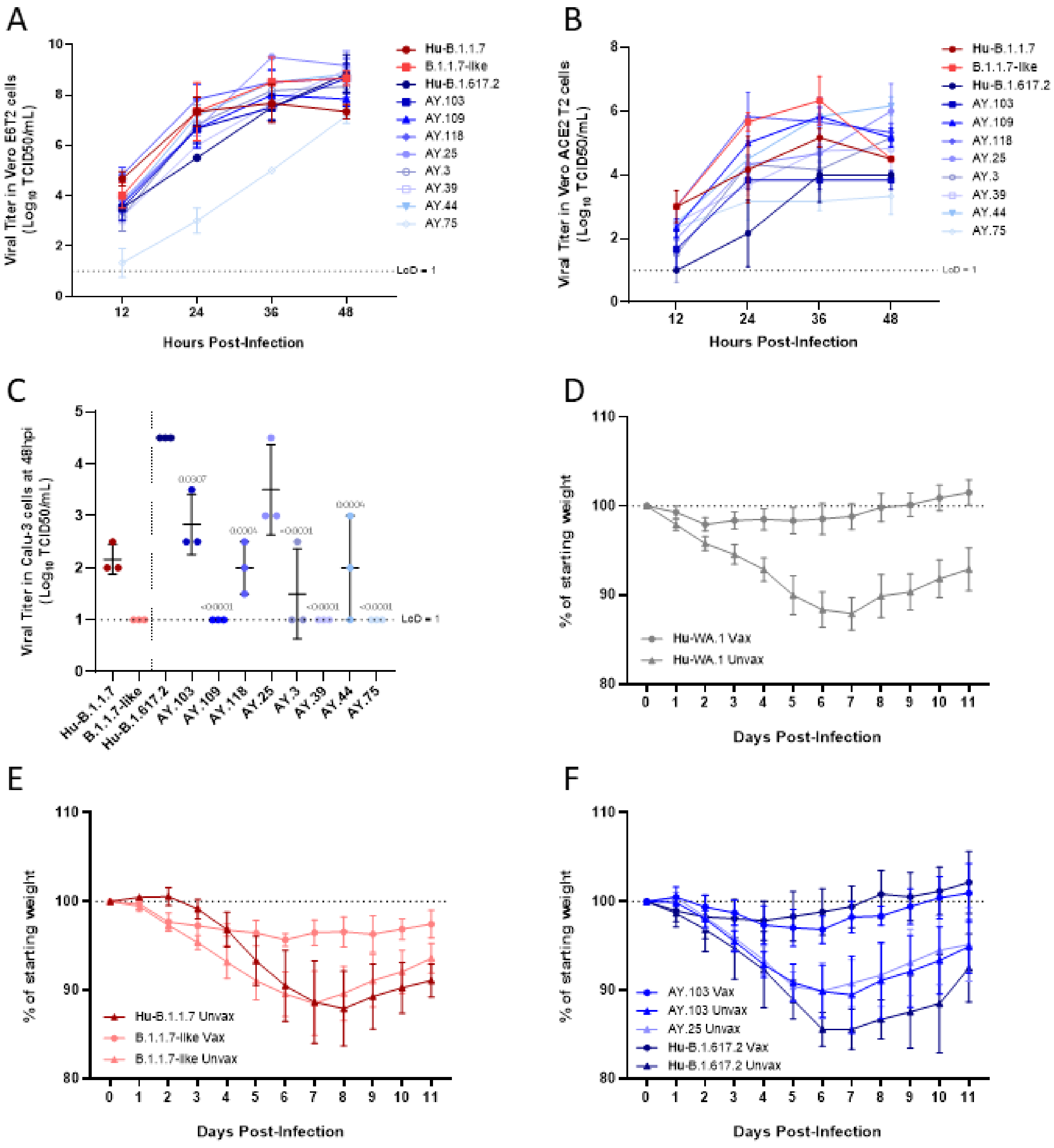

A
Viral Titer in Vero E6T2 cells (Log10 TCID50/mL)
Hours Post-Infection
LoD = 1
Hu-B.1.1.7
B.1.1.7-like
Hu-B.1.617.2
AY.103
AY.109
AY.118
AY.25
AY.3
AY.39
AY.44
AY.75
B
Viral Titer in Vero ACE2 T2 cells (Log10 TCID50/mL)
Hours Post-Infection
LoD = 1
C
Viral Titer in Calu-3 cells at 48hpi (Log10 TCID50/mL)
LoD = 1
0.0307
0.0004
<0.0001
0.0004
Hu-B.1.1.7
B.1.1.7-like
Hu-B.1.617.2
AY.103
AY.109
AY.118
AY.25
AY.3
AY.39
AY.44
AY.75
D
% of starting weight
Days Post-Infection
Hu-WA.1 Vax
Hu-WA.1 Unvax
E
% of starting weight
Days Post-Infection
Hu-B.1.1.7 Unvax
B.1.1.7-like Vax
B.1.1.7-like Unvax
F
% of starting weight
Days Post-Infection
AY.103 Vax
AY.103 Unvax
AY.25 Unvax
Hu-B.1.617.2 Vax
Hu-B.1.617.2 Unvax

Figure S22. Growth analysis in vitro and body weight loss for Syrian hamsters experimentally infected with SARS-CoV-2. (A) Replication kinetics in VeroE6T2 cells. (B) Replication kinetics in VeroE6A2T2 cells. (C) Virus titers in Calu-3 cells at 48-hour post infection. Viral titers expressed as log10 TCID50/mL were plotted with bars representing mean and standard deviation (n = 3). Statistical analysis was performed using one-way ANOVA with multiple comparisons using Tukey post hoc, p-values displayed from Tukey's test statistic, q, which controls for family wise error rate for multiple comparison. (D-F) Body weight loss comparison between unvaccinated and BNT162b2 vaccinated animals. Golden Syrian hamsters were challenged with Hu-WA.1 (unvaccinated n = 12, vaccinated n = 12), Hu-B.1.1.7 (unvaccinated n = 4), B.1.1.7-like (unvaccinated n = 13, vaccinated n = 13), Hu-B.1.617.2 (unvaccinated n = 11, vaccinated n = 12), AY.103 (unvaccinated n = 13, vaccinated n = 13), and AY.25 (unvaccinated n = 10). Animal sample sizes reflect biologically independent animals. Mean weights as a percentage of starting weight were plotted. Error bars represent standard deviation.

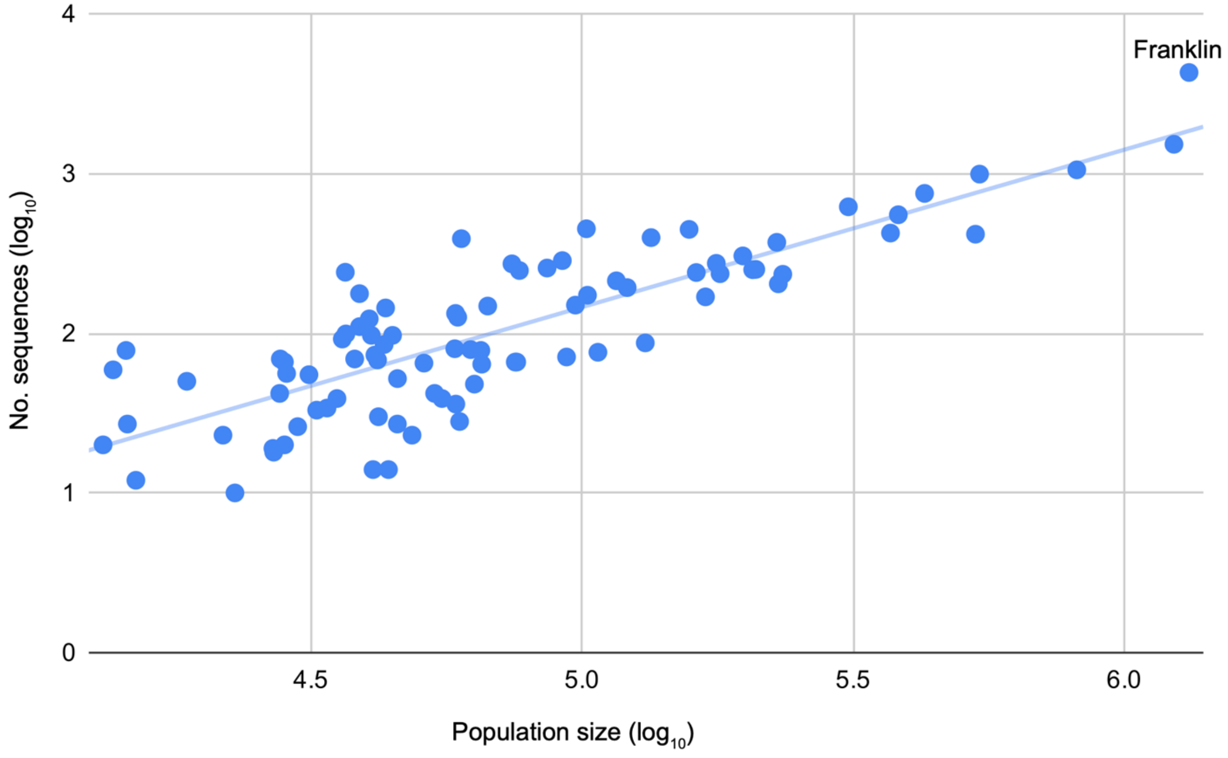

Figure S23. Spatial representation of Ohio SARS-CoV-2 sequences. The number of SARS-CoV-2 sequences an Ohio county that are available from GISAID during the study period (y-axis) is proportional to the population size of that county (x-axis).

Supplementary Materials References

1. Pekar, J. E. *et al.* The molecular epidemiology of multiple zoonotic origins of SARS-CoV-2. *Science* **377**, 960–966 (2022).
2. Sharp, P. M., Tuohy, T. M. F. & Mosurski, K. R. Codon usage in yeast: cluster analysis clearly differentiates highly and lowly expressed genes. *Nucleic Acids Research* **14**, 5125–5143 (1986).
