# Supplementary material for: Accelerated evolution of SARS-CoV-2 in free-ranging white-tailed deer
Source: Nat Commun. 2023 Aug 28;14:5105. doi: 10.1038/s41467-023-40706-y (PMC10462754; doi:10.1038/s41467-023-40706-y)
Supplement: Supplementary file 3 — Description of Additional Supplementary Files [file 41467_2023_40706_MOESM3_ESM.pdf]

Description of Additional Supplementary Files

Supplementary Data 1. All mutations in white-tailed deer clusters for Delta and Alpha datasets.

Supplementary Data 2. Recurrent mutations and mutations with known phenotypes in delta and alpha datasets. Delta clusters include only Ohio white-tailed deer; Alpha clusters include all available white-tailed deer samples.

Supplementary Data 3. GISAID acknowledgement of data contributors table for deer origin SARS-CoV-2 background sequence dataset used in analyses for this study.

Supplementary Data 4. GISAID acknowledgment of data contributors table for human origin SARS-CoV-2 background sequence dataset used in analyses for this study.
