# Supplementary material for: Accelerated evolution of SARS-CoV-2 in free-ranging white-tailed deer
Source: Nat Commun. 2023 Aug 28;14:5105. doi: 10.1038/s41467-023-40706-y (PMC10462754; doi:10.1038/s41467-023-40706-y)
Supplement: Supplementary file 6 — Supplementary Data 3 [file 41467_2023_40706_MOESM6_ESM.pdf]

SUPPLEMENTAL TABLE

Data Availability

GISAID Identifier: EPI_SET_230130nd
doi: 10.55876/gis8.230130nd

All genome sequences and associated metadata in this dataset are published in GISAID's EpiCoV database. To view the contributors of each individual sequence with details such as accession number, Virus name, Collection date, Originating Lab and Submitting Lab and the list of Authors, visit 10.55876/gis8.230130nd

Data Snapshot

- EPI_SET_230130nd is composed of 163 individual genome sequences.
- The collection dates range from 2020-09-28 to 2022-02-11;
- Data were collected in 2 countries and territories;
- All sequences in this dataset are compared relative to hCoV-19/Wuhan/WIV04/2019 (WIV04), the official reference sequence employed by GISAID (EPI_ISL_402124). Learn more at https://gisaid.org/WIV04.
