# Supplementary material for: Accelerated evolution of SARS-CoV-2 in free-ranging white-tailed deer
Source: Nat Commun. 2023 Aug 28;14:5105. doi: 10.1038/s41467-023-40706-y (PMC10462754; doi:10.1038/s41467-023-40706-y)
Supplement: Supplementary file 8 — Reporting Summary [file 41467_2023_40706_MOESM8_ESM.pdf]

nature portfolio

Corresponding author(s): Martha I. Nelson
Andrew S. Bowman

Last updated by author(s): Jul 14, 2023

Reporting Summary

Nature Portfolio wishes to improve the reproducibility of the work that we publish. This form provides structure for consistency and transparency in reporting. For further information on Nature Portfolio policies, see our Editorial Policies and the Editorial Policy Checklist.

Statistics

For all statistical analyses, confirm that the following items are present in the figure legend, table legend, main text, or Methods section.

n/a	Confirmed	
☐	☒	The exact sample size (*n*) for each experimental group/condition, given as a discrete number and unit of measurement
☐	☒	A statement on whether measurements were taken from distinct samples or whether the same sample was measured repeatedly
☐	☒	The statistical test(s) used AND whether they are one- or two-sided<br>*Only common tests should be described solely by name; describe more complex techniques in the Methods section.*
☐	☒	A description of all covariates tested
☐	☒	A description of any assumptions or corrections, such as tests of normality and adjustment for multiple comparisons
☐	☒	A full description of the statistical parameters including central tendency (e.g. means) or other basic estimates (e.g. regression coefficient) AND variation (e.g. standard deviation) or associated estimates of uncertainty (e.g. confidence intervals)
☐	☒	For null hypothesis testing, the test statistic (e.g. *F*, *t*, *r*) with confidence intervals, effect sizes, degrees of freedom and *P* value noted<br>*Give P values as exact values whenever suitable.*
☐	☒	For Bayesian analysis, information on the choice of priors and Markov chain Monte Carlo settings
☒	☐	For hierarchical and complex designs, identification of the appropriate level for tests and full reporting of outcomes
☒	☐	Estimates of effect sizes (e.g. Cohen's *d*, Pearson's *r*), indicating how they were calculated

Our web collection on statistics for biologists contains articles on many of the points above.

Software and code

Policy information about availability of computer code

Data collection	DRAGEN COVID Lineage app v.3.5.6 (Illumina) was used to assemble consensus sequences for original nasal swab specimens. Select viral isolates were sequenced to confirm identity with the original sample sequences that produced the viral isolates using CLC Genomic Workbench v22.0.1 (Qiagen) and BioEdit v7.2.5 (open source). Maps were generated using ArcMap (ESRI). Pangolin was used to assign lineages to SARS-CoV-2 sequences and NextClade and MAFFT v.7.475 were used for alignment. States for mutation analyses were reconstructed at all tree nodes with Tree Time ancestral v 0.9.0-b.2. Extracted mutation vcf files were annotated with SnpEff v 4.5. Mutations in known problematic sites were filtered out using a list available at https://github.com/W-L/ProblematicSites_SARS-CoV2. Observed mutations on the spike trimer were visualized using the Protein Data Bank (PDB; rcsb.org), structure ID 7JJI. Structure visualization was performed with Open-Source PyMol version 2.4.0. R-package Mutational Patterns was utilized to reconstruct mutational contexts. The HyPhy v. 2.5.40 package was used to study positive and negative selection. Foreground branches were marked on the phylogenetic tree with phylotree.js (http://veg.github.io/phylotree.js/#). We used codeml (from PAML version 4.9e) to test whether there are signs of positive selection on branches leading for transmission clusters.

Data analysis	Mixed effects logistic regression was fit in STATA 14.2. Statistical analysis for all in vitro and in vivo experiments was performed in Prism v.9 (GraphPad). IQ-TREE version v1.6.12 (open source) was used to infer phylogenetic trees which were visualized in FigTree v.1.4.4 (open source), deer clusters were confirmed using UShER (open source) downloaded on 2022-07-01 (http://hgdownload.soe.ucsc.edu/goldenPath/wuhCor1/UShER_SARS-CoV-2/2022/07/01/public-2022-07-01.all.masked.pb.gz). BEAST pre-release v.1.10.5 package (open source) compiled from GitHub October 20, 2022, was used to perform the time-scaled Bayesian analysis using the Markov chain Monte Carlo method using the BEAGLE 3 library to improve computational performance and results were visualized in Tracer v.1.7.2 (open source). LogCombiner v1.10.4 (open source) was used to combine runs/trees which was summarized into a single tree by by TreeAnnotator v1.10.4 (open source). Results from mutation analyses were visualized with ggplot2 package for R 4.1.2. Individual transmission clusters were visualized with the ETE 3 python package. Code generated for analysis is available from github at https://github.com/garushyants/sars_cov_2_deer_Ohio.

For manuscripts utilizing custom algorithms or software that are central to the research but not yet described in published literature, software must be made available to editors and reviewers. We strongly encourage code deposition in a community repository (e.g. GitHub). See the Nature Portfolio guidelines for submitting code & software for further information.

Data

Policy information about availability of data

All manuscripts must include a data availability statement. This statement should provide the following information, where applicable:

- Accession codes, unique identifiers, or web links for publicly available datasets
- A description of any restrictions on data availability
- For clinical datasets or third party data, please ensure that the statement adheres to our policy

Whole-genome SARS-CoV-2 sequences are available on GenBank and raw sequence read data are available at NCBI SRA, accession numbers are available in Table S11. Available sequences for background data were downloaded from GISAID (acknowledged in Tables S12-S13). The 787 genomes analyzed by Pekar et al. 2022 were used to compare for rate sensitivity analysis (DOI: 10.1126/science.abp8337). The epidemiological curve of SARS-CoV-2 cases in humans in Ohio was generated using data available from the US Centers for Disease Control and Prevention (https://data.cdc.gov/Case-Surveillance/COVID-19-Case-Surveillance-Public-Use-Data-with-Ge/n8mc-b4w4). Wuhan SARS-CoV-2 genome NC_045512.2 (https://www.ncbi.nlm.nih.gov/nuccore/NC_045512.2) was used as reference for the mutation analysis. Observed mutations on the spike trimer were visualized using the Protein Data Bank (PDB; rcsb.org), structure ID 7JJI (http://doi.org/10.2210/pdb7jji/pdb). All other data are included in this article and its supplementary files.

Research involving human participants, their data, or biological material

Policy information about studies with human participants or human data. See also policy information about sex, gender (identity/presentation), and sexual orientation and race, ethnicity and racism.

Reporting on sex and gender	No human subjects were included in this study.
Reporting on race, ethnicity, or other socially relevant groupings	No human subjects were included in this study.
Population characteristics	No human subjects were included in this study.
Recruitment	No human subjects were included in this study.
Ethics oversight	No human subjects were included in this study.

Note that full information on the approval of the study protocol must also be provided in the manuscript.

Field-specific reporting

Please select the one below that is the best fit for your research. If you are not sure, read the appropriate sections before making your selection.

☐ Life sciences ☐ Behavioural & social sciences ☒ Ecological, evolutionary & environmental sciences

For a reference copy of the document with all sections, see nature.com/documents/nr-reporting-summary-flat.pdf

Ecological, evolutionary & environmental sciences study design

All studies must disclose on these points even when the disclosure is negative.

Study description	This study involved SARS-CoV-2 testing in white-tailed deer followed by epidemiological, virological, and evolutionary analyses of SARS-CoV-2 sequences.
Research sample	Nasal swabs were collected from white-tailed deer carcasses postmortem. Deer were harvested as part of a deer population management programs (801 culled deer) or hunter harvested (713 hunted deer) throughout Ohio and not for the primary purposes of this study. Sample collection was opportunistic and conducted in cooperation with regulatory agencies and as part of routine surveillance programs. Harvested animals included 298 juveniles (under 1 year old) and 1199 adult (1 year or older), 806 males and 709 females. Geographically, we collected samples from 83 of Ohio's 88 counties. By sampling deer from all over Ohio, we

represented deer that use several major habitat types including metropolitan areas, national forest/national park, and rural agricultural land. Deer were selected for study due to their frequent and widespread infection with SARS-CoV-2 and the public health risk associate with establishment of an animal reservoir.

Sampling strategy	Agency partners were trained in nasal swab, blood sample, and data collection methods and then materials required for sample collection were distributed to all districts around Ohio. Samples were collected at meat processing, taxidermy, and/or wildlife check stations where deer hunters bring carcasses during the hunting season. Agencies already place personnel at these locations to record deer harvest data (such as age, sex, and potential collect CWD samples), and our SARS-CoV-2 samples were collected opportunistically from every deer for which collection was feasible. Additionally, we partnered with several population management programs in which deer are culled to reduce the population numbers. Again, sample collectors were trained and stationed at those management program central processing sites where carcasses are brought, and samples were collected opportunistically. No statistical sample size calculation was completed, we collected samples from as many deer as possible and attempted to cover the entire state geographically.
Data collection	Sample collection materials and training were overseen by Dillon McBride, Steven Overend, Jacqueline Nolting, Michael Tonkovich, Tyler Genders, Andrew Montoney, Kevin Kasnyik, and Andrew Bowman. Many individuals were trained in and aided with sample collection including but not limited to those listed in the acknowledgments. Data were recorded on paper data sheets which were returned to Andrew Bowman's laboratory at The Ohio State University along with samples.
Timing and spatial scale	All samples were collected between October 2021-March 2022 from across the entire US state of Ohio. This time period was selected because it corresponds with the white-tailed deer hunting season which allowed for opportunistic sample collection.
Data exclusions	SARS-CoV-2 sequencing was only attempted for samples with RT-PCR cycle threshold values of 33 or lower. This was a predetermined exclusion criteria to not waste resources on samples that do not typically have enough viral material for successful sequencing. Viruses selected for experimental characterization were limited to samples from which we were able to isolate virus, and were further selected to represent lineages that we detected. 1 laboratory animal (Syrian hamster) was excluded from the study when it was determined to be diabetic and euthanized prior to the infection study.
Reproducibility	All deer nasal swabs were initially tested using the Charité/Berlin (WHO) assay targeting the E gene with an added internal positive control to validate our RNA extraction and PCR reactions. If the E assay was positive, it was followed by confirmatory gene targets including RdRp or N1 and N2. Samples must be positive during the screen and confirmatory testing to be considered positive. Samples were tested serologically in at least duplicate. Select viral isolates were sequenced to confirm identity with the original sample sequences that produced the viral isolates. Animal model experiment groups each had at least 3 animals at every time point. All attempts to repeat the experiments were successful.
Randomization	As SARS-CoV-2 infection status was unknown in all deer prior to testing, all samples were extracted in batches without any specific efforts toward randomization. Randomization was not performed or not applicable for in vitro experiments. For In vivo experiments, research animals were randomly assigned to experimental groups by animal care staff.
Blinding	SARS-CoV-2 infection status was unknown in all deer prior to testing. ABSL3+ studies were not blinded. Institutional biosafety guidelines require the investigators to be aware of the identity of the virus associated with the animals and samples they are using, as well as the elevated risks associated with such samples. Serum microneutralization results were analysed and scored by individuals blinded to the sample identification until after the data were completed.

Did the study involve field work? ☒ Yes ☐ No

Field work, collection and transport

Field conditions	Samples were collected from Ohio (USA) during the autumn and winter months. Sample collection most commonly occurred in or immediately adjacent to a structure that provided cover from wind and rainfall.
Location	All deer came from across the entire state of Ohio (USA)
Access & import/export	Deer were harvested as part of deer population management programs or the regularly occurring civilian hunting season in Ohio and not for the purposes of this study. Sample collection was opportunistic and conducted in cooperation with regulatory agencies and as part of routine surveillance programs. Samples were collected postmortem which was deemed exempt by the Ohio State University Institutional Animal Care and Use Committee.
Disturbance	No additional disturbances were made for the purpose of this study.

Reporting for specific materials, systems and methods

We require information from authors about some types of materials, experimental systems and methods used in many studies. Here, indicate whether each material, system or method listed is relevant to your study. If you are not sure if a list item applies to your research, read the appropriate section before selecting a response.

Materials & experimental systems

n/a	Involved in the study	
☐	☒	Antibodies
☐	☒	Eukaryotic cell lines
☒	☐	Palaeontology and archaeology
☐	☒	Animals and other organisms
☒	☐	Clinical data
☒	☐	Dual use research of concern
☒	☐	Plants

Methods

n/a	Involved in the study	
☒	☐	ChIP-seq
☒	☐	Flow cytometry
☒	☐	MRI-based neuroimaging

Antibodies

Antibodies used: Rabbit anti-SARS CoV-2 NP mAb (Sinobiologicals Cat # 40143-R004, clone #004); goat anti-rabbit IgG –HRP conjugated antibody (Cell Signaling Cat# 7074S)

Validation: From the vendor : 40143-R040 Has cross-reactivity in ELISA with SARS-CoV-2 Nucleocapsid protein. No cross-reactivity in ELISA with MERS-CoV, HCOV-229E, HCoV-NL63, HCoV-HKU1, HCoV-OC43

Eukaryotic cell lines

Policy information about cell lines and Sex and Gender in Research

Cell line source(s): Calu3 was sourced from American Type Culture Collection (ATCC); VeroE6/TMPRSS2 was sourced from National Institutes of Biomedical Innovation, Health and Nutrition JCRB Cell Bank https://cellbank.nibiohn.go.jp/english/; VeroE6/TMPRS2/T2A/ACE2 was sourced from Dr. Barney Graham at VRC, NIAID, NIH, currently available at BEI Resources (Cat # NR-54970)

Authentication: The cell lines used in this study were not authenticated after being received. Each was received with documentation including cell type, clone, lot, passage, morphology, species determination, viability and growth properties.

Mycoplasma contamination: All cell lines were tested negative for mycoplasma by routine testing using ATCC Universal Mycoplasma testing kit (30-1012k) during laboratory culture.

Commonly misidentified lines (See ICLAC register): No commonly misidentified cell lines were used in the study.

Animals and other research organisms

Policy information about studies involving animals; ARRIVE guidelines recommended for reporting animal research, and Sex and Gender in Research

Laboratory animals: Male LVG Golden Syrian Hamster, 4-5 weeks of age, were purchased from Charles River Laboratories (Wilmington, MA).

Wild animals: Wild whit-tailed deer were harvested as part of deer population management programs and the civilian hunting season in Ohio and not for the purposes of this study. Sample collection was opportunistic from these harvested wild deer and was conducted postmortem.

Reporting on sex: Laboratory experiments were performed exclusively with male animals, as designated by the vendor prior to arrival. Male animals were chosen as it has been established male Syrian hamsters are more susceptible to disease caused by SARS-CoV-2 Nature (Doi: 10.1038/s41586-020-2787-6).

Field-collected samples: Culled deer carcasses were transported to a central processing point in the field and hunted deer carcasses were brought by hunters to local check stations or processing facilities where samples were collected under field conditions. Sample collectors wore gloves and a face mask. A nasal swab was collected from each deer and placed into a tube with BD viral transport media, which is shelf stable prior to use. After collection, samples were placed at 4°C for immediate transport and then transferred into a −80°C freezer where they remained until testing was initiated.

Ethics oversight: Laboratory animal studies were conducted in accordance with the Guide for the Care and Use of Laboratory Animals of the National Institutes of Health and approved under St. Jude Children's Research Hospital's Animal Care and Use Committee protocol 442. Sample collection from deer was conducted postmortem which was deemed exempt by the Ohio State University Institutional Animal Care and Use Committee.

Note that full information on the approval of the study protocol must also be provided in the manuscript.
